# Genome mining and characterisation of a novel transaminase with remote stereoselectivity

**DOI:** 10.1038/s41598-019-56612-7

**Published:** 2019-12-30

**Authors:** D. P. Gavin, F. J. Reen, J. Rocha-Martin, I. Abreu-Castilla, D. F. Woods, A. M. Foley, P. A. Sánchez-Murcia, M. Schwarz, P. O’Neill, A. R. Maguire, F. O’Gara

**Affiliations:** 1 School of Chemistry; Analytical and Biological Chemistry Research Facility, University College Cork, Cork, Ireland; 2 Synthesis and Solid State Pharmaceutical Centre, University College Cork, Cork, Ireland; 3 BIOMERIT Research Centre, School of Microbiology, University College Cork, Cork, Ireland; 4 School of Microbiology, University College Cork, T12 K8AF Cork, Ireland; 5 School of Chemistry, School of Pharmacy, Analytical and Biological Chemistry Research Facility, University College Cork, Cork, Ireland; 6 Institute of Theoretical Chemistry, Faculty of Chemistry, University of Vienna, Währinger Str. 17, A-1090 Vienna, Austria; 7 Pfizer Process Development Centre, Loughbeg, Cork, Ireland; 8 Human Microbiome Programme, School of Pharmacy and Biomedical Sciences, Curtin Health Innovation Research Institute, Curtin University, Perth, WA 6102, Australia and Telethon Kids Institute, Perth, WA 6008 Australia

**Keywords:** Biotechnology, Chemistry

## Abstract

Microbial enzymes from pristine niches can potentially deliver disruptive opportunities in synthetic routes to Active Pharmaceutical Ingredients and intermediates in the Pharmaceutical Industry. Advances in green chemistry technologies and the importance of stereochemical control, further underscores the application of enzyme-based solutions in chemical synthesis. The rich tapestry of microbial diversity in the oceanic ecosystem encodes a capacity for novel biotransformations arising from the chemical complexity of this largely unexplored bioactive reservoir. Here we report a novel ω-transaminase discovered in a marine sponge *Pseudovibrio* sp. isolate. Remote stereoselection using a transaminase has been demonstrated for the first time using this novel protein. Application to the resolution of an intermediate in the synthesis of sertraline highlights the synthetic potential of this novel biocatalyst discovered through genomic mining. Integrated chemico-genomics revealed a unique substrate profile, while molecular modelling provided structural insights into this ‘first in class’ selectivity at a remote chiral centre.

## Introduction

Biocatalysis has emerged as an important technology in the ‘toolkit’ of the asymmetric chemist, especially in the last decade^1–4^. The integration of biocatalytic steps in syntheses can have positive implications for the environmental impact of a process but it can also alter synthetic routes, since exploiting the chemo-, regio- and stereoselectivity of biocatalysts can open the door to alternative, shorter syntheses^5,6^. Chiral amines, and functional groups derived from amines, are found in many pharmaceuticals and fine chemicals^7^. These stereogenic centres are often embedded in complex structures. The Active Pharmaceutical Ingredient (API), or target molecule, often contains at least one other stereocentre, and controlling the overall stereochemistry is challenging. Despite numerous advances in organic synthesis over the last number of decades, classical resolution remains a reliable and prominent technique in pharmaceutical manufacture^8,9^. Due to their prevalence in pharmaceuticals and fine chemicals, chiral amine subunits are high value synthetic targets and biocatalysis represents a highly enantioselective and sustainable route to them. Indeed, many elegant biocatalytic routes to chiral amines have been reported utilising a range of enzymatic families, including hydrolases, monoamine oxidases, imine reductases and reductive aminases in the (dynamic) kinetic resolution and asymmetric synthesis of pharmaceutical intermediates^10–20^.

Transaminases have received much attention in the context of chiral amine synthesis^21–25^. *ω*-Transaminases (*ω-*TAs, E.C. 2.6.1.X) are pyridoxal-5′-phosphate (PLP)-dependent enzymes that catalyse the reversible transfer of an amine group from a donor molecule (*e.g*. alanine) to an acceptor molecule containing a carbonyl group. Structurally, *ω-*TAs are typically dimeric proteins which have a large and small binding pocket within their active site, although tetrameric forms have been reported^26^. Almost without exception, the small pocket can accommodate only a methyl group or a quasi-methyl group such as a -CH_2_- within the framework of a cyclohexyl or cyclopentyl moiety. As such, a major limitation of these enzymes is their limited substrate scope^27^. However, their synthetic power has ensured that they have become valuable tools in the synthesis of stereogenically pure primary amines^28,29^. The challenge therefore is to discover or engineer transaminases with unique biocatalytic properties such that stereo-specific resolution of a broader range of substrates is feasible.

The marine ecosystem sustains a rich microbial biodiversity, the biocatalytic potential of which is largely unexplored when compared with its terrestrial counterpart. Studies reporting the isolation of novel bioactive compounds from the marine environment have become more prominent in recent years with anti-cancer, anti-microbial, and anti-inflammatory therapeutics being among the most well studied^30^. It is becoming increasingly apparent that the functional novelty encoded within the rich genetic diversity of marine polymicrobial communities extends also to the enzymatic activities that sustain microbial life in this ecosystem. While technologies such as directed evolution and rational design have provided further added value to existing enzyme frameworks, identifying source proteins with novel functionality has several benefits, particularly where previously pristine niches have revealed their rich and biodiverse bioactive potential^31^. There are several routes currently available to access these novel activities, from high-throughput metagenomics based functional screens, to more targeted genome mining approaches based on a fundamental understanding of process and structure. In many cases, approaches that utilise multiple components of the genome technology toolkit are favoured. Within the marine niche, a significant concentration of bioactive potential resides within the microbial communities that colonise marine sponges of the Porifera phylum^30^. Accessing culturable microbes from marine sponges remains challenging, although several species have emerged with interesting bioactive and biosynthetic profiles. They are an untapped source of proteins with novel favourable functionality and/or substrate scope based on the unique biotransformations required of them e.g. accessing halogenated metabolites. These enzymes could also be expected to exhibit additional properties such as salt and osmotic tolerance offering robustness in synthetic bioreactor pipelines^31,32^.

**Figure 1:**
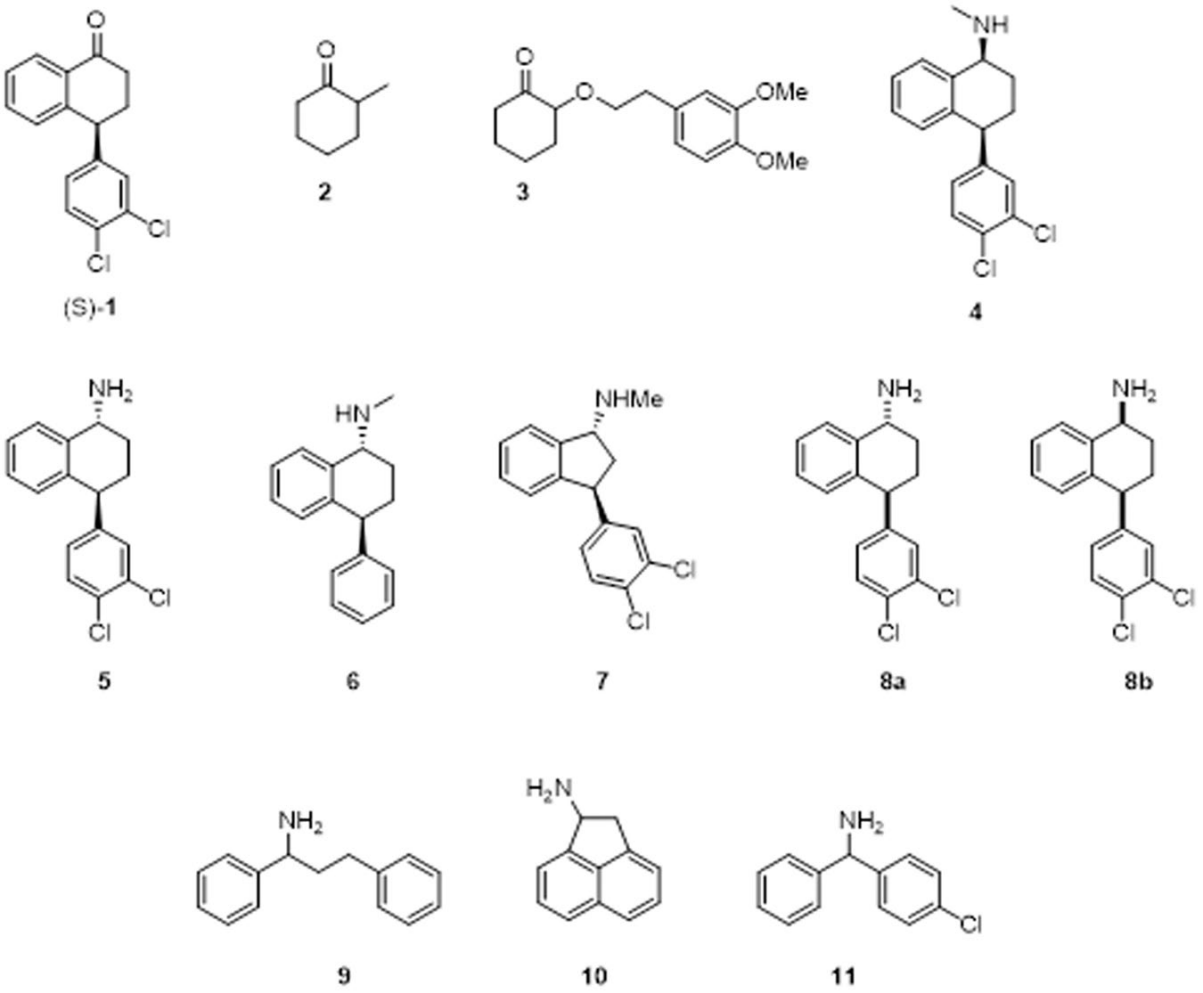
Structures of compounds of interest for transaminase mediated resolution.

Most biocatalytic transformations focus on enantioselection at or near to the site of reaction. There are some examples of biocatalytic resolution of remote stereogenic centres, for example using hydrolases^33–39^. In contrast, transaminase-mediated resolution of compounds bearing even a second stereocentre are very rare. To the best of our knowledge there has been only two such reports and both of these studies were conducted on compounds with a stereogenic centre at the α-carbon (adjacent to the reactive site), **2** and **3**, taking advantage of the enol equilibrium in a dynamic process (Fig. 1)^40,41^.

Sertraline **4 (**Fig. 1**)** is a Selective Seretonin Reuptake Inhibitor (SSRI) which is produced by Pfizer and it is one of the most prescribed antidepressants in the world. Along with a (1 *S*)-secondary amine, sertraline contains a stereogenic centre at the 4-position of the aminotetralin core. The aminotetralin bicycle is a privileged subunit in drug discovery, also being present in dasotraline (**5**) and the deschloro (1 R)-epimer of sertraline, tametraline (**6**). Dasotraline is the (1 *R*)-primary amine equivalent of sertraline and is also a compound of therapeutic interest^42^. Desmethylsertraline (norsertraline) **8b** is the active metabolite of sertraline and the API can be synthesised from this primary amine precursor. Indatraline **7** is a nonselective monoamine reuptake inhibitor^43^.

The key intermediate in the synthesis of sertraline itself is the (4 *S*)-tetralone **1** (Fig. 1). *rac*-**1** Can be accessed *via* the reaction of 1-napthol and 1,2-dichlorobenzene in the presence of a strong Lewis acid^44^. Reductive amination with methylamine yields the product in a 95:5 *cis:trans* ratio^45^. Due to the importance of the API, many syntheses have been reported^44,46–48^. Despite these efforts, an environmentally friendly, operationally simple method of acquiring (*S*)-**1** remains an important synthetic goal.

Here we report the first isolation and characterisation of a ω-transaminase with selectivity at a remote chiral centre. A domain-oriented genome mining search of culturable marine sponge isolates identified a transaminase with a unique sequence compared to existing databases. Substrate profiling revealed that transaminase from *Pseudovibrio* sp. WM33 exhibited an acceptance of bulky substrates, including amine **8b**. Remote stereoselectivity was validated and shown to be a characteristic of the marine transaminase, but not of the well-characterised *Chromobacterium violaceum* transaminase. Molecular modelling provided insights into the structural basis of this selectivity and to our knowledge previously unreported enantiodiscrimination at the remote stereocentre in the biotransformation of amine **8b**.

## Results

### Isolation of a novel ω-transaminase activity from marine sponge *Pseudovibrio* sp WM33

**Figure 2:**
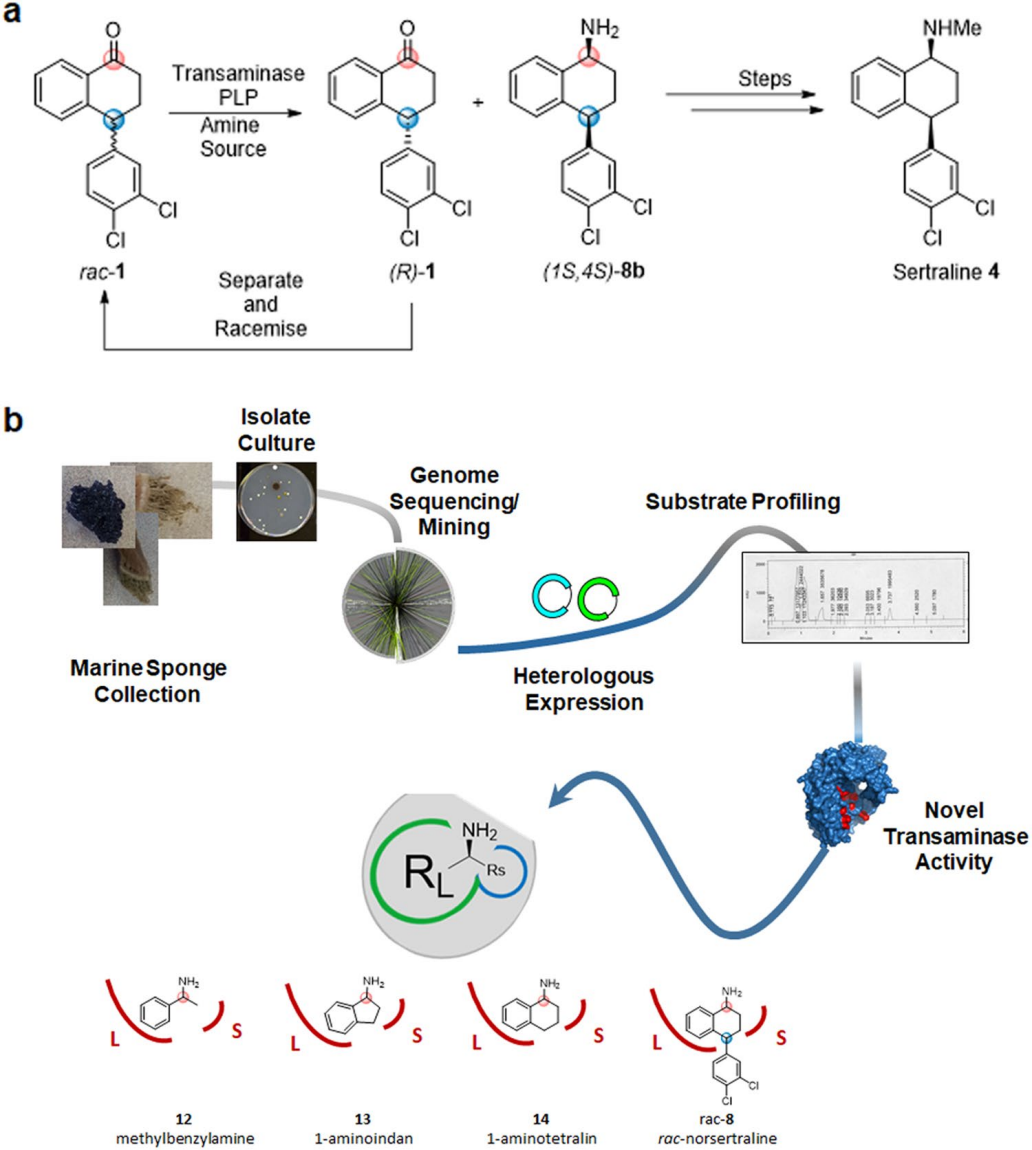
(**a)** Potential resolution of the tetralone intermediate remote stereocentre in the synthesis of sertraline. (**b**) Schematic overview of the pipeline approach for the isolation of ‘first in class’ transaminase activity.

Having envisioned a transaminase-mediated resolution of the sertraline intermediate (Fig. 2a) and inspired by the elegant work of Bornscheuer and co-workers on transaminase evolution towards bulky substrates^49^, we set about the task of finding a transaminase with synthetically useful activity towards interconversion of **1** and **8b**. Importantly, we noted that in Bornscheuer’s study, low levels of activity were detected with several in-house wild-type transaminases and mutant variants against compounds **9** and **10**, while the diaryl compound **11**, a “bulky-bulky” diaryl substrate, was not accepted by any transaminase tested. Given that fold type I transaminases can have activity against 1-aminotetralin **14** and 1-aminoindan **13** (Fig. 2)^41,50–52^, with in some cases higher activity than against the benchmark compound *a*- methylbenzylamine **12**, we postulated that this type of transaminase could in theory bestow remote selectivity in the resolution of substrates bearing a second stereocentre. We theorised that we could find this activity with a fold class I transaminase as the bulky dichlorophenyl-substitution is “away” from the small binding pocket, at the 4-position of the aminotetralin core (Fig. 2).

**Figure 3:**
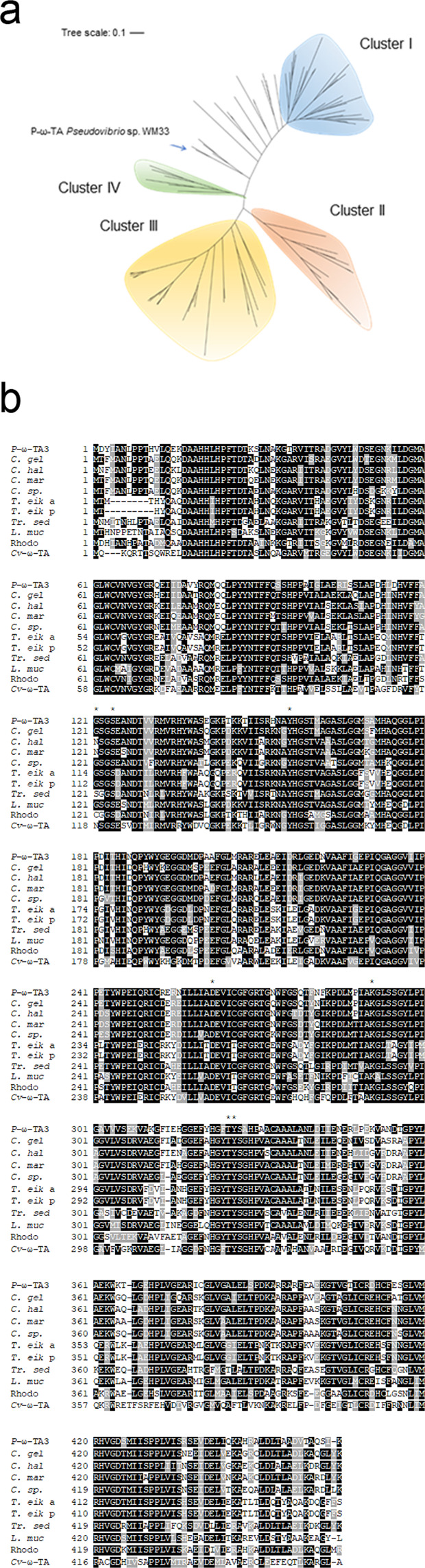
(**a)** Clustering tree analysis of transaminase protein sequences. Transaminase sequences clearly group into four distinct clusters, I-IV. An aminotransferase from *Pseudovibrio* WM33 and *Pseudovibrio* axinellae were found between Cluster I and Cluster IV, these ω-transaminases from *Pseudovibrio* do not group within these cluster due to low identities with other transaminases. The closest aminotransferase to the *Pseudovibrio* transaminases is from *Cohaesibacter marisflavi* with 74% sequence identity. The tree is drawn to scale, with branch lengths measured in the number of substitutions per site. The analysis involved 104 amino acid sequences and all positions containing gaps and missing data were eliminated. A total of 436 positions were contained in the final dataset. Identifiers are as follows: *C. gel*, *Cohaesibacter gelatinilyticus*; *C. hal*, *Cohaesibacter haloalkalitolerans*; *C. mar*, *Cohaesibacter marisflavi*; *C*. sp.*, Cohaesibacter* sp. *ES.047*; *T. eik* a, *Thiothrix eikelboomii* aspartate aminotransferase; *T. eik* p, *Thiothrix eikelboomii* putrescine aminotransferase; *Tr. sed*, *Tropicimonas sediminicola*; *L. muc*, *Leucothrix mucor*; Rhodo, Rhodobacteraceae bacterium). **b** Alignment of *P*-ω-TA with related transaminases including *Cv*-ω-TA. Amino acids strongly conserved are highlighted in black whereas residues less conserved are shown in grey shading. Alignment was performed with T-Coffee and processed using BoxShade. Key active site residues are highlighted with an *^53^.

The genomes of marine sponge isolates were investigated for genes encoding for potential transaminase activity, with particular emphasis on the class 1 fold domain. Presumptive ‘hits’ were identified in several marine sponge isolate genomes, including in the culturable isolate *Pseudovibrio* WM33 (GenPept Accession No. WP_063301853). The sequence encoding the putative transaminase was predicted to encode for a 445 amino acid protein with a predicted molecular weight of 49.8 kDa. Sequence analysis using the BLASTN and BLASTP algorithms revealed a novel sequence compared with existing sequences in the NCBI database. Cluster tree analysis (Fig. 3) demonstrated that the transaminase enzymes grouped into four discrete clusters, defined as Cluster I-IV. Interestingly, *Pseudovibrio* WM33 transaminase sequence branched distinct from all the major transaminase clusters. An alignment was subsequently performed using the *Pseudovibrio* WM33 transaminase (hereafter termed *P*-ω-TA) with the nine non-*Pseudovibrio* ‘best hits’ exhibiting the highest sequence identity from the BLASTP analysis. The well characterised *Chromobacterium violaceum* transaminase (*Cv*-ω-TA), which shares 56% identity to *P*-ω-TA, was also included in the alignment. This transaminase is in the same fold class as *P*-ω-TA. Moreover, the active sites within *Cv*-ω-TA are conserved with the novel *P*-ω-TA, with the exception of Asn 118′ (G121 in *P*-ω-TA)^53^. The amino acids Ser 124′ (S121 in *Cv*-ω-TA) and Tyr 156′ (Y153 in *Cv*-ω-TA) which are involved in forming hydrogen bonds with phosphate oxygen atoms of Pyridoxal 5′-Phosphate (PLP) are present in *P*-ω-TA. Several amide nitrogen atoms, such as those of Thr 325′ and Tyr 326′ (T321 and Y322 in *Cv*-ω-TA), take part in hydrogen bonding with PLP. The amino acid Lys 291′ (K288 in *Cv*-ω-TA), which covalently binds PLP, is also conserved in *P*-ω-TA, as is Asp262′ (D259 in *Cv*-ω-TA), which plays an important role when forming bonds with PLP. However, significant differences and unique regions were identified in the comparative analysis, suggesting the possibility of unique functionality when compared with the extensively used *Cv*-ω-TA.

### Heterologous expression of *P*-ω-TA and validation of transaminase activity

A molecular approach was undertaken to express the putative transaminase encoding gene in *E. coli* BL21 DE RIPL. Gene specific primers were used to generate a *P*-ω-TA amplicon and directional cloning was undertaken to successfully generate an in-frame N-terminal His-Tag *P*-ω- TA fusion (pET- *P*-ω-TA) in the pET28a expression plasmid. Sequence analysis confirmed the fidelity of the inserted DNA, and SDS PAGE analysis confirmed the production of a ~50 kDa protein in both *P*-ω-TA and Cv-ω-TA (Supplementary Data Fig. S1). Lysate from IPTG-induced pET- *P*-ω-TA expressed in *E. coli* BL21 DE RIPL cells was subsequently used to confirm and characterise the transaminase activity of *P*-ω-TA. Lysate from similarly induced *E. coli* BL21 DE RIPL cells carrying an empty plasmid was included in each assay to establish a baseline control.

#### Enantioselectivity

*P*-ω-TA was tested for enantioselectivity using a standard methyl-benzylamine (MBA) substrate. As predicted for class I fold transaminases, purified *P*-ω-TA protein showed *(S)*-enantioselectivity for MBA. Conversion of substrate to acetophenone was observed when *(S)-α-*MBA was used, whereas no acetophenone was produced when the *(R)-α-*MBA version was used (Supplementary Data Fig. S2).

#### Temperature and pH

The effect of temperature and pH on enzyme activity during the reaction was measured using *(S)*-MBA as amino donor with pyruvate providing the ketone group. This revealed an optimum temperature and pH of 40°C and 10, respectively. Total loss of activity was recorded at temperatures above 60°C (Supplementary Data Fig. S2). *P*-ω-TA exhibited measurable activity in the basic pH range of 8–11, being highest at pH 10, perhaps a reflection of its oceanic origin (Supplementary Data Fig. S2).

#### Solvent tolerance

A range of DMSO concentrations were tested to investigate solvent tolerance of the P-ω-TA enzyme. As above, (S)-MBA as amino donor with pyruvate providing the ketone group. The optimum reaction conditions were observed at 10% DMSO, with a dramatic loss in activity at higher, and indeed lower concentrations (Supplementary Data Fig. S3).

### *P*-ω-TA shows no unusual activity towards model substrates compared to *Cv*- ω-TA

**Figure 4:**
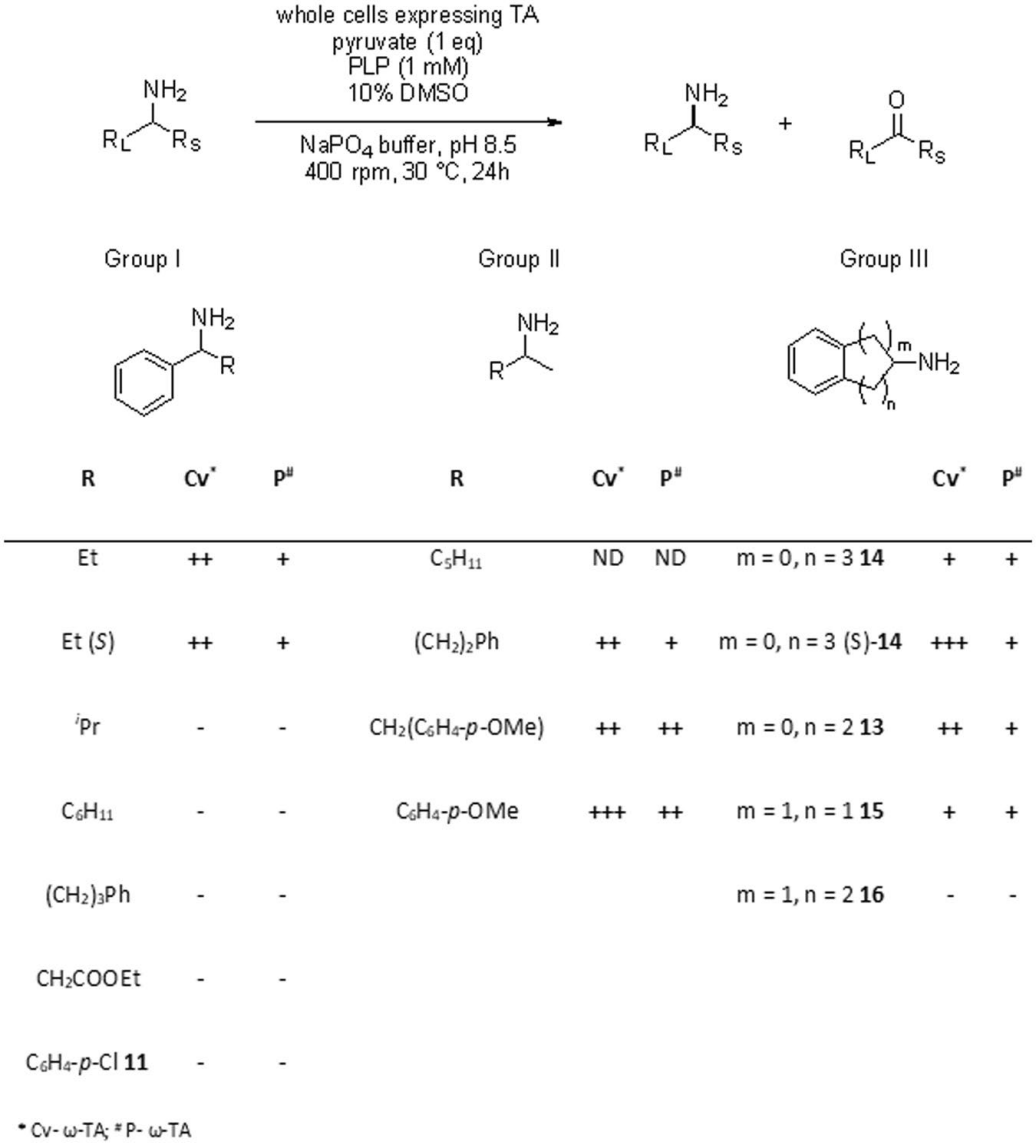
Substrate profiling of P-ω-TA against a panel of substrates grouped based on physical and structural characteristics. Conversion: (+++) > 30%, (++) 10–30%, (+) 0–10%, (−) no conversion; ND – not detected (no starting material or product was recovered from the reaction mixture). Duplicate and triplicate independent biological replicate assays were performed for Group II and Group III, respectively.

A range of model substrates were tested against the novel biocatalyst *P*-ω-TA and, for comparison, the well-characterised *Cv*-ω-TA (heterologously expressed as above) for activity in the oxidative deamination reaction. For the majority of the substrates, only the conversion was measured (Fig. 4). Compounds in group I were chosen to determine if the active site can accommodate larger groups than R = Me, as this is traditionally a limitation of wild-type transaminases. Notably, both transaminases successfully processed a range of substrates bearing one methyl substituent (Group II), while neither accepted substrates bearing two bulky substituents (Group I) to any great degree. Cyclic substrates (aminoindane and amino tetralins, Group III) were also chosen as these motifs are present in a wide range of pharmaceutically active compounds.

Overall the reactivity profile of the novel transaminase with these substrates was remarkably similar to that of *Cv*-ω-TA. Interestingly, the use of (±)-1-aminotetralin **14** resulted in poor conversion but the use of enantiopure (S)-1-aminotetralin (S)-**14** showed a higher degree of conversion in each case. In the majority of these cases *Cv*-ω-TA outperformed the novel *P*-ω-TA biocatalyst (Fig. 4).

### P-ω-TA exhibits enantioselectivity for a remote stereocentre

On the basis that *P*-ω-TA represented a novel class I fold transaminase, with a natural structural variation relative to available and previously characterised transaminases, we investigated *P*-ω-TA in terms of its potential for remote stereoselectivity using the amine substrates **8a** and **8b**. As our primary objective was the remote stereoselection, for clarity we investigated the thermodynamically favoured oxidative deamination reaction using the *cis*- and *trans-*diastereomers (**8b** and **8a**, respectively) separately.

**Figure 5:**
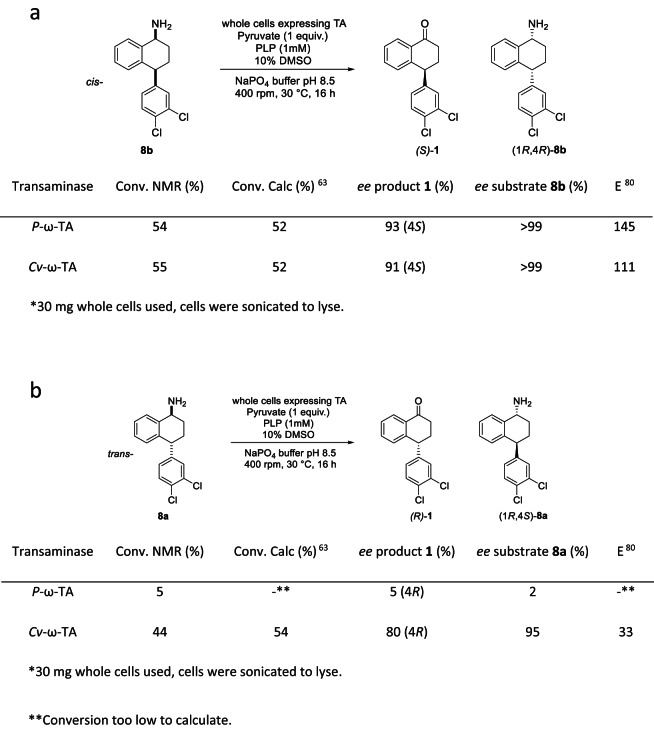
Activity of *P*-ω-TA and *Cv*-ω-TA against (**a**) *cis*-amine **8b** and (**b**) *trans*-amine **8a**. Data is from a representative experiment of three independent biological replicates with excellent repeatability.

Gratifyingly, *P*-ω-TA showed activity and enantioselectivity in experiments against the *cis*-substrate **8b**. In order to further investigate the novelty of this transformation and to gauge if the activity was indeed unusual, we examined the viability of the well-studied *Cv*-ω-TA in the same reactions. As previously stated, *Cv*-ω-TA is in the same fold class as *P*-ω-TA and hence is also (*S*)-selective^51^. In these experiments, the same conditions were used. *Cv*-ω-TA was found to be active against both **8b** and **8a** (Fig. 5). Due to the (*S*)-selectivity at the reacting site, the *trans*-stereoisomer **8a** gave rise to the (4 *R*)-tetralone, thereby showing that this enzyme is not stereo-sensitive to the remote site. Importantly, the novel transaminase only showed significant activity against **8b**, giving the (4 *S*)-tetralone product (*4 S*)-**6** in 92% *ee*, while *Cv*-ω-TA processed both the *cis-* and *trans-*amines with no evidence of discrimination at the remote stereocentre (Fig. 5). When both transaminases were used as biocatalyst, kinetic resolution of *cis*-**8b** proceeded with high efficiency within 16 h leading to essentially 50% conversion with highly enantioenriched substrates and products. Contrastingly, exposing the novel transaminase to *trans*-**8a** under the same reaction conditions resulted in poor conversion (Fig. 5). Both transaminases exhibited (*S*)-selectivity at the site of reaction, as anticipated. The selectivity of the enzyme was confirmed by comparing the reaction HPLC trace with that of a sample of the (4 *S*)-tetralone, (4 *S*)-**1** (Supplementary data). Interestingly, the transaminases showed greater activity towards the amine **8b** than the unsubstituted 1-aminotetralin **14**. Less than 10% of 1-Aminotetralin **14** was converted to product in comparison to *cis*-**8b** (up to 55% conversion, details in Fig. 5).

**Figure 6:**
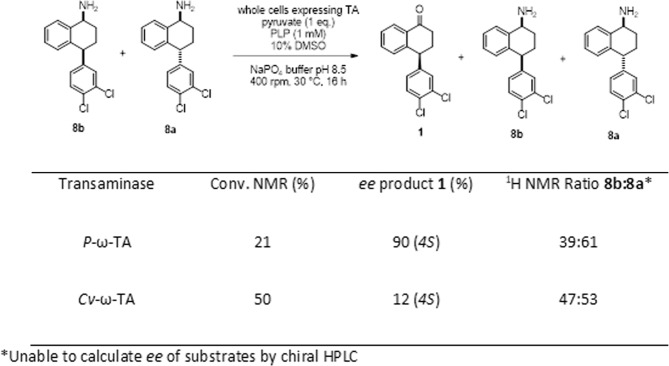
Activity of *P*-ω-TA and *Cv*-ω-TA against a mix of **8a** and **8b**. Data is from a representative experiment of two independent biological replicates with excellent repeatability.

Building on these exciting results using the individual diastereomers, an equimolar mixture of the *cis-*amine **8b** and *trans*-amine **8b** was used as substrate in the presence of both transaminases (Fig. 6). *P*-ω-TA displayed excellent discrimination at the remote stereocentre, leading to tetralone **1** with 21% conversion (90% *ee*, *4S*) – close to the kinetic limit of 25%. The lack of discrimination of *Cv*-ω-TA at the remote stereocentre was similarly confirmed as both diastereomers were processed, resulting in 50% conversion to tetralone **1**, in 12% *ee*. Thus, *P*-ω-TA exhibits excellent diastereoselection at the remote stereocentre even when using a mixture of *cis-* and *trans*-diastereomers, highlighting the synthetic potential of this novel biocatalyst.

The fact that transaminase *P*-ω-TA is (S)-selective at the 4-position as well, means that the sertraline API could be accessed via two different methods. The reductive amination reaction could successfully resolve tetralone **1**, the key intermediate in the synthesis of sertraline. After separation of the product, oxidative deamination using the same biocatalyst will furnish the enantiopure tetralone. Reductive amination with methylamine furnishes the API^45^. Alternatively, it is possible to synthesise the API directly from the (*1S*)-primary amine desmethylsertraline **8b**^46,47^.

### Modelling of the chemical transformation of 8a and 8b by *P-ω*-TA

**Figure 7:**
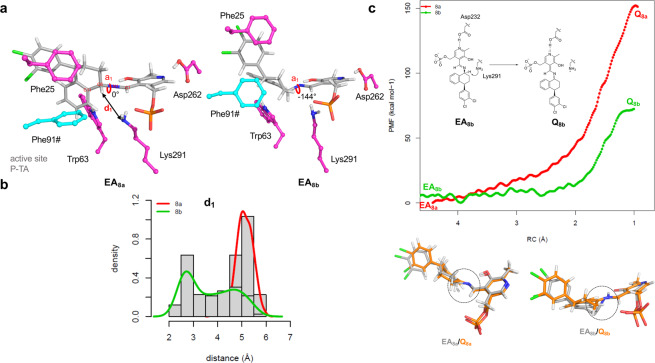
(**a**) External aldimines **EA**_**8a**_ and **EA**_**8b**_ at the active site of *P*-ω-TA. (**b)** Distribution of the distance d_1_ along the MD simulations of the external aldimines of **EA**_**8a**_ (red line) and **EA**_**8b**_ (green line) at the active site of *P*-ω-TA. (**c**) PMF (kcal mol^-1^) for the abstraction of H1 of **EA**_**8a**_ and **EA**_**8b**_ by Lys291 of *P*-ω-TA. On the bottom it is shown the structural superimposition of final **Q**_**8a**_ and **Q**_**8b**_ with the initial external aldimines. The π-conjugated system of the aldimine is highlighted with a dashed circle.

In order to understand the remote enantioselectivity shown by *P*-ω-TA in the transamination reaction towards **8b** (Fig. 5), we modelled the external aldimines **EA**_**8a**_ and **EA**_**8b**_ at the active site of *P*-ω-TA (Fig. 7). The **EA**:*P*-ω-TA complexes were built by homology modelling and manual docking of the ligand at the active site of the enzyme. Further refinement was achieved by means of a restrained Molecular Dynamics (MD) simulation protocol (see Computational methods).

In the proposed mechanism for the deamination reaction in ω-transaminases^54^, once the substrate condenses with the PLP to form the external aldimine **EA**, the general base of the reaction (neutral Lys291) abstracts the proton H1 of **8a**/**8b** to form the quinonoid intermediate **Q**, where the generated negative charge after proton abstraction is conjugated with the aldimine system and the pyridine ring. The deprotonation of the **EA** by Lys291 occurs by the *re*-face of the aldimine and it is the first key step for the stereospecificity of the reaction. Therefore, we focused on this intermediate in our MD simulation studies. Amongst others, we measured the (i) the distance between the general base of the reaction (NZ of Lys291) and the proton H1 (named d_1_, Fig. 7a) and (ii) the dihedral angle defined by the bonds C11-C6 and the N1 = C1 aldimine unit (a_1_). The latter dihedral gives a measurement of the planarity of the former conjugated system. If these two bonds are coplanar, the dihedral would have a value equal to zero. We found that the distance between H1 of **8a** and the NZ of Lys291 is larger than the distance of the former residue with the H1 of **8b** (Fig. 7b). In addition, whereas the mean value of a_1_ in **EA**_**8a**_ is 0 °, the value for a_1_ of the external aldimine **EA**_**8b**_ is −144°. That is, only **EA**_**8b**_ adopts a proficient conformation for the proton abstraction and formation of the quinonoid intermediate **Q**_**8b**_ in the active site of *P*-ω-TA. The visual inspection of the model shows that the chlorinated ring in **EA**_**8a**_ and **EA**_**8b**_ forms a ππ-stacking interaction with the Phe25 located in the N-terminal helical domain (Fig. 7a). In such a disposition, the external aldimine is constrained at the active site of *P*-ω-TA. The side chains of Phe91# on one face, and of Leu62, Trp63, and Ala234 on the other face, prevent by steric impediment the rotation around the bond N1-C6 in **EA**_**8a**_ and **EA**_**8b**_. Therefore, the remote stereogenic centre on the tetralone determines the orientation of the substrate at the binding site due to steric effects.

Guided by this finding, we simulated the proton abstraction reaction by Lys291 using quantum mechanics/molecular mechanics (QM/MM) MD simulations (Fig. 7c). We defined the shortening of the distance between the N7 of Lys291 and the proton H1 on **EA**_**8a**_ and **EA**_**8b**_ as reaction coordinate (RC). In Fig. 7c, the mean force potential (PMF, kcal mol^−1^) for this reaction towards both substrates is shown. The abstraction of H1 in **EA**_**8a**_ costs almost double the energy of that required in **EA**_**8b**_. Comparing the geometry of the obtained high-energy intermediates **Q**_**8a**_ and **Q**_**8b**_ (carbon atoms coloured in orange, bottom of Fig. 7c) with the parental external aldimines **EA**_**8a**_ and **EA**_**8b**_, respectively, it can be seen in **Q**_**8b**_ that the system has relaxed to accommodate the extra negative charge, whereas in **Q**_**8a**_ the conjugated system described above is not completely coplanar. Noteworthy, in our computations, the intermediate **Q** was obtained as a high energy state, which could indicate that a conformational change in the protein may follow to relax **Q**.

**Figure 8:**
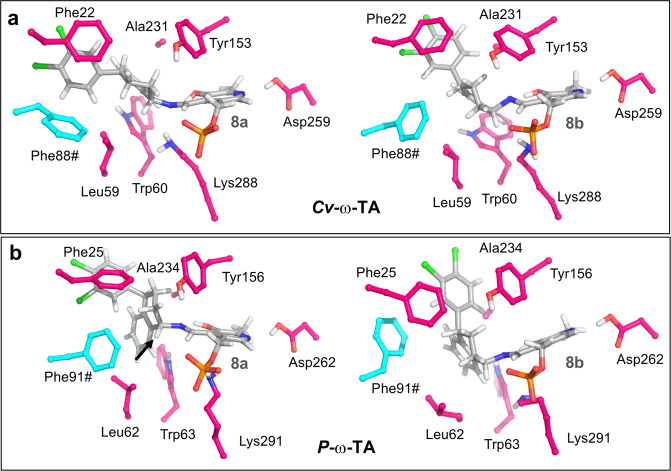
Representative structures of **8a** and **8b** at the active site of *Cv*-ω-TA (**a**) and *P*-ω-TA (**b**) obtained by means of MD simulations. The arrow highlights the different conformation of **8a** at the active site of *P*-ω-TA.

Regarding the differences of stereoselectivity shown between *P*-ω-TA and *Cv*-ω-TA in Fig. 5, we also simulated **8a** and **8b** at the active site of *Cv*-ω-TA by means of MD simulations (Fig. 8). Whereas in *P*-ω-TA the two substrates adopt different conformations as described above (highlighted by an arrow, Fig. 8b), in *Cv*-ω-TA both populate the pre-reactive conformation for the proton abstraction reaction by Lys288 (Fig. 8a). These differences can be ascribed to the changes in the sequence of the regions that cover the active site, like the N-terminal helical region (helices α_1_-α_2_, residues 1–35) and the C-terminal residues 300–320 (Supplementary Data Fig. S4), rather than changes at the active site. However, the subtle changes in the former three regions may differentially orientate the loops around the active site (e.g. α_1_-α_2,_ loop), thus covering the active site, and thereby, the orientation of the critical residues for the stereoselectivity. This hypothesis is supported by the experimental evidence that the N-terminal helical region and the former C-terminal segment have been shown to be quite flexible and to change their conformation upon binding of PLP and substrates^53^.

Finally, a structural alignment of our molecular model of P-ω-TA was carried out using the server DALI^55,56^. The best matches with a Z-score value ≥ 50 (11 structures) are listed in Supplementary Data Fig. S5 and a WebLogo^57,58^ of the structural alignment is shown in Supplementary Data Fig. S6. Structural identity with *P*-ω-TA ranged from 36–59% across the 11 TA’s. When compared with Cv- ω-TA, the best structural homologue shows variability in the residues shown in Fig. 8 (Ph25, Trp63, Phe91 and Ala234). Therefore, these positions, as well as those located at the N-terminal helical region (helices alpha1-alpha2, residues 1–35) and at the C-terminal (residues 300–320), are considered potential sites for future mutagenesis studies to modulate the stereoselectivity of P-ω-TA.

## Discussion

The surge in interest in biocatalysis has been fuelled in recent years by the attractive promise of access to biotransformations which are currently out of reach of chemical catalysts^59^. Apart from the alignment of biocatalysis with green chemistry directives and factors such as mild operating conditions and lower operating costs, microbial enzymes have the capacity to deliver on the enantioselective conversion of challenging drug intermediates and APIs^32^. Hydrolases have been comprehensively investigated as biocatalysts and have found application in (dynamic) kinetic resolutions of chiral amines^11,14,20^, including in the synthesis of norsertraline **8b**^19^. Monoamine oxidases have been used extensively in the deracemisation of racemic amines^12,15^. A number of biocatalysts have been employed in the reductive amination of imine precursors, often using super-stoichiometric amounts of the amine donor^18^. Recent work has identified a reductive aminase which can enantioselectively transfer primary and secondary amines to a carbonyl moiety, giving rise to a range of secondary and tertiary amine products, in some cases with 1 molar equivalent of the amine donor^10^.

Hydrolases can be used to resolve remote stereocentres^35–37^, and have been particularly important biocatalysts in the context of resolution of APIs and pharmaceutical intermediates^33,34,39,60^. In some cases, more than one stereocentre can be resolved^38^. Kroutil and co-workers tested 3 transaminases for activity against 2-methylcyclohexanone **2**, revealing a strong preference by the transaminase from *C. violaceum* (referred to here as *Cv*-ω-TA) for one enantiomer of the ketone starting material^41^. Limanto and colleagues used a cyclohexanone derivative **3** with a bulky ether substituent, again at the 2-position of the substrate^40^. The amine product is an intermediate in the synthesis of vernakalant, a drug in development for atrial fibrillation. Interestingly, the original transaminase variant used showed a preference for the wrong ketone enantiomer and three rounds of directed evolution (with *in-silico* design) were conducted to find an enzyme with the desired stereo preference. Apart from these two examples, transaminases have been used to set the stereochemistry at the reacting site (i.e. the primary amine) only.

In light of the dearth of direct or convenient syntheses of the API and cognisant of the exquisite selectivity of enzymes, we envisaged a biocatalytic resolution of the remote stereocentre of the Sertraline intermediate **1**. Essential to this is the discovery of the biocatalytic enantiodiscrimination of the remote stereocentre exhibited by *P*-ω-TA. The use of a biocatalyst to set the stereochemistry at the remote chiral centre could represent a substantial improvement on other reported syntheses, which revolve around classical resolution of the API with mandelic acid or the use of continuous chromatography to separate the enantiomers of **1**^45^. The former synthesis necessitates recycling of the unwanted stereoisomers *via* epimerisation of both the methine centre and the secondary amine separately. The methine racemisation of *rac*-**8b** requires a toluene reflux in the presence of 50 mol% KO^t^Bu^46–48^. Contrastingly, the presence of the electron-withdrawing ketone in tetralone **1** means that the methine centre can be racemised in the presence of 6 mol% NaOH at 50 °C, which is utilised in the continuous chromatography route^44^. Enantioselective resolution of this remote stereocentre appears to be a unique trait of the marine transaminase *P-*ω-TA uncovered in this study.

Owing to the limited structure of compounds which are accepted by wild type transaminases, the successful utilisation of a transaminase in a process has typically relied upon finding a mutant variant of natural enzymes with the desired substrate profile *via* directed evolution and/or (semi-) rational design. Our study highlights the untapped potential of harnessing novel biodiversity from pristine niches for existing framework proteins with previously unseen functionality. Transaminases have been used in the synthesis of pharmaceutical intermediates and APIs, notably in the synthesis of Januvia® (sitagliptin phosphate)^61^. In the case of sitagliptin, an (*R*)-selective fold type IV transaminase was used and 11 rounds of directed evolution, resulting in 27 mutations (8% of the entire sequence), were needed to reach the optimal protein with all of the required properties. Recently, another study described how bulky substrate acceptance could be engineered with just 4 mutations in a Fold I class transaminase^49^. More recently, dual substrate recognition for biogenic diamines and readily available monoamines has enabled researchers to adopt a *Pseudomonas putida* putrescine transaminase for the synthesis of benzylamine derivatives with excellent product conversions and extremely broad substrate tolerance^62^. Despite these and other successes, the search for enzymes with novel functionality and/or broad substrate scope is paramount to the continued development of the field, not least because these new enzymes can act as templates for further evolution of the biocatalyst. The discovery of *P*-ω-TA, a novel transaminase from the marine sponge bacterium *Pseudovibrio* WM33, offers proof of concept that natural enzymes with unique and valuable properties exist and are accessible through molecular technologies. The ability of *P*-ω-TA to accept bulky substrates, while exhibiting remote stereoselectivity, marks this enzyme as a ‘first in class’ with significant potential for synthetic optimisation.

The complexity of drug synthesis pipelines is such that single enzyme mediated resolution is limited to specific parts of the pipeline. While some success has been achieved in combining several enzymatic steps within synthesis pipelines, one-pot cascade systems remain an attractive target for biocatalytic development^63^. The Turner lab have developed cascade systems for the production of chiral 2,5-disubstituted pyrrolidines^64^, deracemization of chiral benzylic amines^65^, and production of mono- and disubstituted piperidines and pyrrolidines^66^. Cascade combination with acyl transferases^67^, and transketolases^68,69^, has also been reported. As the complexity of the cascades increases, new challenges arise. These include the propensity for side reactions and maintaining the naturally biodiverse expression systems (either homologous or heterologous) used in these one-pot cascade systems^70^. A greater understanding of the interactome dynamics between industrially relevant expression systems will provide a platform for the development of integrated and efficient cascade based systems, within which enzymes with attractive properties such as *P*-ω-TA could operate.

The unique activity of the *P*-ω-TA marine enzyme also underpins its potential as an advanced framework for directed evolutionary studies. Studies using the *Cv*-ω-TA have identified several residues in the protein framework whose substitution leads to improved properties relative to the wild-type protein^71^. A Y168F substitution was shown to increase the reaction rate 2-fold and *P-*ω-TA contains a natural Y168A substitution at this position relative to *Cv-*ω-TA. The *Cv-*ω-TA residue in all other active site substitutions in this study (A231 S, S156A, R416K, and W60C) was conserved in *P-*ω-TA. In another study, use of the proline substitution method identified several positions whose substitution resulted in increased stability and half-life^72^. The K167P substitution was found to confer enhanced melting point properties to the enzyme and *P-*ω-TA contains a natural K167S substitution relative to *Cv-*ω-TA. Dourado and colleagues described the successful directed evolution of variant (S)-selective-transaminases for asymmetric synthesis of (1S)-1-(1,1′-biphenyl-2-yl)ethanamine, achieving 1716-fold increased activity when compared with the wild-type enzyme^73^. Indeed, the explosion in available sequence data has led some researchers to develop new biocatalysts using ancestral sequence reconstruction^74^, and the growth of sequence-based databases combined with enhanced data analytics is sure to open new possibilities in TA design. Liquid and solid-phase screening technologies have emerged in recent years enabling greater access to evolved TA protein variants. These screening platforms enable access to natural biodiversity^75^, as well as evolved variants^76^. However, notwithstanding these advances, the industrial application of these novel TAs remains limited by factors such as disfavoured reaction equilibrium, poor substrate scope, and product inhibition^77^. Future studies will focus on continuous improvement of the biocatalytic activity and stability of *P-*ω-TA. This will include efforts to engineer activity in the reductive amination direction. The process of evolving optimised biocatalytic properties will be aided by combinations of high throughput technologies both for modification and detection^78^, as well as by powerful computer driven rational design^79^.

## Conclusion

A ω-transaminase from *Pseudovibrio* WM33 was identified and sequenced, showing a high degree of sequence novelty when compared against publicly available databases. This sequence novelty suggested a possible novel enzymatic function, having been isolated from an ecosystem known to contain unique chemical profiles when compared with the terrestrial environment. Further characterisation and substrate profiling demonstrated the first example of remote stereoselectivity using a transaminase biocatalyst. The unique selectivity of this enzyme isolated from a marine sponge enables the resolution of amine **8b**, a potential intermediate in the synthesis of the important antidepressant, sertraline. This resolution could fit into a synthetic route of the API. Biochemical analysis demonstrated the robustness of the enzyme at pH 8–11 and at temperatures up to 50 °C. The unique remote stereoselectivity of this enzyme, aligned with its robust biochemical profiles, makes it an excellent platform for future rational design and directed evolution studies. Furthermore, the success of the biodiscovery approach outlined in this study will serve to underpin continued investigation into biodiversity from marine niches, to harness the full potential of this ecosystem.

## Methods

### *In silico* capture of putative aminotransferase genes in the Pseudovibrio sp. WM33 genome

The genome of *Pseudovibrio* sp. WM33 (GenBank NID: LMCK00000000.1), isolated from the marine sponge *Axinella dissimilis* off the west coast of Ireland, was obtained from the NCBI database. Sequences of aminotransferase genes were analysed using Clustal Omega^81^. Enzymes encoded by these genes were subjected to preliminary domain identification. Candidate gene *P*-ω-TA was identified by annotation as a putative ω-amino acid–pyruvate aminotransferase. The putative aminotransferase gene was consistent with the structural characteristics of an ω-aminotransferase from *C. violaceum*. The NCBI Conservation Domain Database (http://www.ncbi.nlm.nih.gov/cdd/) was employed to analyse the amino acid sequence of the *P*-ω-TA conserved domain. Specific sites were identified with the aspartate aminotransferase (AAT) superfamily (fold type I) of pyridoxal phosphate (PLP)-dependent enzymes.

Alignment of protein sequences was performed by input of FASTA sequences into the T-Coffee programme (http://tcoffee.crg.cat/apps/tcoffee/do:regular)^82^. The fasta.aln file from that output was transferred to BoxShade (http://www.ch.embnet.org/software/BOX_form.html) selecting “other” as input sequence and “RTF_New” as output. Phylogenetic reconstruction of the 100 top BLASTP ‘hits’ was performed using the MEGA X programme^83^. The evolutionary history was inferred using the Maximum Likelihood method (JTT matrix-based model).

### Heterologous expression and protein purification

The predicted transaminase from *Pseudovibrio* sp WM33 was amplified using oligonucleotides purchased from Eurofins Genomics (Germany) for subcloning of the genes into a pET28a(+). The primers used were BamPTAF 5′ GAAGGATCCATGGACTATATCGCTAATTCTTCCG 3′ and XhoPTAR 5′CGTCCTCGAGTTATTTGATGCTTTGGGCAGT 3′. Similarly, the transaminase from *C. violaceum* was amplified using the primers BamCTAF 5′ GCGGGATCCATGCAGAAGCAACGTACGACCA 3′ and XhoCTAR 5′ CTATCTCGAGACTAAGCCAGCCCGCGCGCCTTC 3′. Restriction sites *BamHI* and *XhoI* were incorporated when designing the primers, respectively. The PCR conditions were as follows heated lid at 111 °C, initial denaturation for 3 min at 95 °C, then 30 cycles of: denaturation at 95 °C for 15 sec, annealing at 51 °C for 15 sec and finally extension for 3 min at 72 °C. Q5 High-Fidelity DNA Polymerase (New England Biolabs) was used for all amplifications. After the 30 cycles were complete a final elongation for 5 min at 72 °C was done. The amplicon was subjected to restriction digest using the BamHI and XhoI enzymes at 37 °C for 16 hr and purified by column extraction using a QIAGEN PCR Purification Kit (QIAGEN). The pET28a expression plasmid was similarly digested and purified to create compatible sticky ends. Ligation of insert and plasmid was carried out with the T4 Ligase (Roche) at 16 °C overnight and the reaction was subsequently transformed into *E. coli* CH3-Blue Competent Cell (Bioline). Positive clones carrying the recombinant insert were selected by PCR and conjugation was performed to introduce the construct into *E. coli* BL21(DE) RIPL for heterologous protein expression.

*E. coli* BL21(DE) RIPL cells carrying a pET28a(+) plasmid with the *P*-ω-TA and *Cv*-ω-TA were grown at 37 °C for 4 hr at which time 0.5 mM of IPTG was added to induce protein expression. The temperature was reduced down to 23 °C and induction was allowed to proceed for a further 4 hr. The pellet was collected by centrifuging the culture at 12,000 rpm at 4 °C for 10 min. The cells were lysed using CelLytic™ B Cell Lysis Reagent (Sigma) 5 ml/g of pellet, lysozyme 0.2 mg/ml, Benzonase 50 unit/ml and protease inhibitor cocktail 10 µL/ml. The lysing cells were agitated gently on a shaker at room temperature for 15 min before centrifugation at 4 °C and 12,000 rpm for 10 min.

The enzymes were purified using Poly-Prep® Chromatography Columns (Bio-Rad) and 1 ml of High Density Nickel Affinity Gel. The proteins were subsequently purified by different concentrations of Imidazole 50, 100 and 500 mM in a solution of 0.5 M NaCl and 20 mM Tris-HCl. Alternatively, proteins were also purified using the PrepEase Histidine-Tagged Protein Purification Kit (USB Corporation, USA).

### Biochemical enzyme characterisation

Enzyme activity assays were carried out at 25 °C with 2.5 mM *(S)-*α-Methylbenzylamine or 2.5 mM *(R)-*α-Methylbenzylamine (both in 50 mM phosphate buffered to pH 10), 2.5 mM pyruvate, 0.25% DMSO and 0.1 mM PLP. The activity was confirmed by the production of acetophenone which was measured at Abs_245nm_^84^. The purified enzymes were stored at −80 °C until used. The reaction was carried out at different temperatures: 20–60 °C.

The enzymes were also assessed for pH activity in order to establish the optimal conditions for enzyme activity. A pH range of 3 to 12 was assessed using sodium citrate-acetic acid and sodium phosphate-NaOH buffers.

### General procedure for the oxidative deamination of amines

From the induced transaminase enzymes in *E. coli*, 30 mg were each suspended in 50 mM sodium phosphate buffer (pH 8.5). The suspension was sonicated at 30% intensity for 10 sec, followed by 30 sec on ice. This process was repeated 5 times to lyse the cells. PLP solution (in 50 μl of buffer, final concentration of 1 mM^26,49,85,86^) and sodium pyruvate (in 50 μl of buffer, overall 1 equiv.) were added to the reaction, followed by 20 mM amine substrate in 100 μl DMSO, taking the total volume to 1 ml. The solution was shaken at 400 rpm at 30 °C for 16 hr. The reaction was stopped through the addition of 400 μl 5 M aqueous NaOH solution. To this 4 ml of ethyl acetate was added and the tubes were centrifuged to pellet the cells. The organic phase was passed through a silica plug containing Na_2_SO_4_ and the solvent was removed *in vacuo*. The crude products were analysed by ^1^H NMR and chiral HPLC (detailed methodology is provided in Supplementary Data).

### Computational methods

For the MD simulations the 3D structure of *P*-ω-TA (UniProtKB id. A0A165YA85) was generated by homology modelling using the server Phyre2.0 in the intensive mode^87^. As templates, the chain A of the crystal structure of the class III AT from *Silicibacter pomeroyi* (PDB id. 3HMU) and the chain A of the crystal structure of the ω-TA from *C. violaceum* in complex with PLP (PDB id. 4A6T) was used to model the enzyme with high confidences (>90%)^53^. The external aldimines (EA) of the substrates **8a** and **8b** (named **EA**_**8a**_ and **EA**_**8b**_, respectively) were docked manually into the active site of *P*-ω-TA and the active site of *Cv*-ω-TA after molecular superimposition with the PLP molecule in the latter template used for the modelling of the proteins. Care was taken to protonate properly the titratable residues at the active site. As an example, Asp259, which is hydrogen-bonded with the pyridine of PLP, has to be protonated in its side chain (as ASH) and His154 protonated in his delta nitrogen (as HID). In order to get the force-field parameters for the **EA**_**8a**_ and **EA**_**8b**,_ their ground state geometries were first optimised in gas phase and their electrostatic potential were computed at the standard level of theory (HF/6–31 G**//HF/3–21 G) and fitted to the atoms as RESP charges using the program antechamber (AmberTools18, http://ambermd.org). AMBER atom types were used to describe **EA**_**8a**_ and **EA**_**8b**_, and the phosphate groups were considered to be deprotonated (total charge −2). The leaprcff14SB force filed was used in all the MD simulations. The MD simulations were run on GPUs using the pmemd.cuda module of Amber16 in the Single-Precision-Fixed-Precision (SPFP) mode. The EA:enzyme complex was simulated as a protein dimer. **EA**_**8a**_ and **EA**_**8b**_ were placed in each of the active sites and the total complex was embedded in a truncated octahedral box of ca. 30400 TIP3P water molecules that extended 12 Å away from any solute atom and 32 Na^+^ ions were added to ensure charge neutrality^88^. The system was relaxed by energy minimisation in three consecutive steps (3 × 5000 cycles), in which after the first 1000 cycles the minimisation method was switched from steepest descendent to conjugate graduate. The resulting system was heated from 100 to 300 K during 200 ps with a time step of 0.2 fs and with the position of all the solute atoms restrained with a harmonic constant of 20 kcal mol^−1^ Å^−2^. The Langevin thermostat (collision frequency of 1.0 ps^−1^) was employed for the temperature regulation and the simulation was run with fixed volume (NVT ensemble)^89^. The harmonic restraints were gradually reduced in four steps from 40 to 10 kcal mol^−1^ Å^−2^. Then, the density of the system was equilibrated for 20 ps using a time step of 0.2 fs by fixing the pressure, using the Langevin thermostat with isotropic pressure scaling (NPT ensemble), and allowing the volume of the box to change. The system was simulated for 30 ns at 300 K with a time step of 2 fs without any restraint. The cut-off distance for the nonbonded interactions was 10 Å and periodic boundary conditions were used. Electrostatic interactions were treated by using the smooth particle mesh Ewald (PME) method with a grid spacing of 1 Å^90^. The SHAKE algorithm was applied to all bonds involving hydrogen atoms.

#### QM/MM MD simulations and umbrella sampling

After the MD simulation, the final snapshots were minimised with a QM/MM MD protocol to obtain a representative model of the complex **8a**:TA/**8b**:TA. The external aldimine and the side chains of Lys291 (general base of the reaction) and Asp287 were included into the QM region (77 atoms + 2 link atoms). The AA side chains included into the QM region were cut from the ß-carbon atoms. The density functional tight-binding 3 (DFTB3) semiempirical method was used to treat the QM region and the rest of the system was treated classically as described above^91^. The effect of the environment into the QM region was included using the electrostatic embedding scheme. The system was minimised for 500 cycles where the last 300 cycles were run using conjugate gradient. The reaction for the proton abstraction of the external aldimines **EA**_**8a**_ and **EA**_**8b**_ by Lys291 in *P*-ω-TA was simulated by steered QM/MM MD simulations where the reaction coordinate (RC) was defined as the shortening of the distance between the NZ of the neutral Lys291 and the H1 on the external aldimine of the *nor*-sertraline (spring constant 200 kcal mol^−1^ Å^−2^). After that, the PMF energy profile was computed by QM/MM umbrella sampling MD simulation from the geometries obtained on the previous sMD simulations (20 geometries). Each of these points (from RC = 4.7 to RC = 1.0 Å) was allowed to oscillate in a window of 2 ps. The free energy profile was calculated by the variational free energy profile (vFEP) method^92^.

### Substrate synthesis

Racemic tetralone intermediate **1** was synthesised from 1-napthol and 1,2-dichlorobenzene in the presence of a strong Lewis acid. Subsequent reduction furnished a pair of diastereomeric alcohols. These diastereomers were separated *via* flash chromatography. Mitsunobu chemistry provided the *trans*- and *cis*-azides with inversion of stereochemistry, which were converted to the target amines **8a** and **8b** *via* a Staudinger reduction **(**Supplementary data).

## Supplementary information

Supplementary Information.

## Data Availability

All data generated or analysed during this study are included in this published article (and its supplementary information files).

## References

[1] Choi JM, Han SS, Kim HS (2015). Industrial applications of enzyme biocatalysis: Current status and future aspects. Biotechnol Adv.

[2] Reetz MT (2013). Biocatalysis in Organic Chemistry and Biotechnology: Past, Present, and Future. J Am Chem Soc.

[3] Sun HH, Zhang HF, Ang EL, Zhao HM (2018). Biocatalysis for the synthesis of pharmaceuticals and pharmaceutical intermediates. Bioorgan Med Chem.

[4] Torrelo G, Hanefeld U, Hollmann F (2015). Biocatalysis. Catal Lett.

[5] de Souza ROMA, Miranda LSM, Bornscheuer UT (2017). A Retrosynthesis Approach for Biocatalysis in Organic Synthesis. Chem-Eur J.

[6] Turner NJ, O’Reilly E (2013). Biocatalytic retrosynthesis. Nat Chem Biol.

[7] Nugent, T. C. *Chiral Amine Synthesis: Methods, Developments and Application* (WILEY-VCH Verlag GmbH & Co. KGaA, 2010).

[8] Kellogg, R. M. & Leeman, M. In *Comprehensive Chirality* Vol. 9 (eds. Yamamoto, H. & Carreira, E.) 367–399 (Elsevier Inc., 2012).

[9] Kiwala D (2016). Separation of Stereoisomeric Mixtures of Nafronyl as a Representative of Compounds Possessing Two Stereogenic Centers By Coupling Crystallization, Diastereoisomeric Conversion and Chromatography. Org Process Res Dev.

[10] Aleku GA (2017). A reductive aminase from *Aspergillus oryzae*. Nat Chem.

[11] Blacker AJ, Stirling MJ, Page MI (2007). Catalytic racemisation of chiral amines and application in dynamic kinetic resolution. Org Process Res Dev.

[12] Ghislieri D (2013). Engineering an Enantioselective Amine Oxidase for the Synthesis of Pharmaceutical Building Blocks and Alkaloid Natural Products. J Am Chem Soc.

[13] Ghislieri D, Turner NJ (2014). Biocatalytic Approaches to the Synthesis of Enantiomerically Pure Chiral Amines. Top Catal.

[14] Gotor-Fernandez V, Brieva R, Gotor V (2006). Lipases: Useful biocatalysts for the preparation of pharmaceuticals. J Mol Catal B-Enzym.

[15] Herter S (2018). Mapping the substrate scope of monoamine oxidase (MAO-N) as a synthetic tool for the enantioselective synthesis of chiral amines. Bioorgan Med Chem.

[16] Hohn M, Bornscheuer UT (2009). Biocatalytic Routes to Optically Active Amines. Chemcatchem.

[17] Kroutil W (2013). Asymmetric Preparation of prim-, sec-, and tert-Amines Employing Selected Biocatalysts. Org Process Res Dev.

[18] Schrittwieser JH, Velikogne S, Kroutil W (2015). Biocatalytic Imine Reduction and Reductive Amination of Ketones. Adv Synth Catal.

[19] Thalen LK (2009). A Chemoenzymatic Approach to Enantiomerically Pure Amines Using Dynamic Kinetic Resolution: Application to the Synthesis of Norsertraline. Chem-Eur J.

[20] van Rantwijk F, Sheldon RA (2004). Enantioselective acylation of chiral amines catalysed by serine hydrolases. Tetrahedron.

[21] Guo F, Berglund P (2017). Transaminase biocatalysis: optimization and application. Green Chem.

[22] Mathew S, Yun H (2012). omega-Transaminases for the Production of Optically Pure Amines and Unnatural Amino Acids. ACS Catal.

[23] Paul CE (2014). Transaminases Applied to the Synthesis of High Added-Value Enantiopure Amines. Org Process Res Dev.

[24] Simon RC, Richter N, Busto E, Kroutil W (2014). Recent Developments of Cascade Reactions Involving omega-Transaminases. ACS Catal.

[25] Slabu I, Galman JL, Lloyd RC, Turner NJ (2017). Discovery, Engineering, and Synthetic Application of Transaminase Biocatalysts. ACS Catal.

[26] Borner T (2017). Explaining Operational Instability of Amine Transaminases: Substrate-Induced Inactivation Mechanism and Influence of Quaternary Structure on Enzyme-Cofactor Intermediate Stability. ACS Catal.

[27] Hohne M, Schatzle S, Jochens H, Robins K, Bornscheuer UT (2010). Rational assignment of key motifs for function guides in silico enzyme identification. Nat Chem Biol.

[28] Feng YH (2017). Development of an Efficient and Scalable Biocatalytic Route to (3R)-3-Aminoazepane: A Pharmaceutically Important Intermediate. Org Process Res Dev.

[29] Truppo MD, Rozzell JD, Turner NJ (2010). Efficient Production of Enantiomerically Pure Chiral Amines at Concentrations of 50 g/L Using Transaminases. Org Process Res Dev.

[30] Reen FJ, Romano S, Dobson ADW, O’Gara F (2015). The Sound of Silence: Activating Silent Biosynthetic Gene Clusters in Marine Microorganisms. Mar Drugs.

[31] Parages, M. L., Gutierrez-Barranquero, J. A., Reen, F. J., Dobson, A. D. W. & O’Gara, F. Integrated (Meta) Genomic and Synthetic Biology Approaches to Develop New Biocatalysts. *Mar Drugs*, **14** (3), (2016). 10.3390/md14030062 PMC4810074 27007381

[32] Castilla, I. A., Woods, D. F., Reen, F. J. & O’Gara, F. Harnessing Marine Biocatalytic Reservoirs for Green Chemistry Applications through Metagenomic Technologies. *Mar Drugs*, **16**(7), (2018). 10.3390/md16070227 PMC6071119 29973493

[33] Barbayianni E, Kokotos G (2012). Biocatalyzed Regio- and Chemoselective Ester Cleavage: Synthesis of Bioactive Molecules. Chemcatchem.

[34] Cunha RLOR, Ferreira EA, Oliveira CS, Omori AT (2015). Biocatalysis for desymmetrization and resolution of stereocenters beyond the reactive center: How far is far enough?. Biotechnol Adv.

[35] Deasy RE, Brossat M, Moody TS, Maguire AR (2011). Lipase catalysed kinetic resolutions of 3-aryl alkanoic acids. Tetrahedron-Asymmetr.

[36] Deasy RE, Moody TS, Maguire AR (2013). Influence of the position of the substituent on the efficiency of lipase-mediated resolutions of 3-aryl alkanoic acids. Tetrahedron-Asymmetr.

[37] Foley AM, Gavin DP, Joniec I, Maguire AR (2017). Impact of variation of the acyl group on the efficiency and selectivity of the lipase-mediated resolution of 2-phenylalkanols. Tetrahedron-Asymmetr.

[38] Gavin DP (2017). Hydrolase-mediated resolution of the hemiacetal in 2-chromanols: The impact of remote substitution. Tetrahedron-Asymmetr.

[39] Mendez-Sanchez D, Lopez-Iglesias M, Gotor-Fernandez V (2016). Hydrolases in Organic Chemistry. Recent Achievements in the Synthesis of Pharmaceuticals. Curr Org Chem.

[40] Limanto J (2014). A Highly Efficient Asymmetric Synthesis of Vernakalant. Org Lett.

[41] Richter N (2015). omega-Transaminases for the amination of functionalised cyclic ketones. Org Biomol Chem.

[42] Koblan KS (2015). Dasotraline for the Treatment of Attention-Deficit/Hyperactivity Disorder: A Randomized, Double-Blind, Placebo-Controlled, Proof-of-Concept Trial in Adults. Neuropsychopharmacol.

[43] Bogeso KP, Christensen AV, Hyttel J, Liljefors T (1985). 3-Phenyl-1-Indanamines - Potential Antidepressant Activity and Potent Inhibition of Dopamine, Norepinephrine, and Serotonin Uptake. J Med Chem.

[44] Lee SH (2011). Stereoselective Amination of Chiral Benzylic Ethers Using Chlorosulfonyl Isocyanate: Total Synthesis of (+)-Sertraline. J Org Chem.

[45] Quallich GJ (2005). Development of the commercial process for Zoloft((R))/sertraline. Chirality.

[46] Chandrasekhar S, Reddy MV (2000). An expedient total synthesis of cis-(+)-Sertraline from D-phenylglycine. Tetrahedron.

[47] Fustero S (2013). Asymmetric Allylation/Ring Closing Metathesis: One-Pot Synthesis of Benzo-fused Cyclic Homoallylic Amines. Application to the Formal Synthesis of Sertraline Derivatives. Org Lett.

[48] Wang GY, Zheng CW, Zhao G (2006). Asymmetric reduction of substituted indanones and tetralones catalyzed by chiral dendrimer and its application to the synthesis of (+)-sertraline. Tetrahedron-Asymmetr.

[49] Pavlidis IV (2016). Identification of (S)-selective transaminases for the asymmetric synthesis of bulky chiral amines. Nat Chem.

[50] Cho BK (2008). Redesigning the substrate specificity of omega-aminotransferase for the kinetic resolution of aliphatic chiral arnines. Biotechnol Bioeng.

[51] Kaulmann U, Smithies K, Smith MEB, HaileS HC, Ward JM (2007). Substrate spectrum of omega-transaminase from *Chromobacterium violaceum* DSM30191 and its potential for biocatalysis. Enzyme Microb Tech.

[52] Koszelewski D, Tauber K, Faber K, Kroutil W (2010). omega-Transaminases for the synthesis of non-racemic alpha-chiral primary amines. Trends Biotechnol.

[53] Humble MS (2012). Crystal structures of the *Chromobacterium violaceum* ω-transaminase reveal major structural rearrangements upon binding of coenzyme PLP. FEBS J.

[54] van Oosterwijk N (2016). Structural Basis of the Substrate Range and Enantioselectivity of Two (S)-Selective omega-Transaminases. Biochemistry.

[55] Holm L, Laakso LM (2016). Dali server update. Nucleic Acids Res.

[56] Holm L, Sander C (1995). Dali: a network tool for protein structure comparison. Trends Biochem Sci.

[57] Crooks GE, Hon G, Chandonia JM, Brenner SE (2004). WebLogo: a sequence logo generator. Genome Res.

[58] Schneider TD, Stephens RM (1990). Sequence logos: a new way to display consensus sequences. Nucleic Acids Res.

[59] Rosenthal K, Lutz S (2018). Recent developments and challenges of biocatalytic processes in the pharmaceutical industry. Curr Opin Green Sust.

[60] Blasco MA, Groger H (2014). Enzymatic resolution of racemates with a ‘remote’ stereogenic center as an efficient tool in drug, flavor and vitamin synthesis. Bioorgan Med Chem.

[61] Savile CK (2010). Biocatalytic Asymmetric Synthesis of Chiral Amines from Ketones Applied to Sitagliptin Manufacture. Science.

[62] Galman, J. L. *et al*. Characterization of a Putrescine Transaminase From *Pseudomonas putida* and its Application to the Synthesis of Benzylamine Derivatives. *Front Bioeng Biotech*, **6**, (2018). 10.3389/fbioe.2018.00205 PMC6308316 30622946

[63] Kelly SA (2018). Application of omega-Transaminases in the Pharmaceutical Industry. Chem Rev.

[64] O’Reilly E (2014). A Regio-and Stereoselective ω-Transaminase/Monoamine Oxidase Cascade for the Synthesis of Chiral 2,5-Disubstituted Pyrrolidines. Angew Chem Int Edit.

[65] O’Reilly E, Iglesias C, Turner NJ (2014). Monoamine Oxidase- ω- Transaminase Cascade for the Deracemisation and Dealkylation of Amines. Chemcatchem.

[66] France SP (2016). One-Pot Cascade Synthesis of Mono- and Disubstituted Piperidines and Pyrrolidines using Carboxylic Acid Reductase (CAR), omega-Transaminase (omega-TA), and Imine Reductase (IRED) Biocatalysts. ACS Catal.

[67] Land H, Hendil-Forssell P, Martinelle M, Berglund P (2016). One-pot biocatalytic amine transaminase/acyl transferase cascade for aqueous formation of amides from aldehydes or ketones. Catal Sci Technol.

[68] Lorilliere M (2017). One-pot, two-step cascade synthesis of naturally rare L-erythro (3S,4S) ketoses by coupling a thermostable transaminase and transketolase. Green Chem.

[69] Bawn M (2018). One-pot, two-step transaminase and transketolase synthesis of l-gluco-heptulose from l-arabinose. Enzyme Microb Technol.

[70] Muschiol J (2015). Cascade catalysis–strategies and challenges en route to preparative synthetic biology. Chem Commun (Camb).

[71] Almahboub SA, Narancic T, Fayne D, O’Connor KE (2018). Single point mutations reveal amino acid residues important for *Chromobacterium violaceum* transaminase activity in the production of unnatural amino acids. Sci Rep.

[72] Land H, Campillo-Brocal JC, Svedendahl Humble M, Berglund P (2019). B-factor Guided Proline Substitutions in *Chromobacterium violaceum* Amine Transaminase: Evaluation of the Proline Rule as a Method for Enzyme Stabilization. Chembiochem.

[73] Dourado DFAR (2016). Rational Design of a (S)-Selective-Transaminase for Asymmetric Synthesis of (1S)-1-(1,1 ‘-biphenyl-2-yl)ethanamine. ACS Catal.

[74] Wilding M (2017). Reverse engineering: transaminase biocatalyst development using ancestral sequence reconstruction. Green Chem.

[75] Coscolin C (2019). Bioprospecting Reveals Class III omega-Transaminases Converting Bulky Ketones and Environmentally Relevant Polyamines. Appl Environ Microb.

[76] Planchestainer M, Hegarty E, Heckmann CM, Gourlay LJ, Paradisi F (2019). Widely applicable background depletion step enables transaminase evolution through solid-phase screening. Chem Sci.

[77] Patil, M. D., Grogan, G., Bommarius, A. & Yun, H. Recent Advances in omega-Transaminase-Mediated Biocatalysis for the Enantioselective Synthesis of Chiral Amines. *Catalysts*, **8**,(2018).

[78] Zeymer C, Hilvert D (2018). Directed Evolution of Protein Catalysts. Annu Rev Biochem.

[79] Bornscheuer UT, Hauer B, Jaeger KE, Schwaneberg U (2019). Directed Evolution Empowered Redesign of Natural Proteins for the Sustainable Production of Chemicals and Pharmaceuticals. Angew Chem Int Ed Engl.

[80] Chen CS, Fujimoto Y, Girdaukas G, Sih CJ (1982). Quantitative-Analyses of Biochemical Kinetic Resolutions of Enantiomers. J Am Chem Soc.

[81] Sievers F, Higgins DG (2014). Clustal Omega. Curr Protoc Bioinformatics.

[82] Di Tommaso P (2011). T-Coffee: a web server for the multiple sequence alignment of protein and RNA sequences using structural information and homology extension. Nucleic Acids Res.

[83] Kumar S, Stecher G, Li M, Knyaz C, Tamura K (2018). MEGA X: Molecular Evolutionary Genetics Analysis across Computing Platforms. Mol Biol Evol.

[84] Schatzle S, Hohne M, Redestad E, Robins K, Bornscheuer UT (2009). Rapid and Sensitive Kinetic Assay for Characterization of omega-Transaminases. Anal Chem.

[85] Koszelewski D, Pressnitz D, Clay D, Kroutil W (2009). Deracemization of mexiletine biocatalyzed by omega-transaminases. Org Lett.

[86] Shin JS, Yun H, Jang JW, Park I, Kim BG (2003). Purification, characterization, and molecular cloning of a novel amine:pyruvate transaminase from *Vibrio fluvialis* JS17. Appl Microbiol Biotechnol.

[87] Kelley LA, Mezulis S, Yates CM, Wass MN, Sternberg MJE (2015). The Phyre2 web portal for protein modeling, prediction and analysis. Nat Protoc.

[88] Jorgensen WL, Chandrasekhar J, Madura JD, Impey RW, Klein ML (1983). Comparison of Simple Potential Functions for Simulating Liquid Water. J Chem Phys.

[89] Pastor RW, Brooks BR, Szabo A (1988). An Analysis of the Accuracy of Langevin and Molecular-Dynamics Algorithms. Mol Phys.

[90] Darden T, York D, Pedersen L (1993). Particle Mesh Ewald - an N.Log(N) Method for Ewald Sums in Large Systems. J Chem Phys.

[91] Seabra deM, Walker G, Elstner RC, Case M, Roitberg DA (2007). A. E. Implementation of the SCC-DFTB method for hybrid QM/MM simulations within the amber molecular dynamics package. J Phys Chem A.

[92] Lee, T. S., Radak, B. K., Huang, M., Wong, K. Y. & York, D. M. Roadmaps through Free Energy Landscapes Calculated Using the Multidimensional vFEP Approach. *J Chem Theory Comput*, **10**(1), 24–34, (2014). 10.1021/ct400691f PMC3912246 24505217

