# Author Correction: Genome mining and characterisation of a novel transaminase with remote stereoselectivity

**DOI:** 10.1038/s41598-022-12801-5

**Published:** 2022-05-27

**Authors:** D. P. Gavin, F. J. Reen, J. Rocha-Martin, I. Abreu-Castilla, D. F. Woods, A. M. Foley, P. A. Sánchez-Murcia, M. Schwarz, P. O’Neill, A. R. Maguire, F. O’Gara

**Affiliations:** 1 School of Chemistry; Analytical and Biological Chemistry Research Facility, University College Cork, Cork, Ireland; 2 Synthesis and Solid State Pharmaceutical Centre, University College Cork, Cork, Ireland; 3 BIOMERIT Research Centre, School of Microbiology, University College Cork, Cork, Ireland; 4 School of Microbiology, University College Cork, T12 K8AF Cork, Ireland; 5 School of Chemistry, School of Pharmacy, Analytical and Biological Chemistry Research Facility, University College Cork, Cork, Ireland; 6 Institute of Theoretical Chemistry, Faculty of Chemistry, University of Vienna, Währinger Str. 17, A-1090 Vienna, Austria; 7 Pfizer Process Development Centre, Loughbeg, Cork, Ireland; 8 Human Microbiome Programme, School of Pharmacy and Biomedical Sciences, Curtin Health Innovation Research Institute, Curtin University, Perth, WA 6102, Australia and Telethon Kids Institute, Perth, WA 6008 Australia

Correction to: *Scientific Reports* https://doi.org/10.1038/s41598-019-56612-7, published online 30 December 2019

The original version of this Article contained an error in Figure 5 (a) label, where the stereochemistry label was incorrect.

“(1S, 4S)-8b”

now reads:

“(1R, 4R)-8b”

The original Figure 5 and accompanying legend appear below.

The original Article has been corrected.

**Figure 5:**
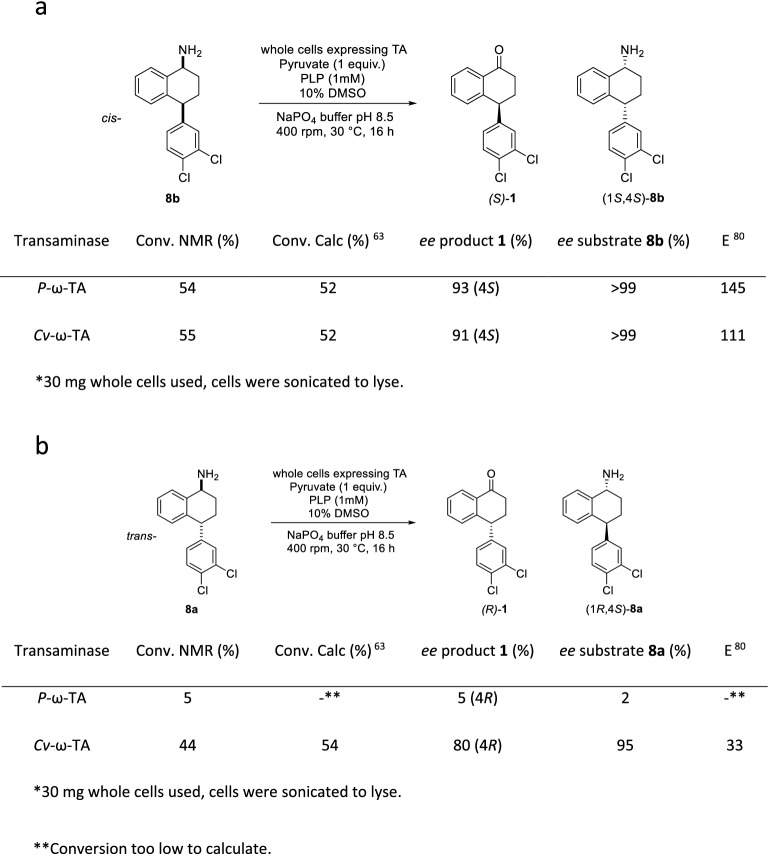
Activity of *P*-ω-TA and *Cv*-ω-TA against (**a**) *cis*-amine **8b** and (**b**) *trans*-amine **8a**. Data is from a representative experiment of three independent biological replicates with excellent repeatability.

