# Supplementary material for: Genome mining and characterisation of a novel transaminase with remote stereoselectivity
Source: Sci Rep. 2019 Dec 30;9:20285. doi: 10.1038/s41598-019-56612-7 (PMC6937235; doi:10.1038/s41598-019-56612-7)
Supplement: Supplementary file 1 — Supplementary Information. [file 41598_2019_56612_MOESM1_ESM.pdf]

Genome mining and characterisation of a novel transaminase with remote stereoselectivity

Gavin, DP[¥1,2], Reen, FJ[¥3,4], Rocha-Martin, J[¥3], Abreu-Castilla, I[3], Woods, DF[3], Foley, AM[5], Sánchez-Murcia, PA[6], Schwarz, M[1], O'Neill, P[7], Maguire, AR[2,5*] and O'Gara, F[2,3,8*]

[1]School of Chemistry; Analytical and Biological Chemistry Research Facility; University College Cork, Cork, Ireland

[2]Synthesis and Solid State Pharmaceutical Centre, University College Cork, Cork, Ireland

[3]BIOMERIT Research Centre, School of Microbiology, University College Cork, Cork, Ireland

[4]School of Microbiology, University College Cork, T12 K8AF Cork, Ireland

[5]School of Chemistry, School of Pharmacy, Analytical and Biological Chemistry Research Facility, University College Cork, Cork, Ireland

[6]Institute of Theoretical Chemistry, Faculty of Chemistry, University of Vienna, Währinger Str. 17 A-1090 Vienna, Austria

[7] Pfizer Process Development Centre, Loughbeg, Cork, Ireland.

[8]Human Microbiome Programme, School of Pharmacy and Biomedical Sciences, Curtin Health Innovation Research Institute, Curtin University, Perth, WA 6102, Australia and Telethon Kids Institute, Perth, WA 6008, Australia

[¥] Authors contributed equally

[*]Corresponding authors: a.maguire@ucc.ie; f.ogara@ucc.ie

Contents

1. Supplementary Figures S3
2. Synthesis and characterisation of compounds S5
3. HPLC chromatograms S11
4. ^{1}H & ^{13}C NMR spectra S19

1. Supplementary Figures

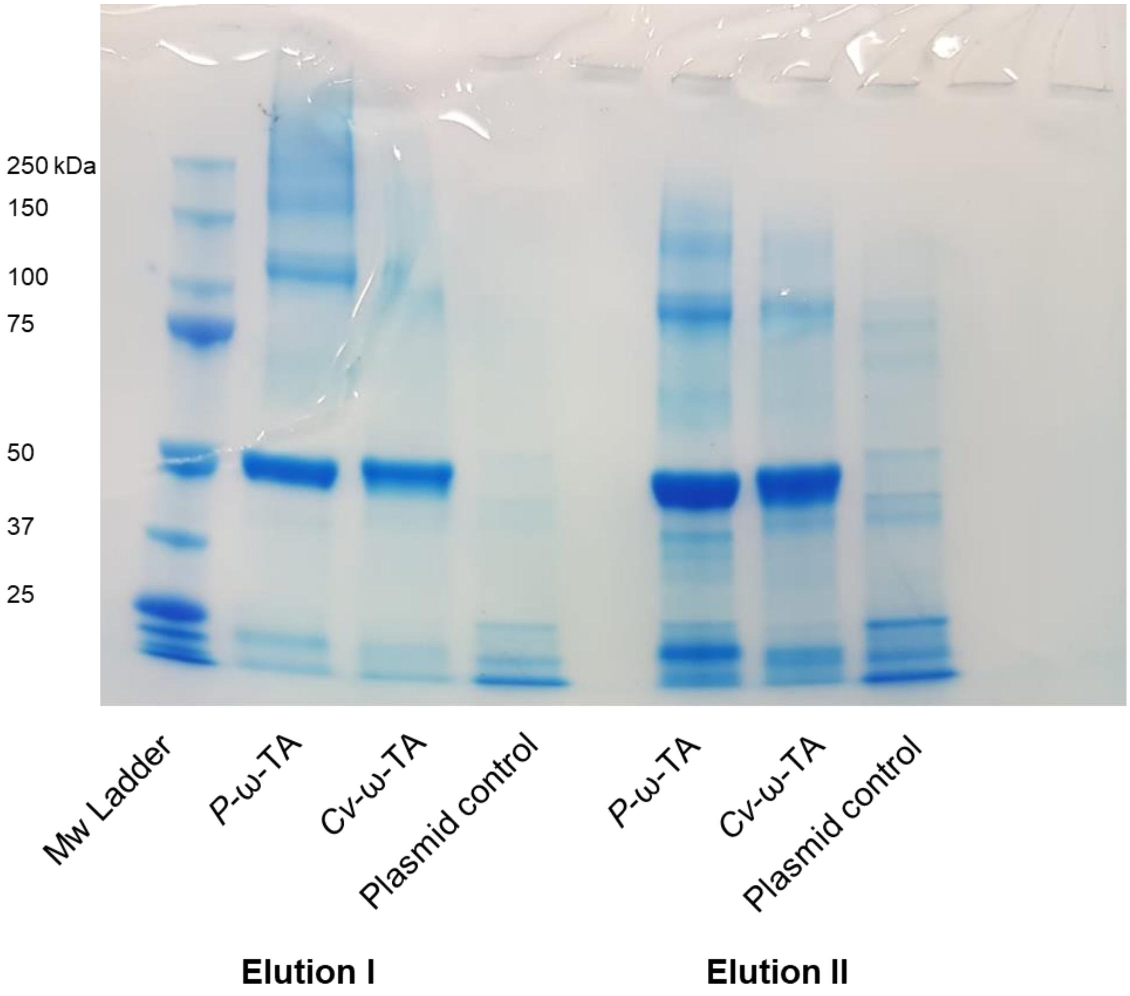

Figure S1. Purification and SDS PAGE analysis of *P*-ω-TA and *Cv*-ω-TA. Both proteins are approximately 50 kDa in size. An empty plasmid pET28a control was processed along with both recombinant constructs and is included here for validation.

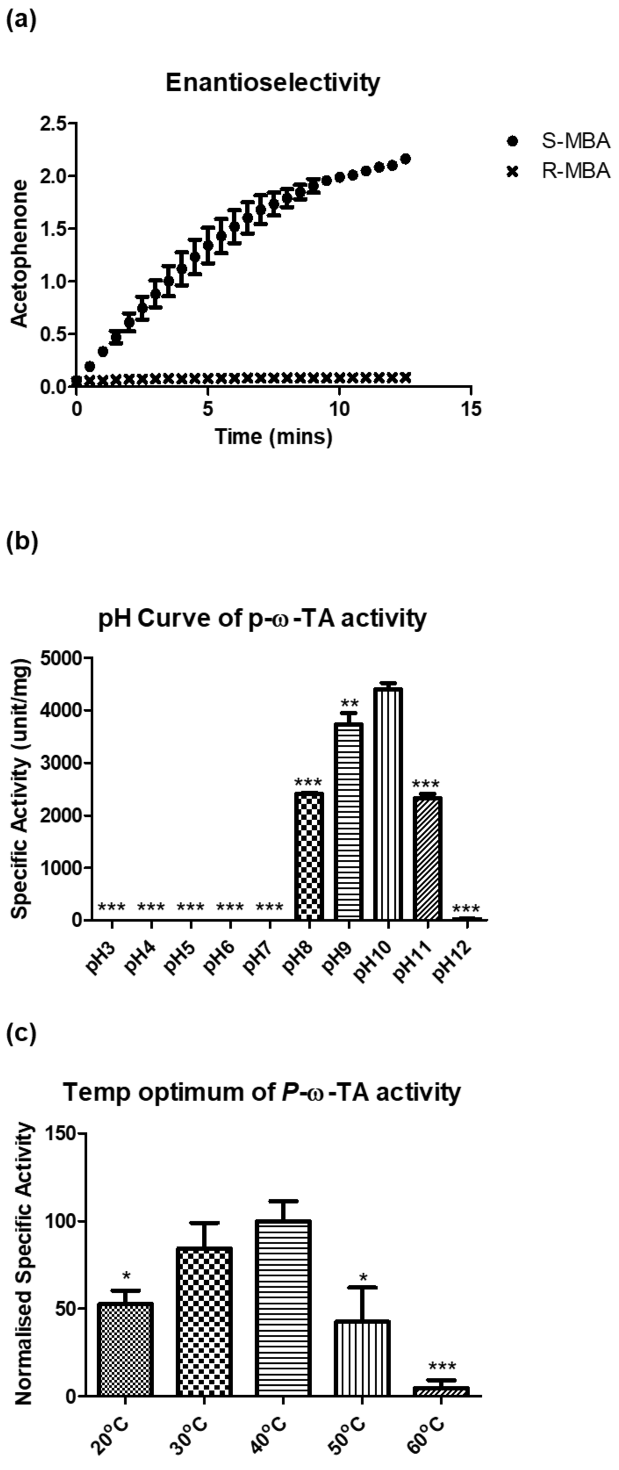

Figure S2. Biochemical characterisation of P-ω-TA representing **a** Enantioselectivity, **b** pH, and **c** Temperature optima. pH data is presented for assays performed at the optimum temperature of 40°C, while temperature optima data is presented for assays performed at the pH optimum of 10. Data is normalised to the average of the optimum value in both cases. Data presented is the average of three independent biological replicates and variation is presented as standard error of the mean. Statistical analysis was performed using One way ANOVA with Dunnett's multiple comparison corrective testing to the optimum activity (* $p < 0.05$, ** $p < 0.01$, *** $p < 0.001$).

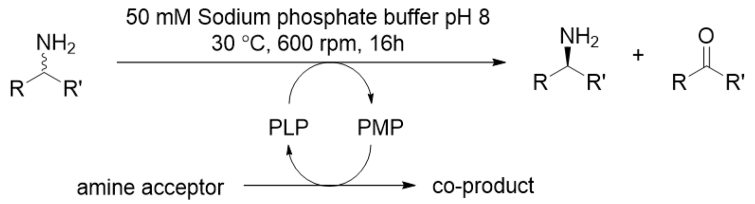

Substrate	Amine acceptor	DMSO	Conversion
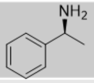	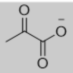	5%	71%
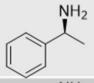	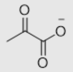	10%	100%
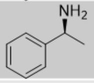	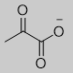	0	26%
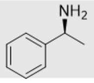	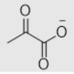	20%	25%

Figure S3. Solvent tolerance of *P*-ω-TA in the presence of a range of DMSO concentrations.

```
P-ω-TA              MDYIANLPPTHVLQEKDAAHHLHPFTDTKSLNAKGTRVITRADGVYLWDSEGNKILDGMA 60
Cv-ω-TA             ---MQKQRTTSQWRELDAAHHLHPFTDTASLNQAGARVMTRGEGVYLWDSEGNKIIDGMA 57
                    : :   *   :  *********: . :*.  *:**:**. **:* ****::*:*.**

P-ω-TA              GLWCVNVGYGRQEIIDAVYRQMQQLPYYNTFFQSSHPPAIGLAERISSLAPDHLDHVFFA 120
Cv-ω-TA             GLWCVNVGYGRKDFAEAARRQMEELPFYNTFFKTTHPAVVELSSLLAEVTPAGFDRVFYT 117
                    ******:****.:: :.. ***.:**:*****:::*  .: *:. ::.::*  :::**::

P-ω-TA              GSGSEANDTVVRMVRHYWASEGKPTKKTIISRHNAYHGSTMAGASLGGMSAMHAQGGLPI 180
Cv-ω-TA             NSGSESVDTMIRMVRRYWDVQGKPEKKTLIGRWNGYHGSTIGGASLGGMKYMHEQGDLPI 177
                    ..***: ** :**** **  :*:* *..:*.* *.*****:..::****  ** *..* *

P-ω-TA              PDITHINQPYWYGEGGDMDPAAFGLMRARELEAEIDRLGEDNVAAFIGEPIQGAGGVIIP 240
Cv-ω-TA             PGMAHIEQPWWYKHGKDMTPDEFGVVAARWLEEKILEIGADKVAAFVGEPIQGAGGVIVP 237
                    *.: **:** *: .* ** *  **:  ** **  * .:* :.****:.**:*******:

P-ω-TA              PETYWPEIQRICRERNILLIADEVICGFGRTGNWFGSQTFNFKPDLMPIAKGLSSGYLPI 300
Cv-ω-TA             PATYWPEIERICRKYDVLLVADEVICGFGRTGEWFGHQHFGFQPDLFTAAKGLSSGYLPI 297
                    * :*****:*** : ::**:************:*** * :.::*.::  ******** **

P-ω-TA              GAVVVSEKVAKGFIEHGGEFYHGFTYSAHPAACAAALANLDIIENERLPEKVANDTGPYL 360
Cv-ω-TA             GAVFVGKRVAEGLI-AGGDFNHGFTYSGHPVCAAVAHANVAALRDEGIVQRVKDDIGPYM 356
                    *. .* ..**.      .:* **:***.**...*.*  *:  :.:* : ::* :  .**:

                    AEKWKT-LGEHPLVGEARICGLVGALELSPDKARRARFEAEKGTVGTICRDHCFESGLVM 419
Cv-ω-TA             QKRWRETFSRFEHVDDVRGVGMVQAFTLVKNKAKRELF-PDFGEIGTLCRDIFFRNNLIM 415
                    ::*.  :  .  *.:.:  *:: :: *  :** *  *  : * :* :**:  * ..*:*

P-ω-TA              RHVGDSMIISPPLVISRSEVDELIQKAHRALDLTAADVTAQSIK---- 463
Cv-ω-TA             RACGDHIVSAPPLVMTRAEVDEMLAVAERCLEEFEQTLKARGLA---- 459
                    *  ** :: :****:: :*:**::   .:.*:     :  :.:
```

Figure S4. Sequence alignment of *P*-ω-TA (A0A165YA85) and *Cv*-ω-TA (Q7NWG4) with Cluster Omega. The conserved residues at the active site are shown in bold. The residues that change in the regions around the active site are highlighted in blue.

No:	Chain	Z	rmsd	lali	nres	%id	PDB	Description
1:	6gwi-B	53.0	1.3	450	450	59	PDB	MOLECULE: PUTRESCINE AMINOTRANSFERASE;
2:	4a6t-C	52.2	1.3	454	455	53	PDB	MOLECULE: OMEGA TRANSAMINASE;
3:	3hmu-A	51.5	1.4	454	464	57	PDB	MOLECULE: AMINOTRANSFERASE, CLASS III;
4:	5kr6-B	50.5	1.7	450	460	44	PDB	MOLECULE: 4-AMINOBUTYRATE TRANSAMINASE;
5:	6g4b-B	50.2	1.5	445	453	42	PDB	MOLECULE: ASPARTATE AMINOTRANSFERASE FAMILY PROTEIN;
6:	3gju-A	50.1	1.6	445	458	36	PDB	MOLECULE: PUTATIVE AMINOTRANSFERASE;
7:	5kqu-C	50.0	1.6	450	459	42	PDB	MOLECULE: 4-AMINOBUTYRATE TRANSAMINASE;
8:	6io1-B	49.7	1.8	448	448	41	PDB	MOLECULE: AMINOTRANSFERASE, CLASS III;
9:	6fyq-A	49.7	1.4	435	443	37	PDB	MOLECULE: AMINE TRANSAMINASE;
10:	5kr3-A	49.7	1.5	448	458	42	PDB	MOLECULE: 4-AMINOBUTYRATE TRANSAMINASE;
11:	5ghg-B	49.6	1.6	431	433	41	PDB	MOLECULE: AMINOTRANSFERASE CLASS-III;

Figure S5. Best 11 solutions of the structural alignment of our molecular model of *P*-ω-TA using the DALI server based on the Z-score value (≥50.0).

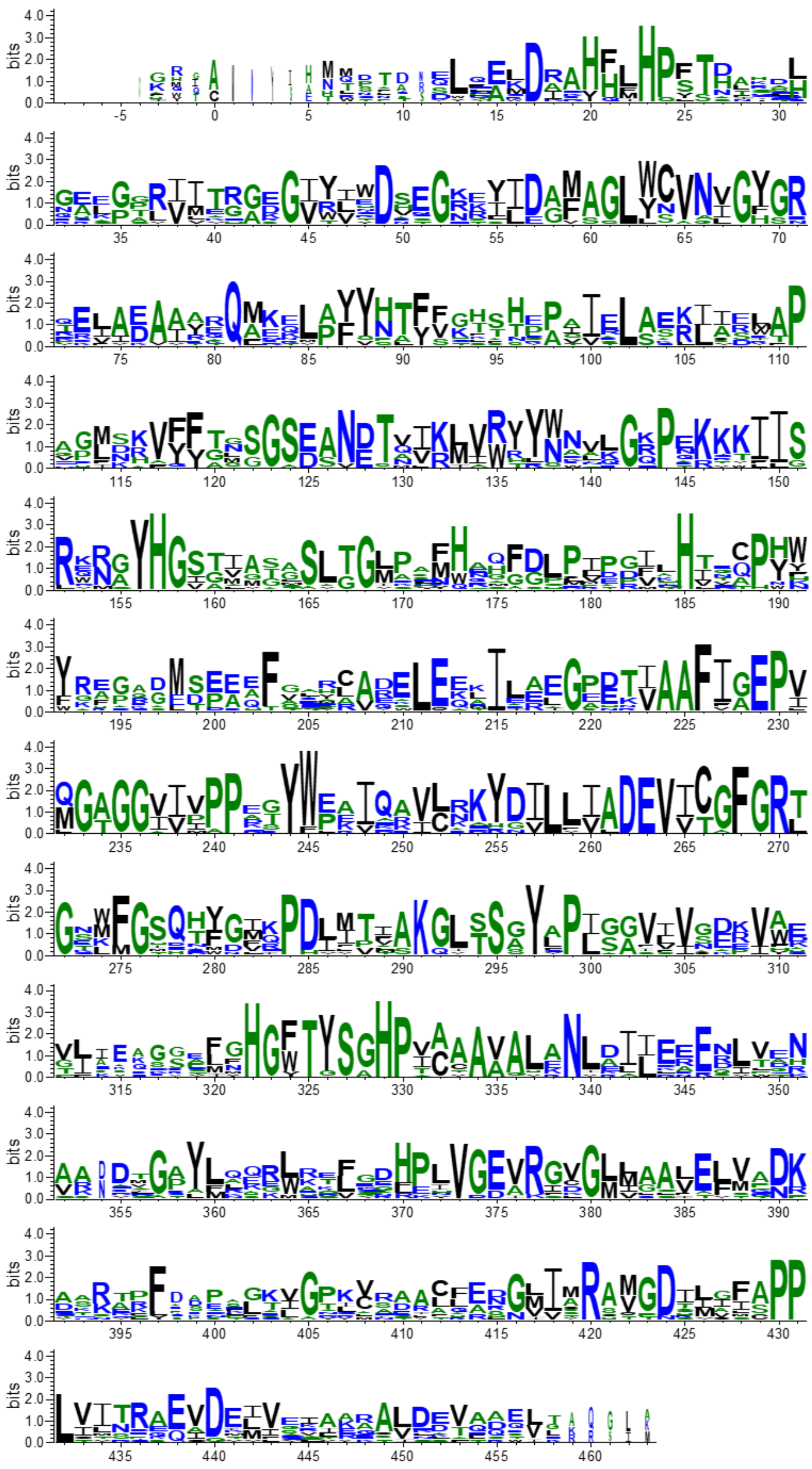

Figure S6. WebLogo of the structural alignment of the best 11 proteins shown in Figure S5. P-ω-TA is used as reference sequence.

2. Synthesis and characterisation of substrates

Synthesis of model amine compounds

General procedure for reductive amination for preparation of racemic amines [1]

A mixture of ketone (5.0 mmol), titanium(IV) ethoxide (10.0 mmol, 2 eq.) and ammonia (2 M in methanol, 25.0 mmol, 5 eq.) was stirred under nitrogen, at ambient temperature for 15 h. Sodium borohydride (7.5 mmol, 1.5 eq.) was added and the resulting mixture was stirred at room temperature for an additional 5 h. The reaction was quenched by pouring onto ammonium hydroxide (2 M, 12.5 ml), the resulting inorganic precipitate was removed by filtration, and washed with ethyl acetate (2 × 15 ml). The layers were separated, and the aqueous solution was extracted with ethyl acetate (2 × 15 ml). The combined organic layers were extracted with aq. HCl (10%, 15 ml). The acidic aqueous extracts were washed with ethyl acetate (25 ml), then treated with aq. NaOH (2 M) to pH 10–12 and extracted with ethyl acetate (3 × 25ml). The combined organic extracts were washed with brine (25 ml), dried (Na_2SO_4), filtered and concentrated to give the primary amine, which needed no further purification.

2-Methyl-1-phenylpropan-1-amine

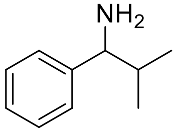

Colourless oil (yield: 49%); ν_{max} (ATR) 2957, 2870, 1465, 1451, 701 cm^{-1}; δ_H (300 MHz, $CDCl_3$) 0.77 (d, 3H, *J* = 6.7, CH_3), 0.98 (d, *J* = 6.7, 3H, CH_3),1.50 (br s, 2H, NH_2), 1.75–1.96 (sym m, 1H, C**H**(CH_3)$_2$), 3.60 (d, 1H, *J* = 7.3, C**H**NH_2), 7.06–7.53 (m, 5H, ArH) ppm; δ_C (75.5 MHz, $CDCl_3$) 18.9 (CH_3), 19.8, 35.5, 62.5, 126.8, 127.0, 128.2 (3 × aromatic CH), 145.5 (aromatic C) ppm; data is in agreement with previously reported data.[2]

Cyclohexyl(phenyl)methanamine

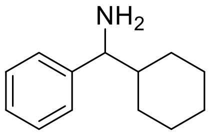

White solid (yield: 23%, m.p.: 99–102 °C); ν_{max} (ATR) 3421, 2922, 2849, 1552, 1453, 1357, 704, 528 cm^{-1}; δ_H (300 MHz, $CDCl_3$) 0.73–1.71 (m, 12H, ring CH & NH_2), 1.71–1.83 (m, 1H, ring CH), 1.86–2.04 (m, 1H, ring CH), 3.60 (d, 1H, *J* = 7.5, C**H**NH_2), 7.10–7.48 (m, 5H, ArH) ppm; δ_C (75.5 MHz, $CDCl_3$) 26.2, 26.4, 29.5, 30.1 (4 × CH_2), 45.2, 61.7 (2 × CH), 126.8, 127.1, 128.2 (3 × aromatic CH), 145.5 (aromatic C) ppm. ^{13}C signal at 145.5 ppm is very weak and was confirmed by HMBC correlation experiments. Carbon assignments were aided by DEPT experiments; data is in agreement with previously reported data.[3] Melting point was not previously reported.

1-(4-Methoxyphenyl)propan-2-amine

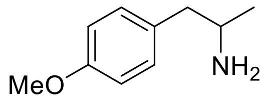

Pale yellow oil (yield: 34%); ν_{max} (ATR) 2957, 2835, 1611, 1510, 1242, 1176, 1033, 802 cm^{-1}; δ_H (300 MHz, $CDCl_3$) 1.10 (d, 3H, *J* = 6.3, CH_3), 2.46 (dd, 1H, *J* = 13.4, 8.0, one of CH_2), 2.65 (dd, 1H, *J* = 13.4, 5.3, one of CH_2), 3.04–3.21 (m, 1H, C**H**NH_2), 3.79 (s, 3H, OCH_3), 6.70–6.94 (m, 2H, ArH), 7.02–7.33 (m, 2H, ArH) ppm; δ_C (75.5 MHz, $CDCl_3$) 23.5, 45.8, 48.6, 55.3, 113.8, 130.2, 131.8, 158.1 ppm; enantiomers separated using Amylose 1 column [conditions: *n*-hexane/*i*PrOH (containing 1% DEA) = 90/10, flow rate = 0.4 ml min^{-1}], R_t = 16.0, R_t = 16.8 min, R_t (ketone) = 14.4 min; data is in agreement with previously reported data.[4]

1,2,3,4-Tetrahydronaphthalen-2-amine

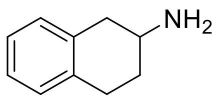

Dark green oil (yield: 48%); ν_{max} (ATR) 3354, 2918, 2841, 741 cm^{-1}; δ_H (300 MHz, $CDCl_3$) 1.51–1.74 [m, 3H, one of C(3)H_2 and NH_2 (1.66, br s)], 1.91–2.10 [m, 1H, one of C(3)H_2], 2.56 [dd, 1H, *J* = 16.1, 9.4, one of C(1)H_2], 2.77–2.94 [m, 2H, C(4)H_2], 3.00 [ddd, 1H, *J* = 16.2, 5.0, 1.3, one of C(1)H_2], 3.18 (tdd, 1H, *J* = 9.5, 5.0, 3.1, C**H**NH_2), 6.87–7.22 (m, 4H, ArH) ppm; δ_C (75.5 MHz, $CDCl_3$) 28.1, 33.0, 39.5 (3 × CH_2), 47.3 (CH), 125.7, 125.8, 128.7, 129.3 (4 × aromatic CH), 135.3, 135.9 (2 × aromatic C) ppm; data is in agreement with previously reported data.[5] Proton and carbon assignments were aided by 2D NMR and DEPT experiments.

1,4-Diphenylbutan-1-amine

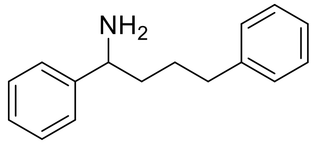

Colourless oil (yield: 16%); ν_{max} (ATR): 2934, 1265, 732, 699 cm^{-1}; δ_H (300 MHz, $CDCl_3$) 1.38–1.98 [m, 6H, 2 × CH_2 & NH_2 (1.69, s)], 2.48–2.71 (m, 2H, CH_2), 3.77–3.97 (m, 1H, C**H**NH_2), 6.91–7.51 (m, 10H, ArH) ppm; δ_C (75.5 MHz, $CDCl_3$), 28.4, 35.8, 39.1 (3 × CH_2), 56.3 (CH), 125.7, 126.4, 127.0, 128.3, 128.4, 128.5 (6 × aromatic CH), 142.3, 146.3 (2 × aromatic C) ppm; HRMS (ES$^+$): $[M+H]^+$ 226.1589 (calculated: 226.1596). This compound is novel and has been fully characterised in this investigation.

(4′-Chlorophenyl)(phenyl)methanamine

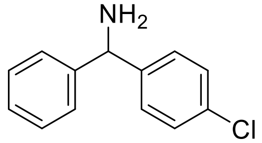

Colourless oil (yield: 37%); δ_H (300 MHz, $CDCl_3$) 1.73 (v br s, 2H, NH_2), 5.12 (br s, 1H, C**H**NH_2), 7.49–7.09 (m, 9H, ArH) ppm; δ_c (75.5 MHz, $CDCl_3$) 59.2, 126.5, 126.8, 127.2, 127.9, 128.3, 128.56, 128.60, 132.65, 144.1, 145.2 ppm; data is in agreement with previously reported data.[6]

Ethyl 3-amino-3-phenylpropanoate

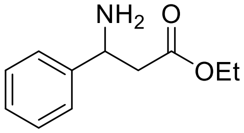

Sulfuric acid (conc., 2.4 ml, 4.42 g, 45.0 mmol) was added to a solution of 3-amino-3-phenylpropanoic acid (2.48 g, 15.0 mmol) in absolute ethanol (50 ml) and heated under reflux for 24 hr. Excess ethanol was evaporated under reduced pressure. The crude product was dissolved in DCM (50 ml) and washed with water (2 × 50 ml), sat. aq. $NaHCO_3$ (2 × 50 ml), brine (50 ml), dried, filtered and concentrated to give the ester as a colourless oil (0.999 g, 34%) which was used without further purification. ν_{max} (ATR) 3381, 1726, 1178, 1031, 699, 539 cm^{-1}; δ_H (300 MHz, $CDCl_3$) 1.23 (t, 3H, *J* = 7.1, $CH_2C\mathbf{H}_3$), 1.77 (br s, 2H, NH_2), 2.66 (d, 2H, *J* = 6.9, $COCH_2$), 4.14 (q, 2H, *J* = 7.1, $OC\mathbf{H}_2CH_3$), 4.42 (dd appears as a t, 1H, *J* = 6.8, $C\mathbf{H}NH_2$), 7.14–7.48 (m, 5H, ArH) ppm; δ_C (75.5 MHz, $CDCl_3$) 14.2, 44.2, 52.6, 60.6, 126.2, 127.4, 128.6, 144.7, 172.1 ppm; data is in agreement with previously reported data.[7]

Synthesis of Sertraline Intermediates

4-(3,4-Dichlorophenyl)-3,4-dihydronaphthalen-1(2H)-one 1

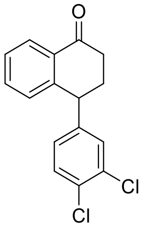

1-Naphthol (5 g, 34.68 mmol), 1,2-dichlorobenzene (32 ml) and $AlCl_3$ (11.56 g, 86.7 mmol) were stirred under N_2 at 110 °C for 2 h. The solution was cooled to room temperature and poured into a 1:1 mixture of ice and 1M HCl solution (30 ml). The aqueous layer was extracted with DCM (2 x 200 ml). The combined organic layer was washed with H_2O (100 ml), then stirred with Celite® (6.7 g) and activated charcoal (5 g) and filtered. The solvent was removed *in vacuo.* The crude brown liquid was purified by flash chromatography (hexane: ethyl acetate 1:0 to 17:3) and recrystallised from hexane to give the *pure* product as a colourless solid (yield: 6.20 g, 61%). ν_{max} (ATR) 1672, 771 cm^{-1}; δ_H (300 MHz, $CDCl_3$) 2.20−2.32 (m, 1H), 2.42−2.53 (m, 1H), 2.57−2.77 (m, 2H), 4.27 (dd, J = 8.0, 4.6 Hz, 1H), 6.92-6.98 (m, 2H), 7.23 (d, *J* = 2 Hz, 2H), 7.36-7.42 (m, 2H), 7.47 (dt, *J* = 7.2, 1.5 Hz, 1H), 8.13 (dd, J = 7.9, 1.8 Hz, 1H) ppm; δ_C (75.5 MHz, $CDCl_3$) 31.7, 36.5, 44.6, 127.4, 127.7, 128.0, 129.3, 130.6, 130.5, 131.0, 132.7, 132.8, 133.9, 144.0, 144.8, 197.3; enantiomers separated using Chiralcel OJ-H [conditions: n-hexane/*i*PrOH (containing 1% DEA) 90:10, 0.5 ml min-1, 25°C, λ = 235 nm], R_t = 23.8 (*4R*), 26.5 (*4S*) min; data is in agreement with previously reported data.[8]

Reduction of ketone:[9] A solution of ketone (72 mmol) in MeOH (400 ml) was cooled to 0 °C. $NaBH_4$ (72 mmol) was added portion wise. The solution was allowed to warm to room temperature and stirred until TLC showed disappearance of the starting material (≈2 hr). The reaction mixture was acidified to pH 5 using 10% aqueous HCl solution. The volatiles were removed *in vacuo* and the aqueous layer was washed

with EtOAc (2 x 100 ml). The combined organic layer was washed with brine (200 ml), dried over $MgSO_4$, filtered and the solvent removed *in vacuo* to give a mixture of the *cis*- and *trans*- diastereomers (42:58).

Diastereomers were separated *via* repeated flash chromatography (80:20 hexane:Et_2O) to give *cis*-**7a**: 7.20 g (34%, least polar), *trans*-7b: 6.47 g (31%, most polar) with the remainder of the recovered material being a mixture of the two diastereomers

***cis*-4-(3,4-Dichlorophenyl)-1,2,3,4-tetrahydronaphthalen-1-ol *cis*-7a**

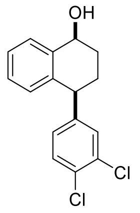

ν_{max} (ATR) 3336, 2939, 2862, 1468, 1397, 1030, 764 cm^{-1}; δ_H (300 MHz, $CDCl_3$) 1.84 (br s, 1H), 1.94– 2.16 (m, 4H), 3.99 (t, J = 6.9 Hz, 1H), 4.86 (t, *J* = 4.2 Hz, 1H), 6.82 (d, J = 7.2 Hz, 1H), 6.99 (dd, J = 8.2, 1.9 Hz, 1H), 7.17 (dt, J = 7.2, 1.5 Hz, 1H), 7.21-7.31 (m, 2H), 7.37 (d, J = 8.2 Hz, 1H), 7.46 (dd, J = 7.6, 1.2 Hz, 1H) ppm; δ_C (75.5 MHz, $CDCl_3$) 28.2, 30.1, 45.1, 67.9, 127.1, 128.2, 128.3, 129.0, 129.8, 130.3, 130.4, 130.7, 132.4, 138.4, 139.0, 147.0 ppm; data is in agreement with previously reported data.[8]

***trans*-4-(3,4-Dichlorophenyl)-1,2,3,4-tetrahydronaphthalen-1-ol *trans*-7b**

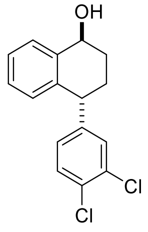

ν_{max} (ATR) 3340, 2938, 2862, 1468, 1397, 1029, 764 cm^{-1}; δ_H (300 MHz, $CDCl_3$) 1.65–1.90 (m, 2H), 1.95–2.21 (m, 2H), 2.24–2.45 (m, 1H), 4.12 (t, J = 6.3 Hz, 1H), 4.80-4.94 (m, 1H), 6.71-6.90 (m, 2H), 7.11 (d, *J* = 2 Hz, 1H), 7.16 (dt, *J* = 7.6, 1.4 Hz, 1H), 7.22–7.35 (m, 2H), 7.54 (d, J = 7.6 Hz, 1H) ppm; δ_C (75.5 MHz, $CDCl_3$) 28.9, 30.0, 44.5, 68.2, 127.2, 127.9, 128.0, 128.1, 129.9, 130.2, 130.3, 130.5, 132.3, 137.8, 139.6, 146.8 ppm; data is in agreement with previously reported data.[10]

General procedure for the synthesis of amines.[11]

In a 2-necked round-bottomed flask, diphenyl phosphoryl azide (0.4 ml, 1.85 mmol, 1.2 equiv.) and the relevant alcohol (0.45g, 1.54 mmol) in dry toluene (15 ml) were stirred under N_2 at 0 °C for 10 minutes. 1,8-Diazabicyclo[5.4.0]undec-7-ene (1.85 mmol, 1.2 equiv.) was added dropwise over 20 mins. Upon addition, the orange solution turned cloudy. It was stirred at 0 °C for 2 hr, and then allowed to warm to room temperature and stirred for a further 12 hr. The solvent was removed in vacuo and the oily residue was passed through a silica plug to give a viscous, colourless oil. This was dissolved in THF (5 ml) and PPh_3 (0.485 g, 1.85 mmol, 1.2 equiv.) and H_2O (0.055 ml, 2 equiv.) were added. The solution was heated under reflux for 4 hr and allowed to cool. The solvent was removed under reduced pressure.

Removal of the triphenyl phosphine oxide by-product: the residue was dissolved in a 50:50 mix of hexane and Et_2O (50 ml) and stored overnight at -20°C. The white precipitate was removed by filtration and the filtrate concentrated under vacuum. The residue was dissolved in DCM (10 ml) and 5M aqueous HCl solution was added dropwise until pH 1. The resulting white precipitate was collected by filtration. The solid was resuspended in H_2O (50 ml) and the pH was adjusted to 10 with 1M aqueous NaOH solution. The mixture was stirred for 30 mins, then extracted using ethyl acetate (2 x 50 ml). The combined organic layer was washed with water (100 ml), brine (100 ml), dried (Na_2SO_4), filtered and concentrated *in vacuo* to give the *pure* product.

***trans*-4-(3,4-Dichlorophenyl)-1,2,3,4-tetrahydronaphthalen-1-amine *trans*-8a**

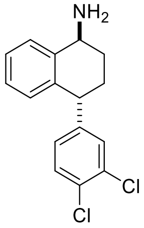

Prepared from alcohol *cis*-**15a** to give a brown oil (yield: 73%); ν_{max} (ATR) 2929, 2857, 1464, 763 cm^{-1}; δ_H (300 MHz, $CDCl_3$) 1.52-1.68 (m, 3H), 1.74-1.86 (m, 1H), 2.04-2.16 (m, 1H), 2.24-2.36 (m, 1H), 4.06-4.15 (m, 2H), 6.80 (d, *J* = 7.7 Hz, 1H), 6.88 (dd, J = 8.3, 2.1 Hz, 1H), 7.08-7.16 (m, 2H), 7.21-7.28 (m, 1H), 7.33 (d, J = 8.3 Hz, 1H), 7.51 (d, J = 7.7 Hz, 1H) ppm; δ_C (75.5 MHz, $CDCl_3$) 29.9, 31.5, 45.0, 49.5, 127.0, 127.8, 128.1, 129.9, 130.1, 130.2, 130.6, 132.3, 137.6, 141.7, 147.4 ppm; enantiomers separated using Chiralcel OJ-H [conditions: n-hexane/*i*PrOH (containing 1% DEA) 90:10, 0.5 ml min^{-1}, 25°C, λ = 230 nm], R_t = 15.7 (1S, *4R*), 18.6 (*1R*, *4S*) min; data is in agreement with previously reported data.[12]

***cis*-4-(3,4-Dichlorophenyl)-1,2,3,4-tetrahydronaphthalen-1-amine *cis*-8b**

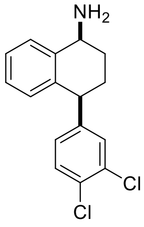

Prepared from alcohol *trans*-**15b** to give an off-white solid (yield: 76%); ν_{max} (ATR) 2940, 2886, 1472, 1140, 777 cm^{-1}; δ_H (300 MHz, $CDCl_3$) 1.63 (br s, 2H), 1.71-1.82 (m, 1H), 1.92-2.14 (m, 3H), 3.99-4.04 (m, 1H), 4.04-4.11 (m, 1H), 6.80 (d, *J* = 7.7 Hz), 6.94 (dd, *J* = 8.3, 2.0 Hz), 7.11 (td, *J* = 7.5, 1.2 Hz, 1H), 7.20-7.26 (m, 1H), 7.35 (d, *J* = 8.2 Hz, 1H), 7.43 (d, *J* = 7.6 Hz, 1H) ppm; δ_C (75.5 MHz, $CDCl_3$) 28.7, 30.7, 45.0, 49.2, 126.9, 127.0, 128.2, 128.5, 129.8, 130.1, 130.2, 130.7, 132.3, 137.7, 141.4, 147.3 ppm; enantiomers were separated using Chiralcel OJ-H [conditions: n-hexane/iPrOH (containing 1% DEA) 90:10, 0.5 ml min^{-1}, 25°C, λ = 235 nm], Rt = 15.0 (1S, *4S*), 17.0 (1R, *4R*) min; data is in agreement with previously reported data.[12]

When a mix of **8a** and **8b** was used as substrate, the amine stereoisomers could not be separated but the ketone product 1 enantiomers were separated using Chiralcel IA [conditions: n-hexane/iPrOH (containing 1% DEA) 97:3, 0.6 ml min-1, 25°C, λ = 230 nm], Rt = 21.8 (4R), 23.7 (4S) min.

References:

1 Miriyala, B., Bhattacharyya, S. & Williamson, J. S. Chemoselective reductive alkylation of ammonia with carbonyl compounds: synthesis of primary and symmetrical secondary amines. *Tetrahedron* **60**, 1463-1471, (2004).

2 Wang, C., Pettman, A., Basca, J. & Xiao, J. A versatile catalyst for reductive amination by transfer hydrogenation. *Angew. Chem. Int. Ed.* **49**, 7548-7552, (2010).

3 Weiberth, F. J. & Hall, S. S. Tandem alkylation-reduction of nitriles. Synthesis of branched primary amines. *J. Org. Chem.* **51**, 5338-5341, (1986).

4 Vahermo, M., Suominen, T., Leinonen, A. & Yli-Kauhaluoma, J. Synthesis and characterization of hydroxylated mesocarb metabolites for doping control. *Arch. Pharm.* **342**, 201-209, (2009).

5 Bondarev, O. & Bruneau, C. Indirect and direct catalytic asymmetric reductive amination of 2-tetralone. *Tetrahedron: Asymmetry* **21**, 1350-1354, (2010).

6 Terrasson, V., Marque, S., Georgy, M., Campagne, J.-M. & Prim, D. Lewis Acid-Catalyzed Direct Amination of Benzhydryl Alcohols. *Adv. Synth. Catal.* **348**, 2063-2067, (2006).

7 Poon, D., Brinner, K. & Doughan, B. Scalable Synthesis of β-Amino Esters via Reformatsky Reaction with N-tert-Butanesulfinyl Imines. *Synlett* **2009**, 991-993, (2009).

8 Lee, S. H. *et al.* Stereoselective amination of chiral benzylic ethers using chlorosulfonyl isocyanate: total synthesis of (+)-sertraline. *J. Org. Chem.* **76**, 10011-10019, (2011).

9 Shao, L. *et al.* Synthesis and pharmacological evaluation of 4-(3,4-dichlorophenyl)-N-methyl-1,2,3,4-tetrahydronaphthalenyl amines as triple reuptake inhibitors. *Bioorg. Med. Chem.* **19**, 663-676, (2011).

10 Lautens, M. & Rovis, T. Selective functionalization of 1,2-dihydronaphthalenols leads to a concise, stereoselective synthesis of sertraline. *Tetrahedron* **55**, 8967-8976, (1999).

11 Fernández, R., Ros, A., Magriz, A., Dietrich, H. & Lassaletta, J. M. Enantioselective synthesis of cis-α-substituted cycloalkanols and trans-cycloalkyl amines thereof. *Tetrahedron* **63**, 6755-6763, (2007).

12 Balavoine, F., Batch, A. & Rolland, C. Processes for preparing desmethylsertraline or a pharmaceutically acceptable salt thereof. (2008).

3. HPLC chromatograms

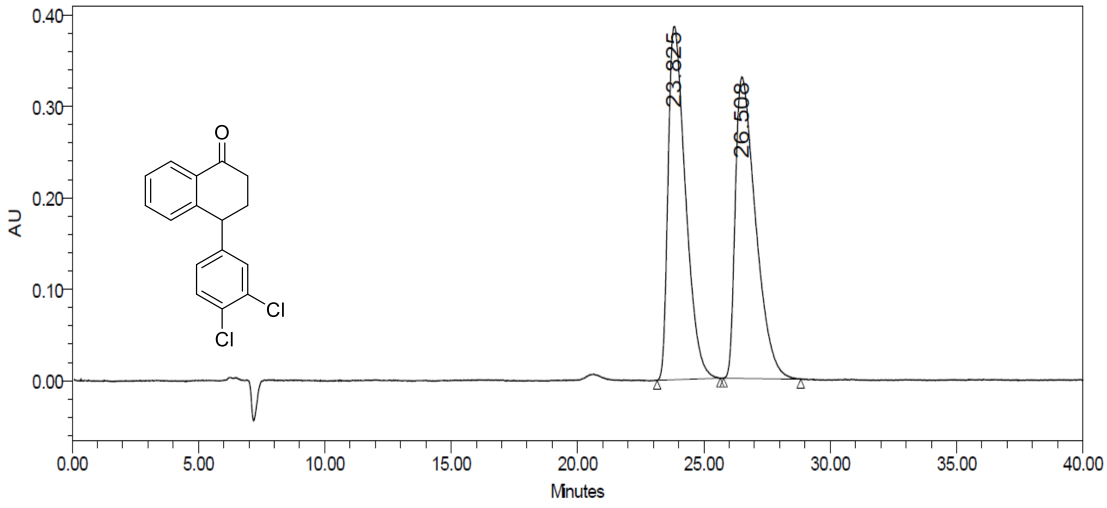

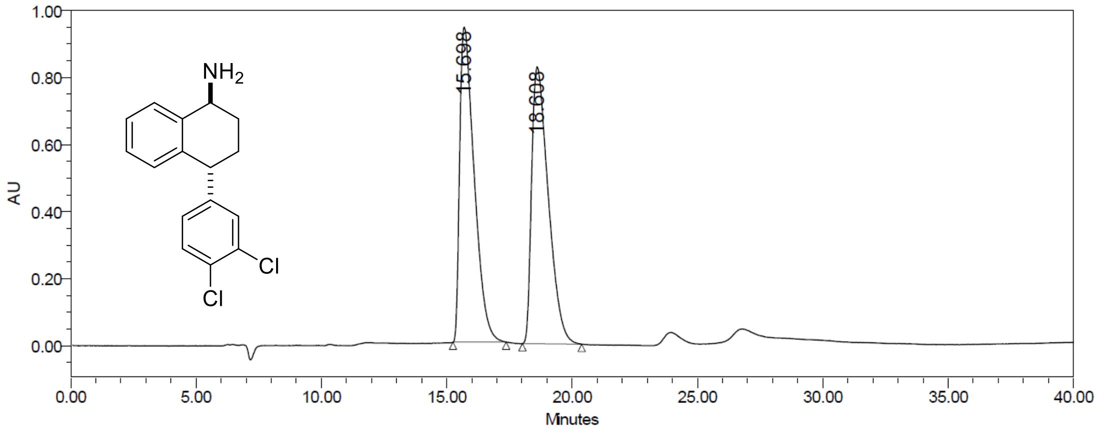

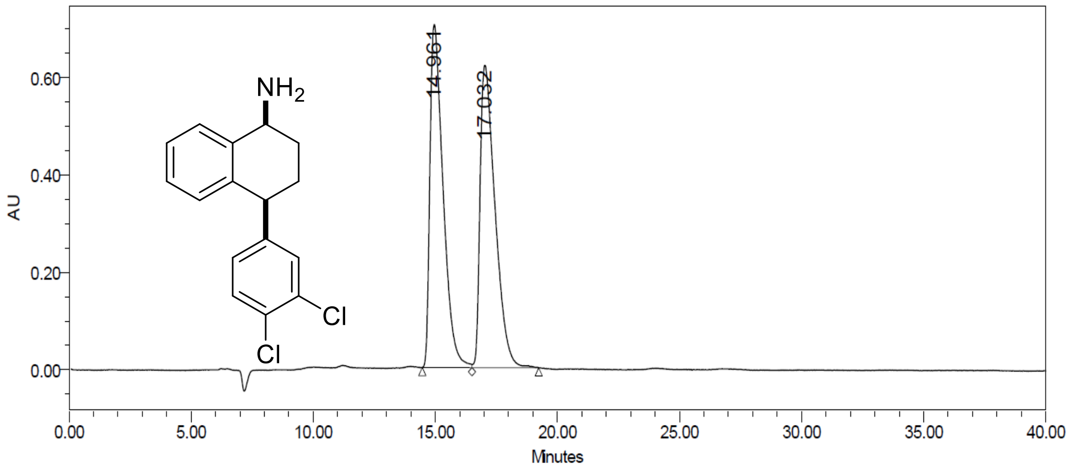

Chromatograms from Tables

Table 1, P-ω-TA

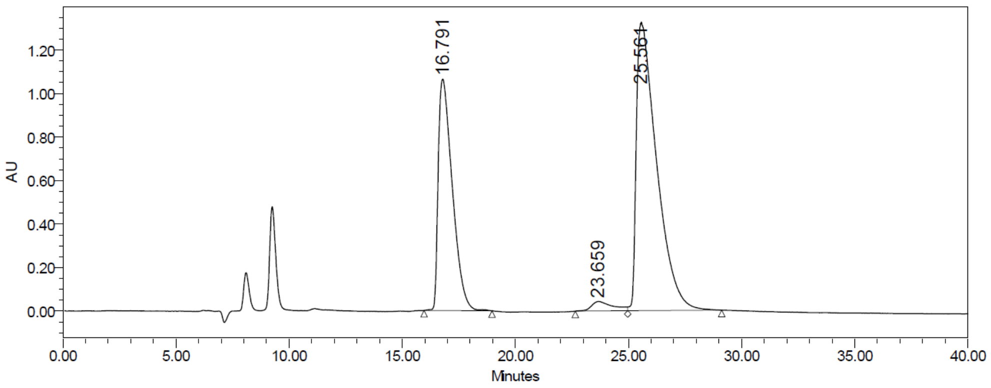

Table 1, Cv-ω-TA

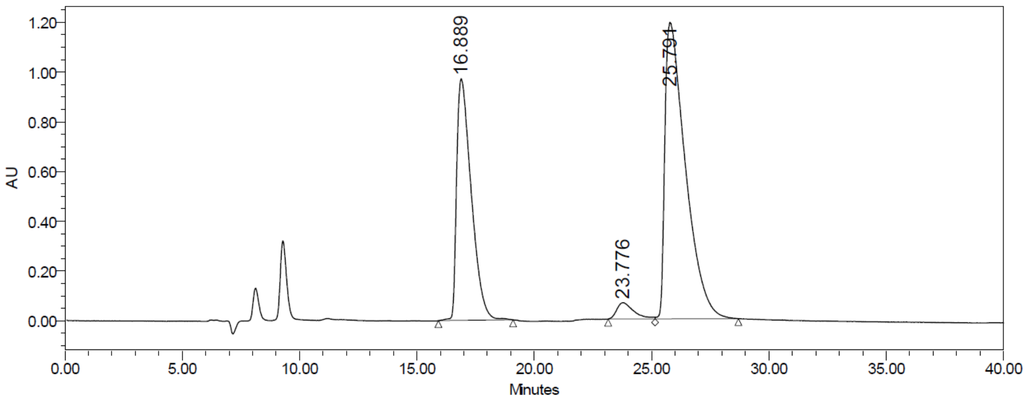

Table 2, P-ω-TA

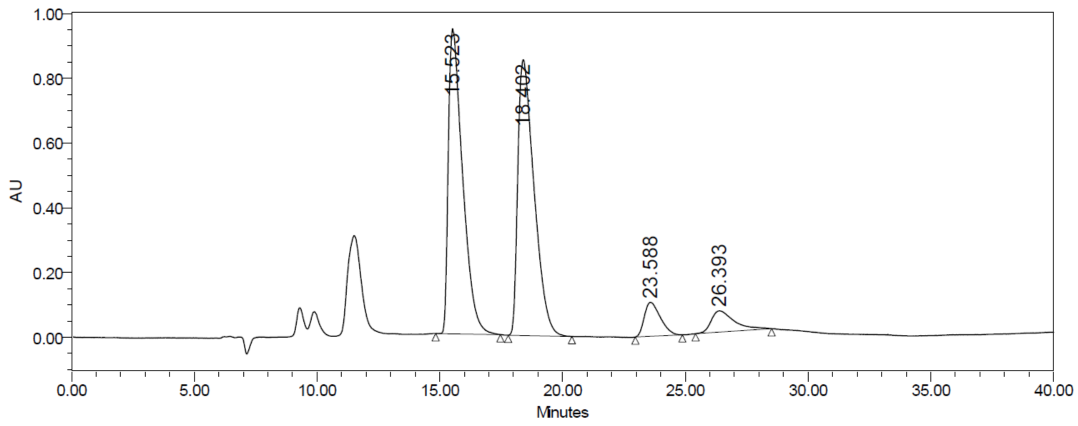

Table 2, Cv-ω-TA

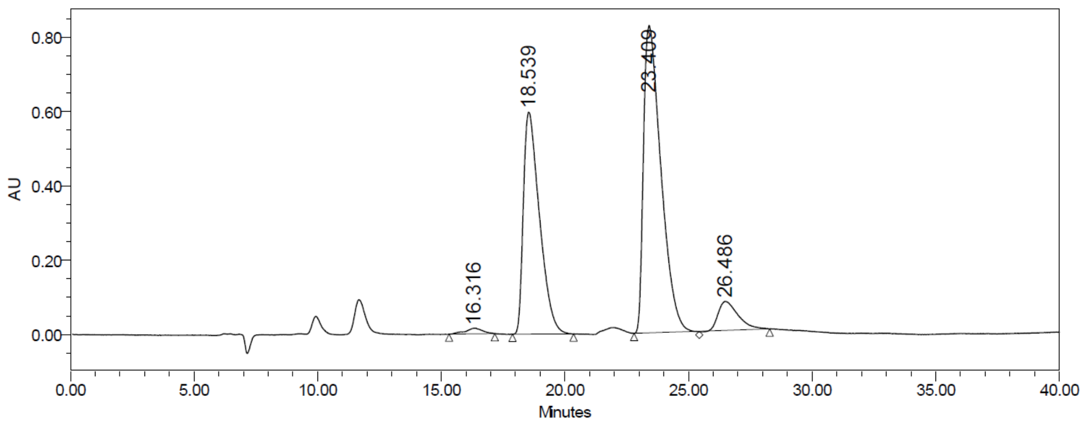

Table 3, P-ω-TA

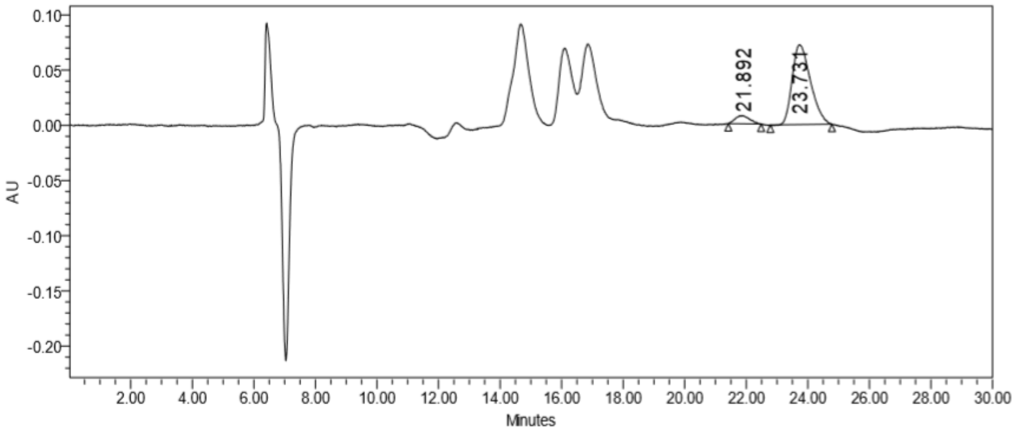

Table 3 Cv-ω-TA

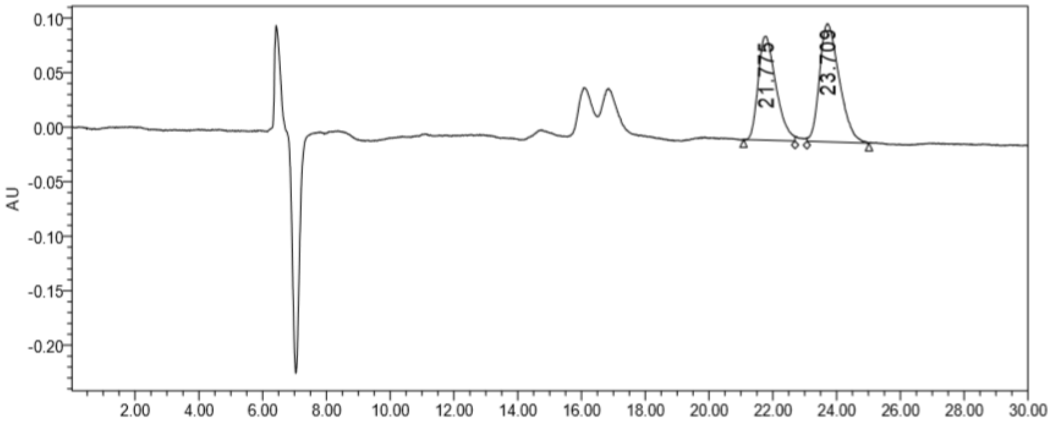

4. ^{1}H & ^{13}C NMR spectra

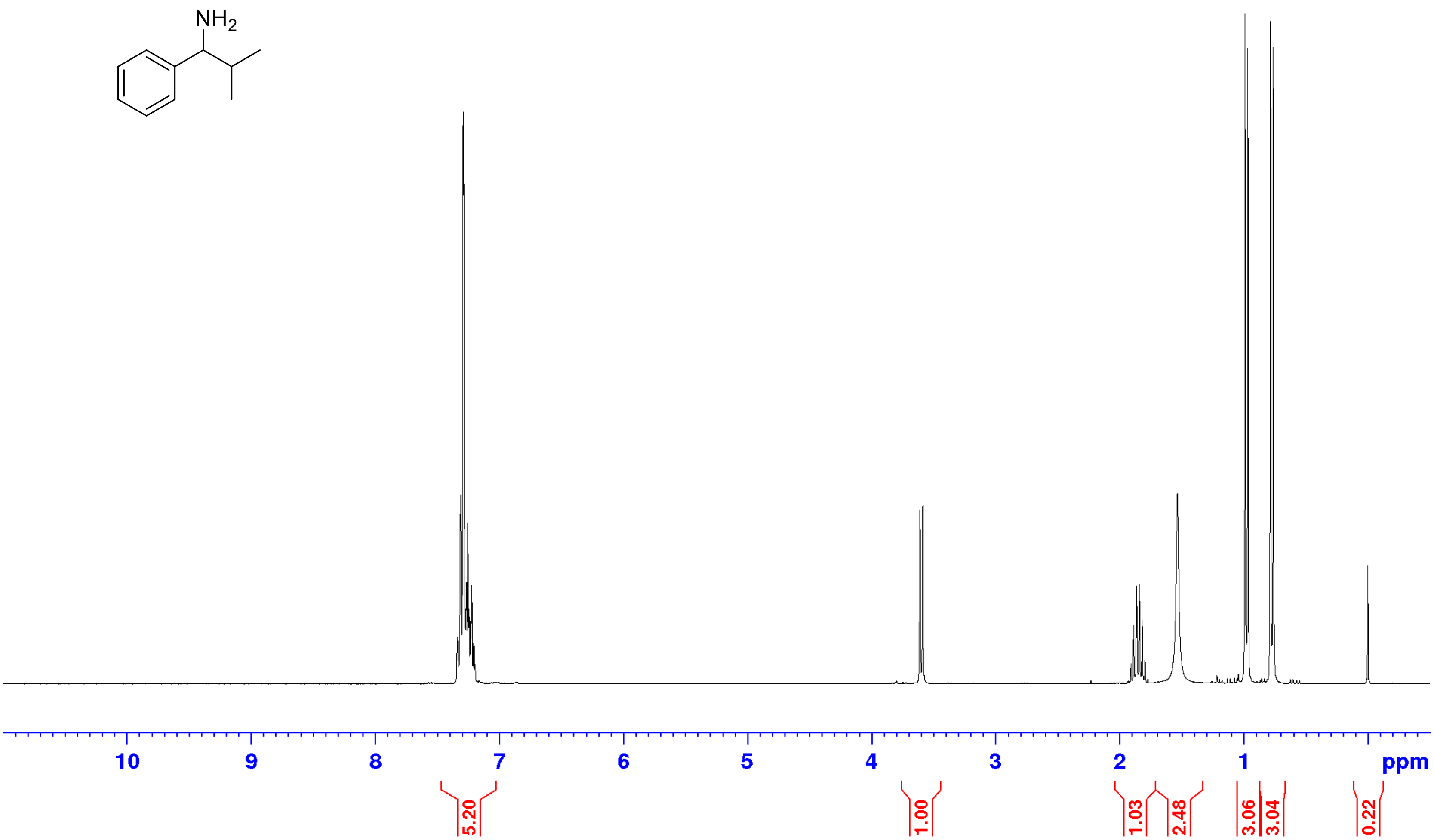

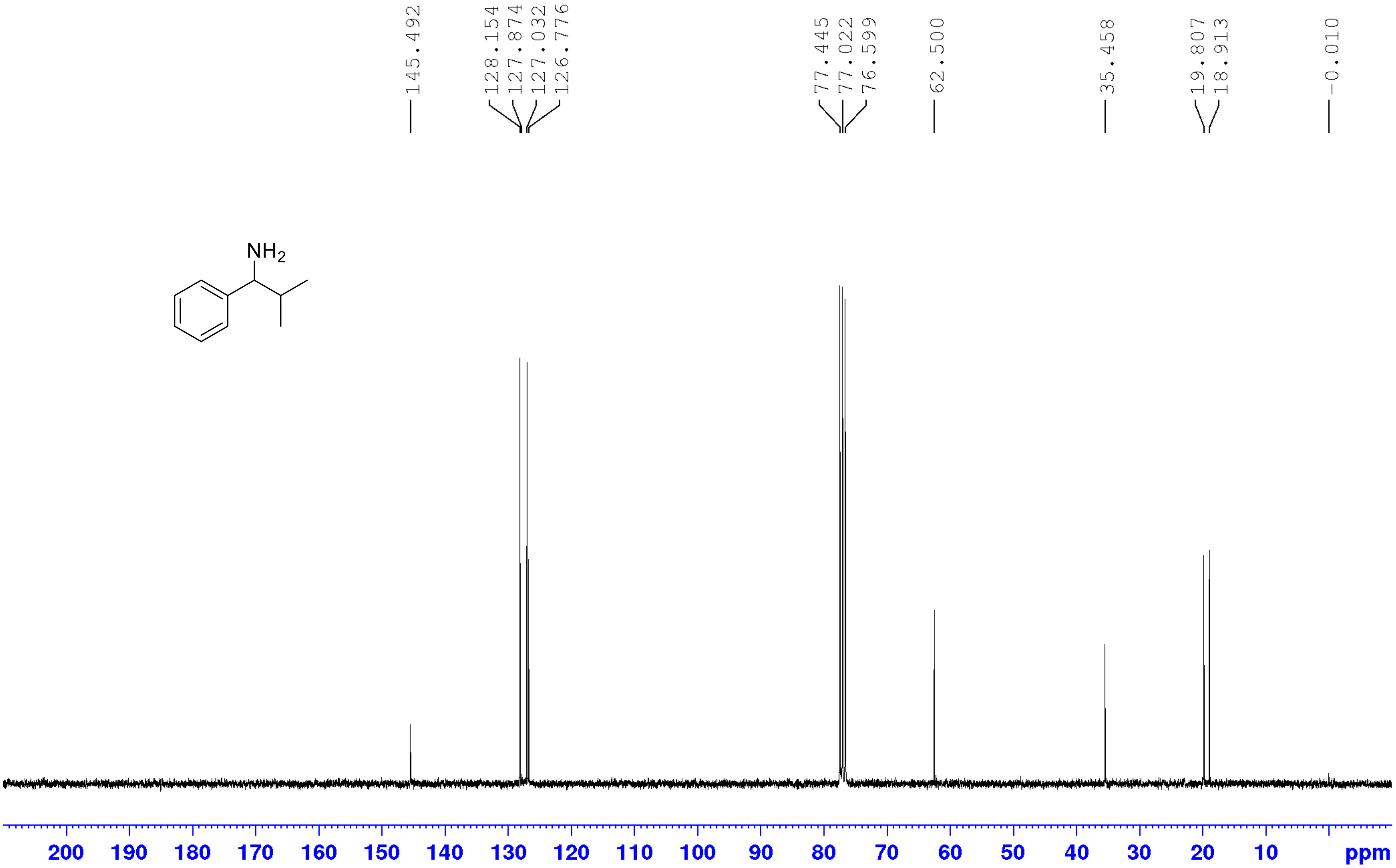

145.492
128.154
127.874
127.032
126.776
77.445
77.022
76.599
62.500
35.458
19.807
18.913
-0.010
NH2
200
190
180
170
160
150
140
130
120
110
100
90
80
70
60
50
40
30
20
10
ppm

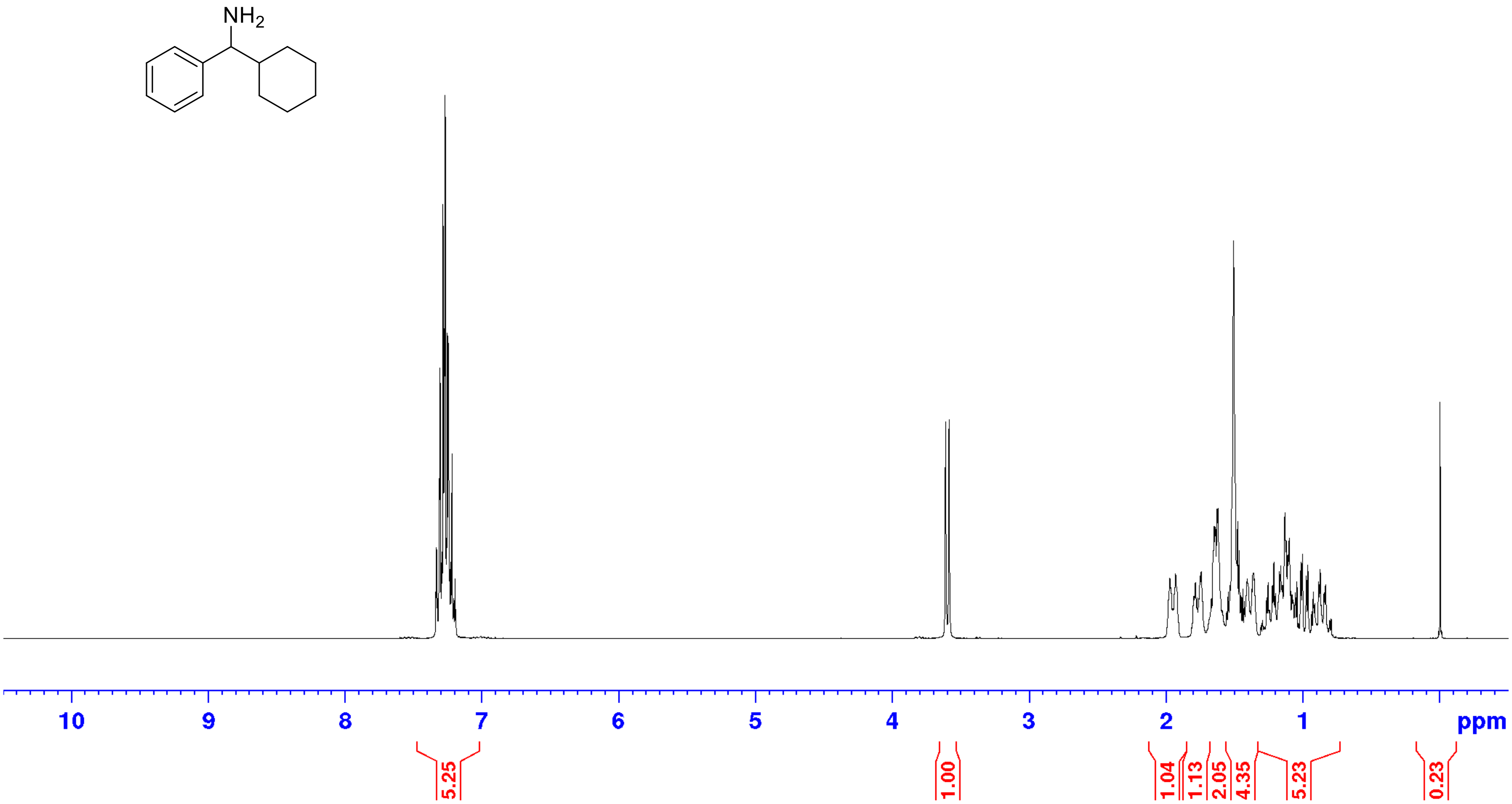
NH2
10
9
8
7
6
5
4
3
2
1
ppm
5.25
1.00
1.04
1.13
2.05
4.35
5.23
0.23

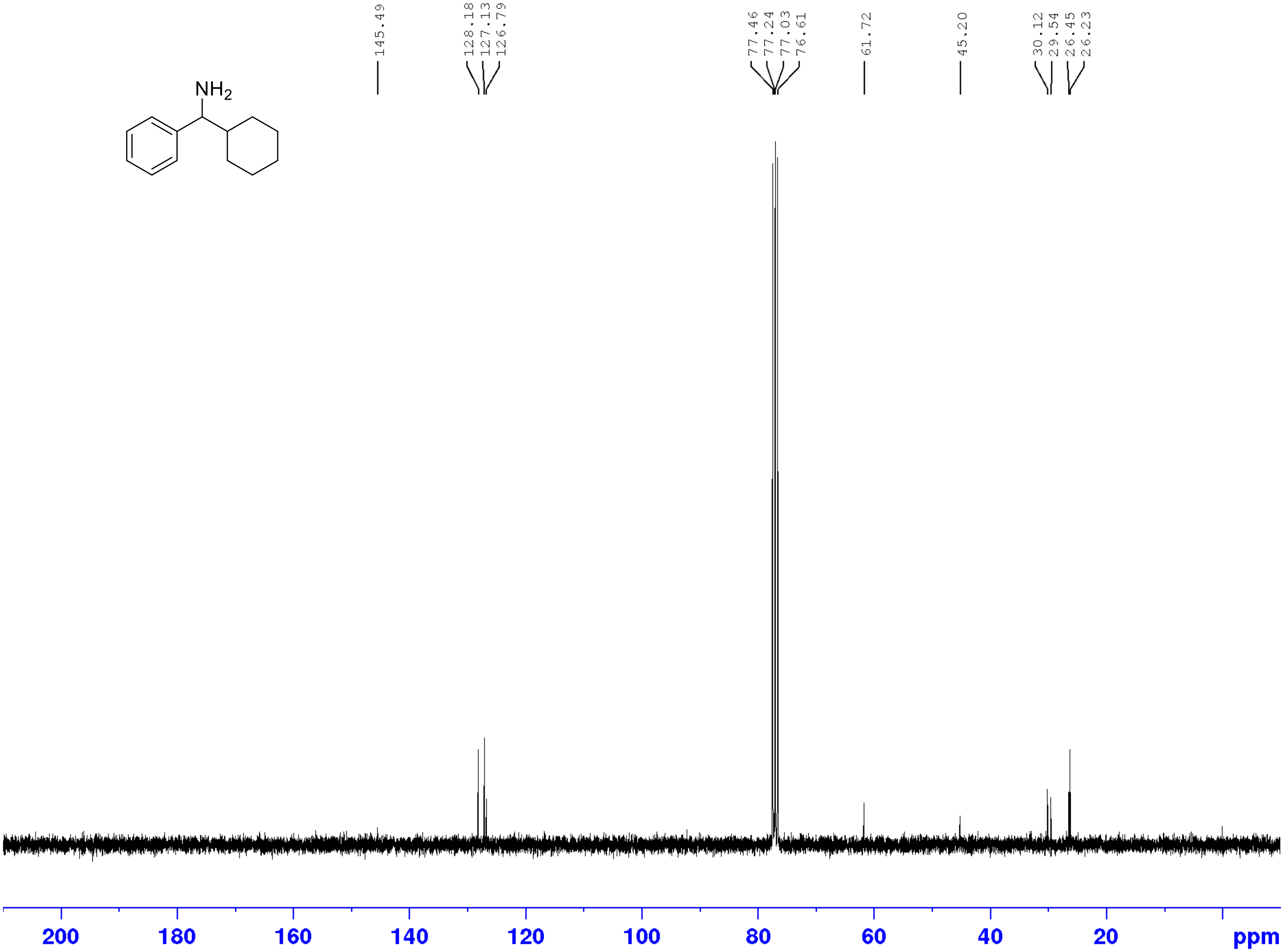
NH2
145.49
128.18
127.13
126.79
77.46
77.24
77.03
76.61
61.72
45.20
30.12
29.54
26.45
26.23
200
180
160
140
120
100
80
60
40
20
ppm

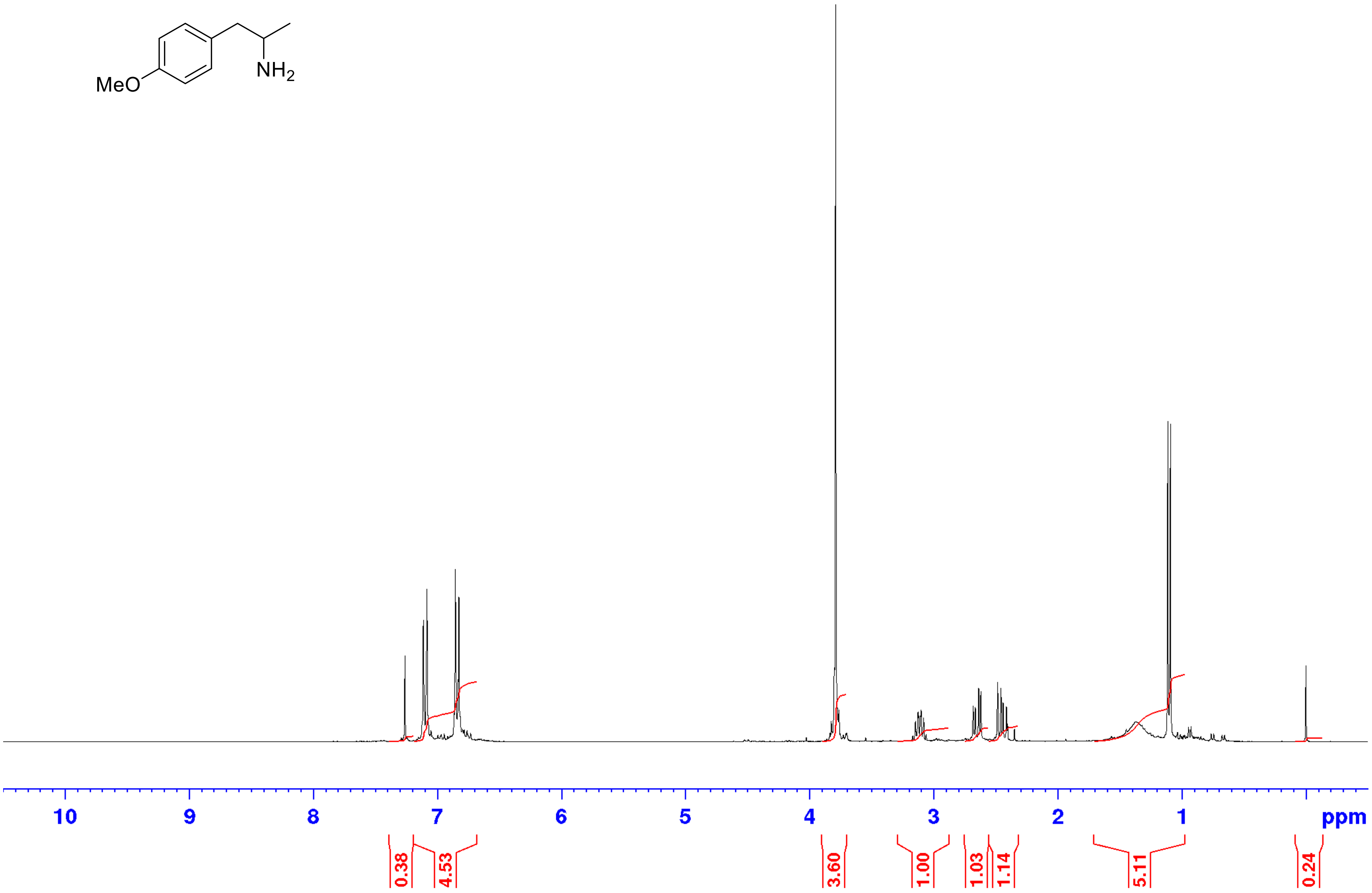
MeO
NH2
10
9
8
7
6
5
4
3
2
1
ppm
0.38
4.53
3.60
1.00
1.03
1.14
5.11
0.24

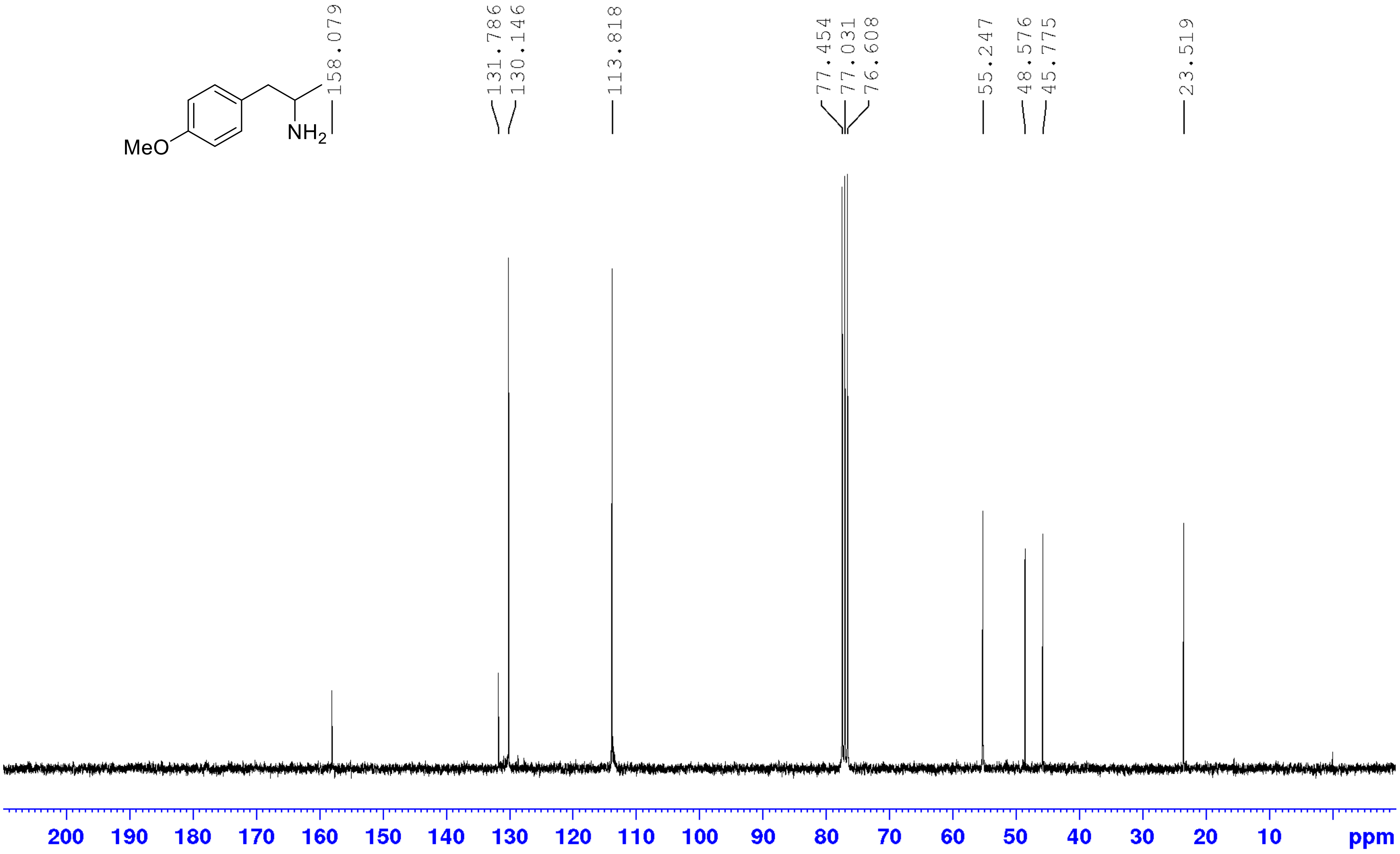

MeO
NH2
158.079
131.786
130.146
113.818
77.454
77.031
76.608
55.247
48.576
45.775
23.519
200
190
180
170
160
150
140
130
120
110
100
90
80
70
60
50
40
30
20
10
ppm

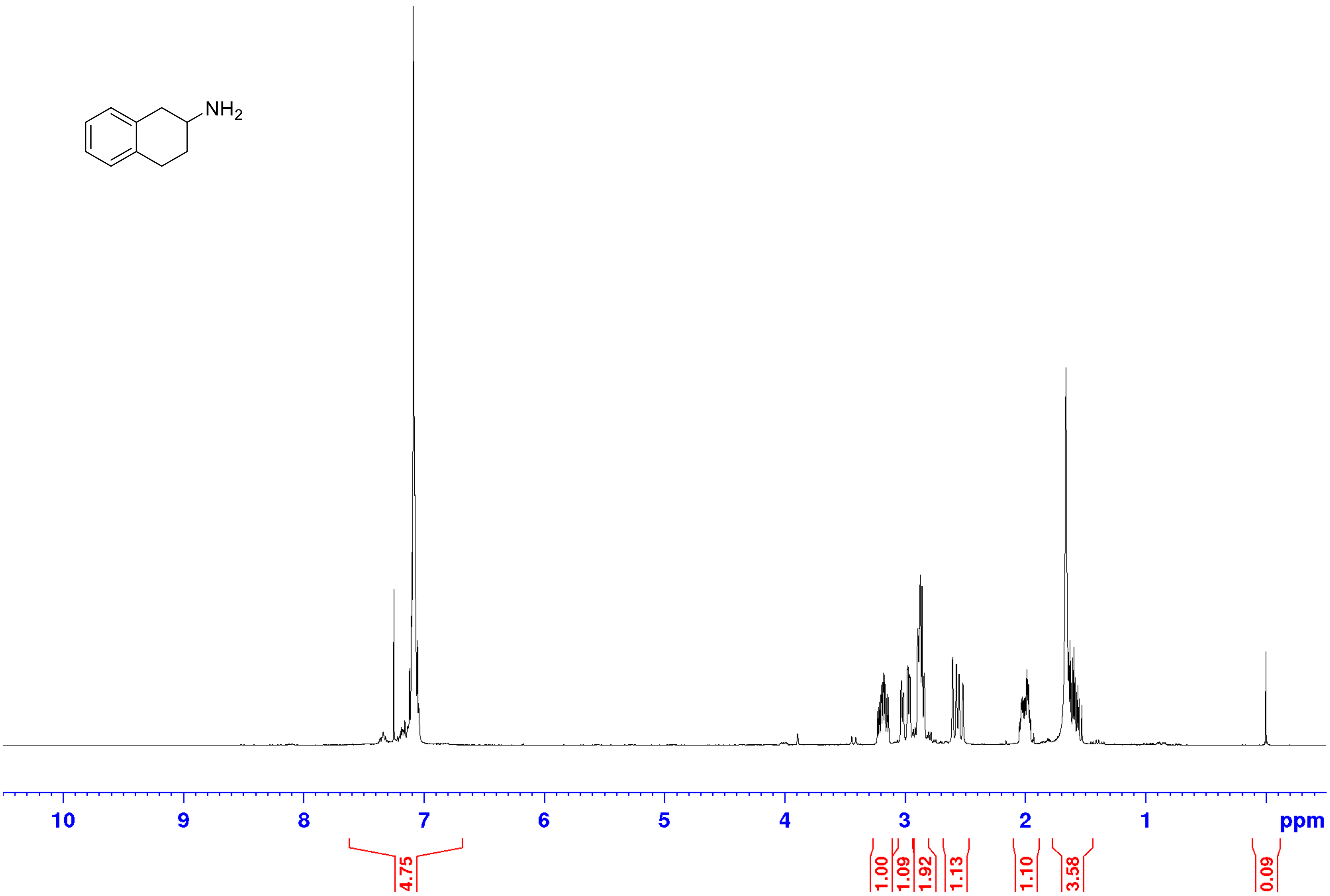
NH2
10
9
8
7
6
5
4
3
2
1
ppm
4.75
1.00
1.09
1.92
1.13
1.10
3.58
0.09

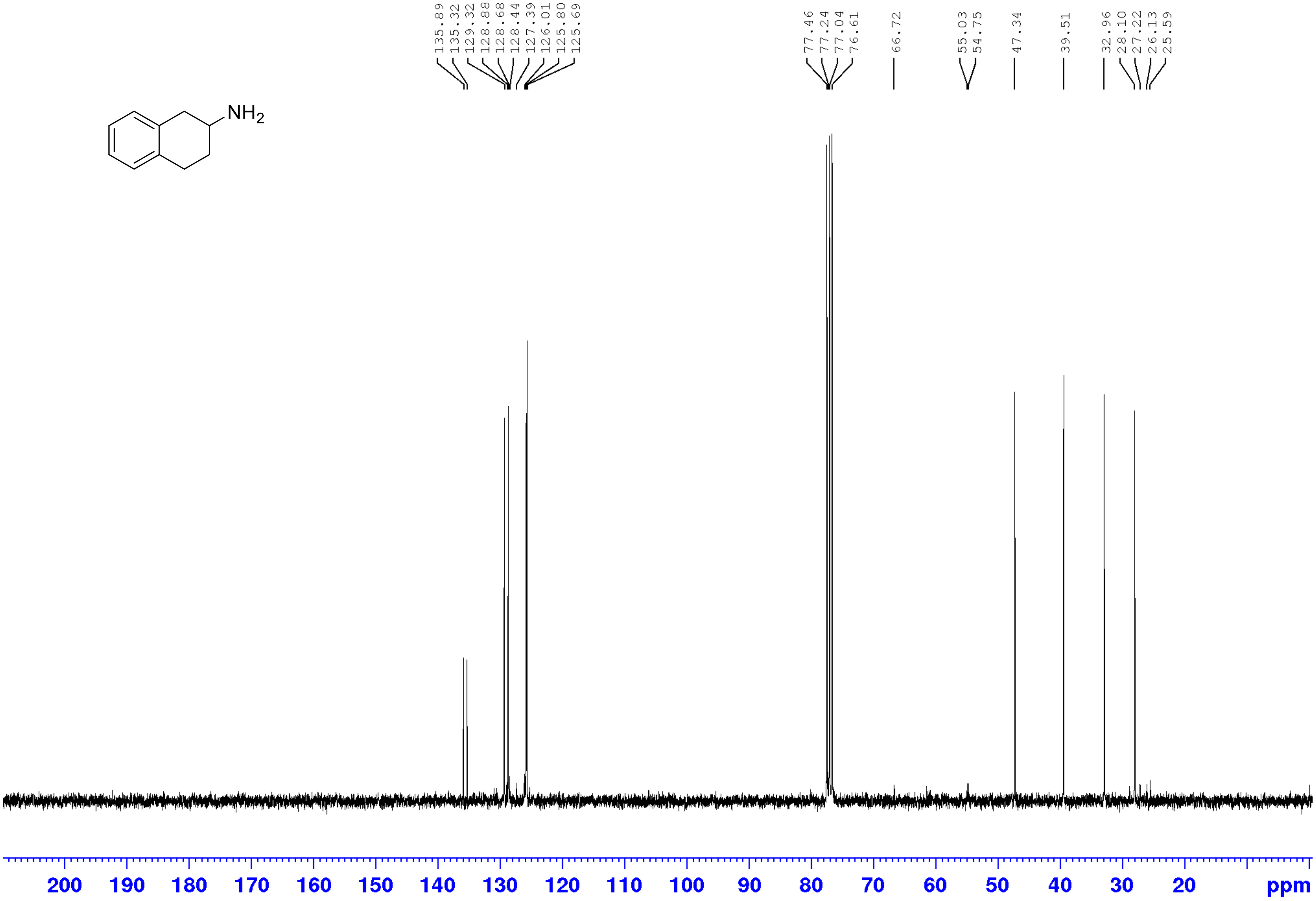
NH$_2$
135.89
135.32
129.32
128.88
128.68
128.44
127.39
126.01
125.80
125.69
77.46
77.24
77.04
76.61
66.72
55.03
54.75
47.34
39.51
32.96
28.10
27.22
26.13
25.59
200 190 180 170 160 150 140 130 120 110 100 90 80 70 60 50 40 30 20
ppm

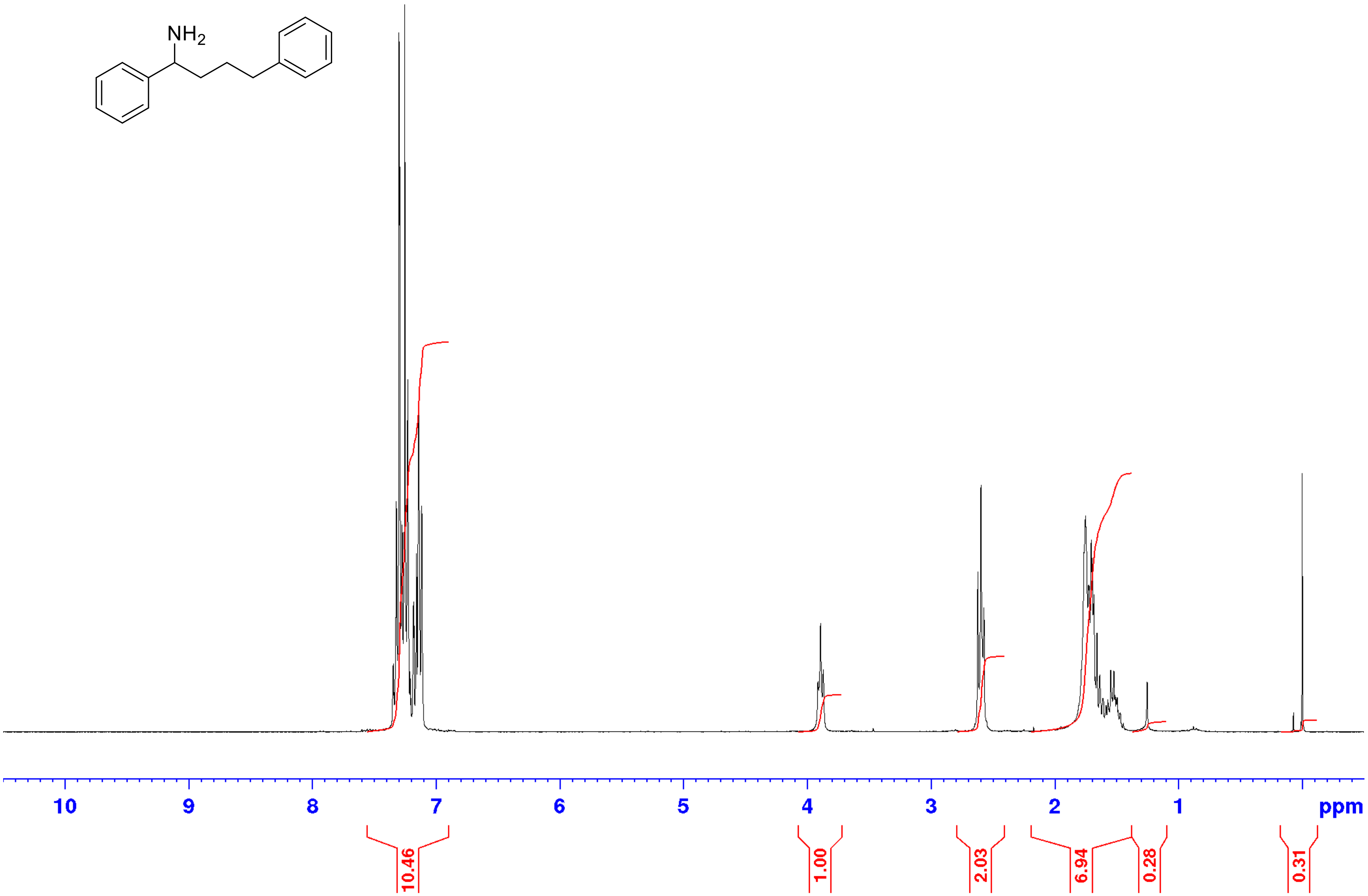
NH2
10
9
8
7
6
5
4
3
2
1
ppm
10.46
1.00
2.03
6.94
0.28
0.31

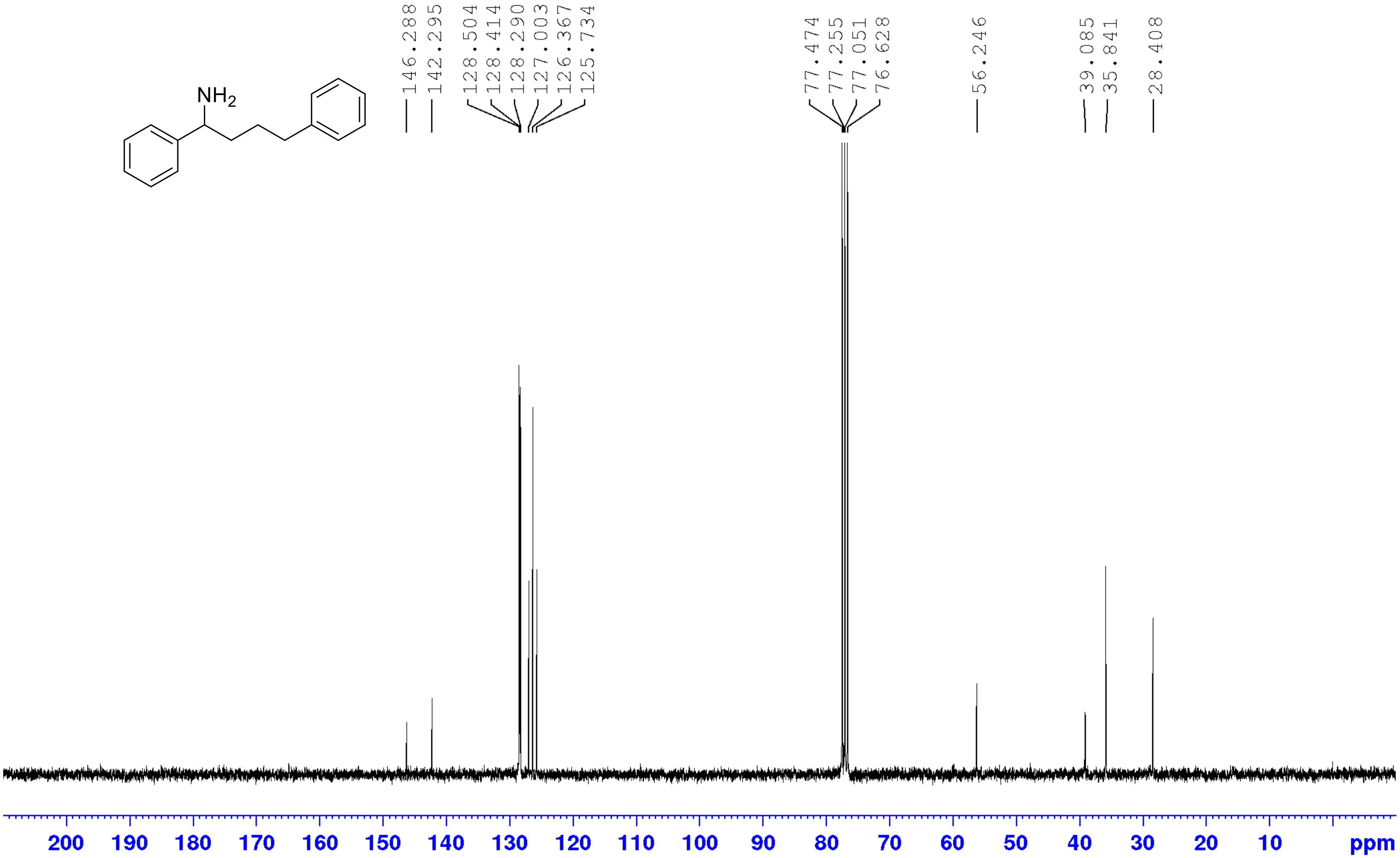
NH2
146.288
142.295
128.504
128.414
128.290
127.003
126.367
125.734
77.474
77.255
77.051
76.628
56.246
39.085
35.841
28.408
200 190 180 170 160 150 140 130 120 110 100 90 80 70 60 50 40 30 20 10 ppm

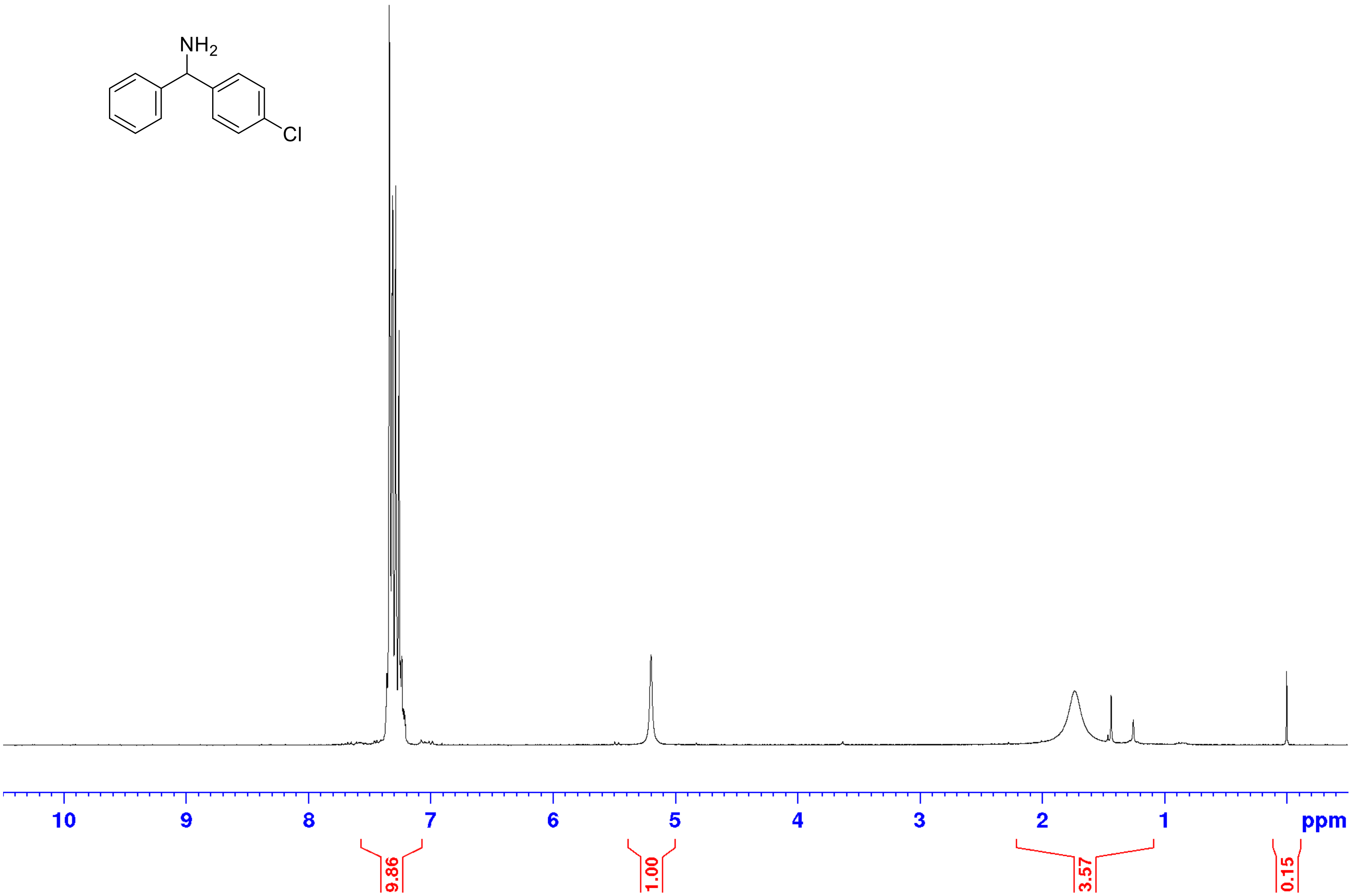
NH2
Cl
10
9
8
7
6
5
4
3
2
1
ppm
9.86
1.00
3.57
0.15

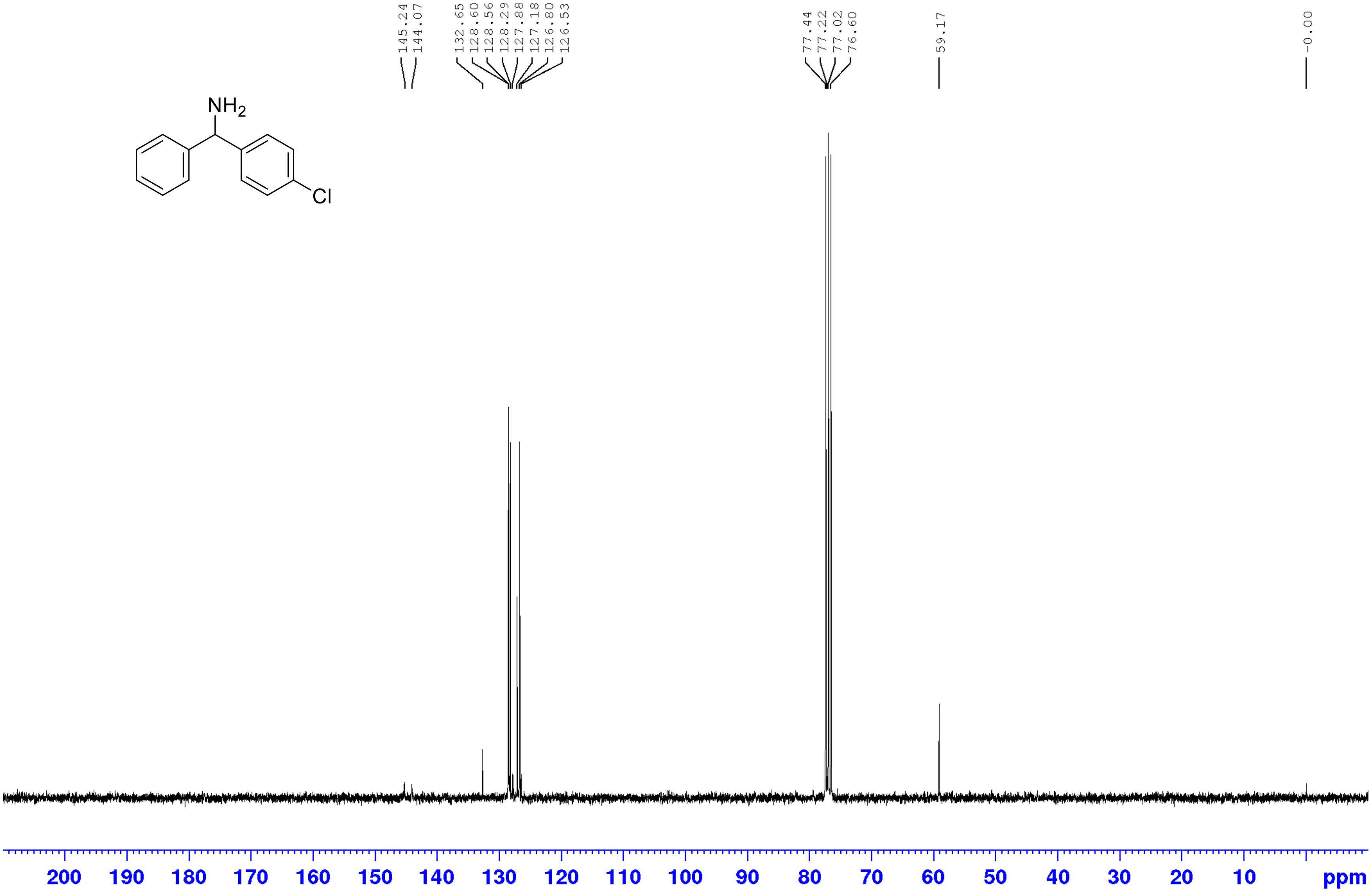
145.24
144.07
132.65
128.60
128.56
128.29
127.88
127.18
126.80
126.53
77.44
77.22
77.02
76.60
59.17
-0.00
200
190
180
170
160
150
140
130
120
110
100
90
80
70
60
50
40
30
20
10
ppm

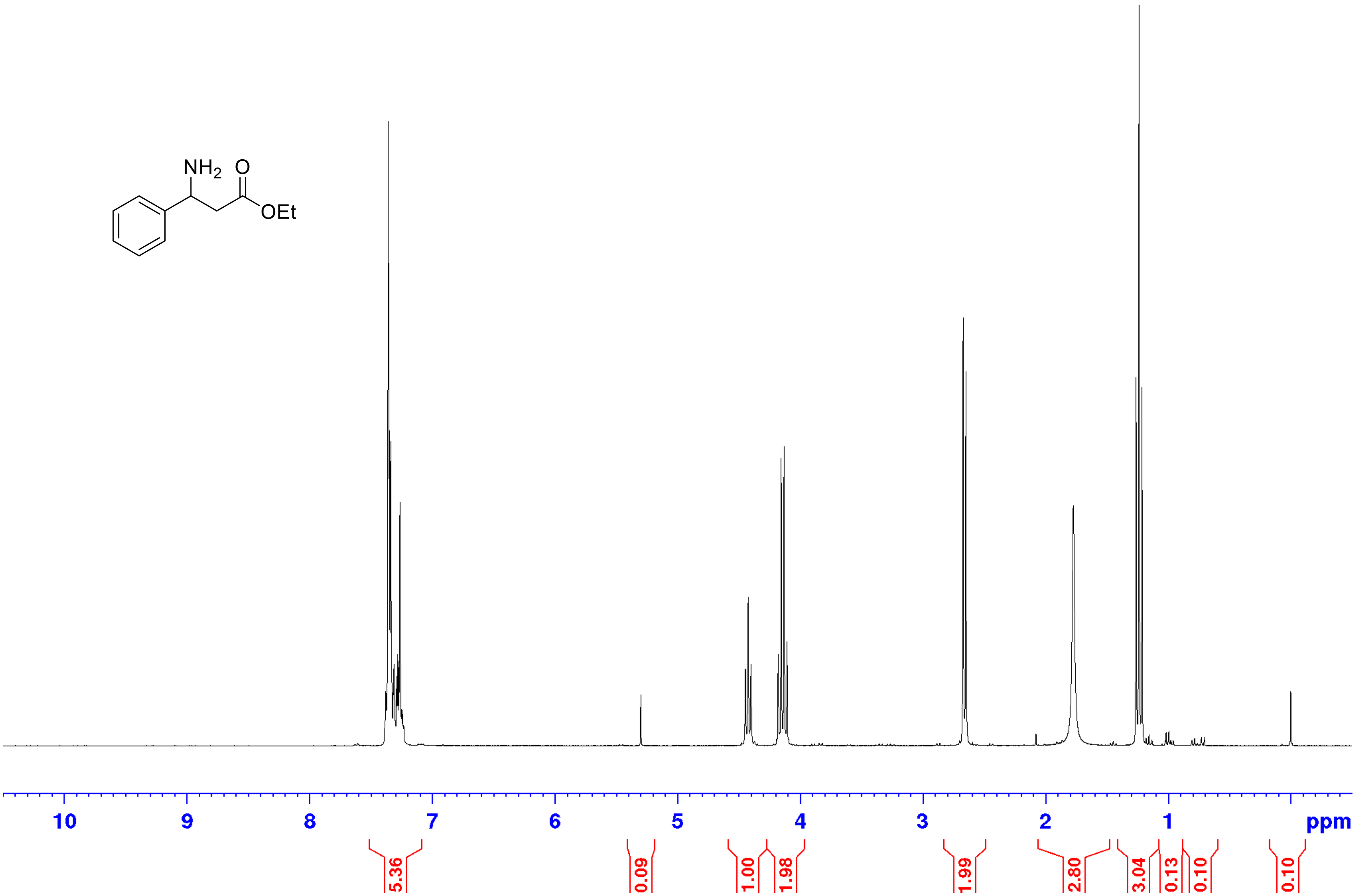
NH2
O
OEt
10
9
8
7
6
5
4
3
2
1
ppm
5.36
0.09
1.00
1.98
1.99
2.80
3.04
0.13
0.10
0.10

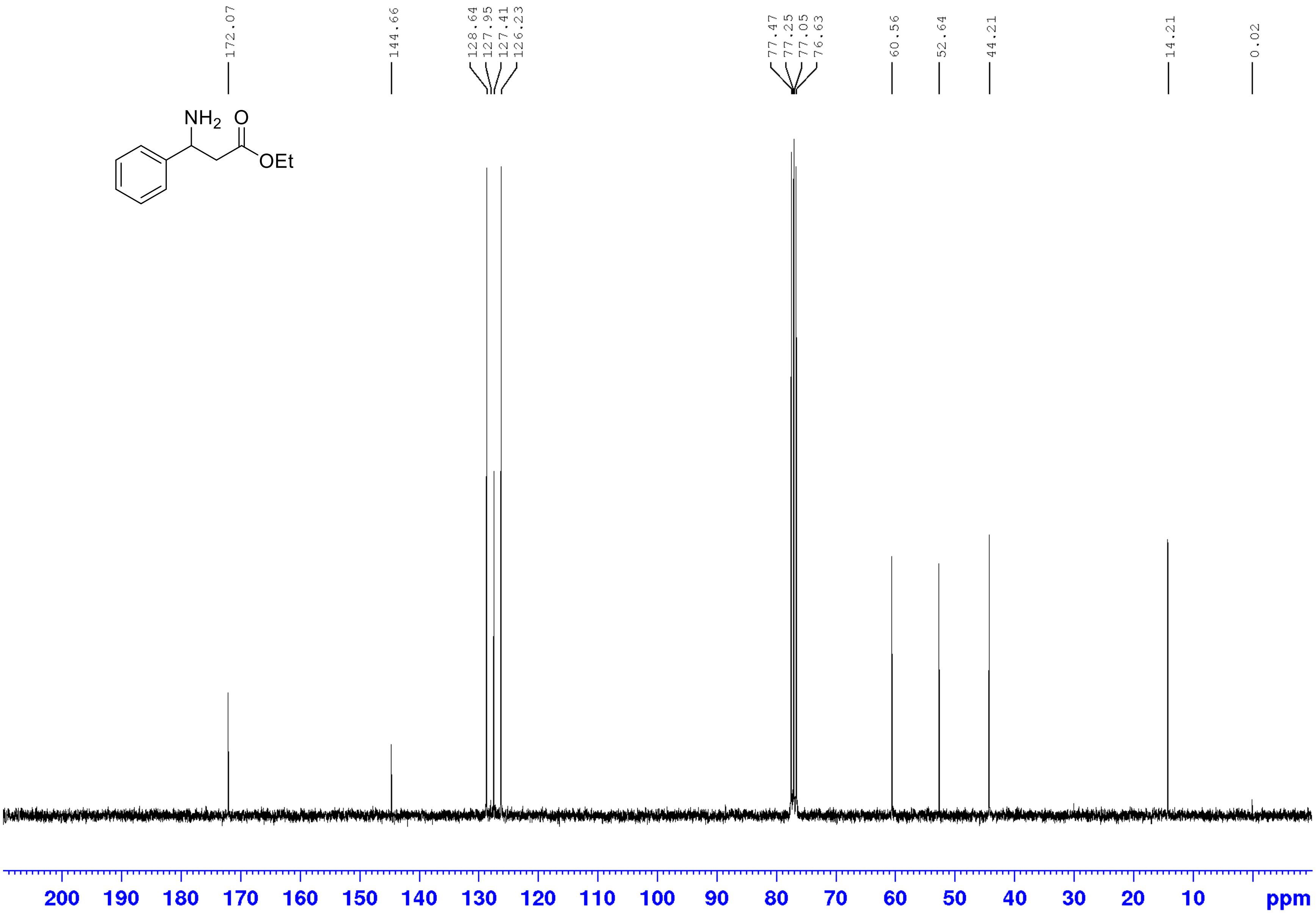

172.07
144.66
128.64
127.95
127.41
126.23
77.47
77.25
77.05
76.63
60.56
52.64
44.21
14.21
0.02
NH_2
O
OEt
200
190
180
170
160
150
140
130
120
110
100
90
80
70
60
50
40
30
20
10
ppm

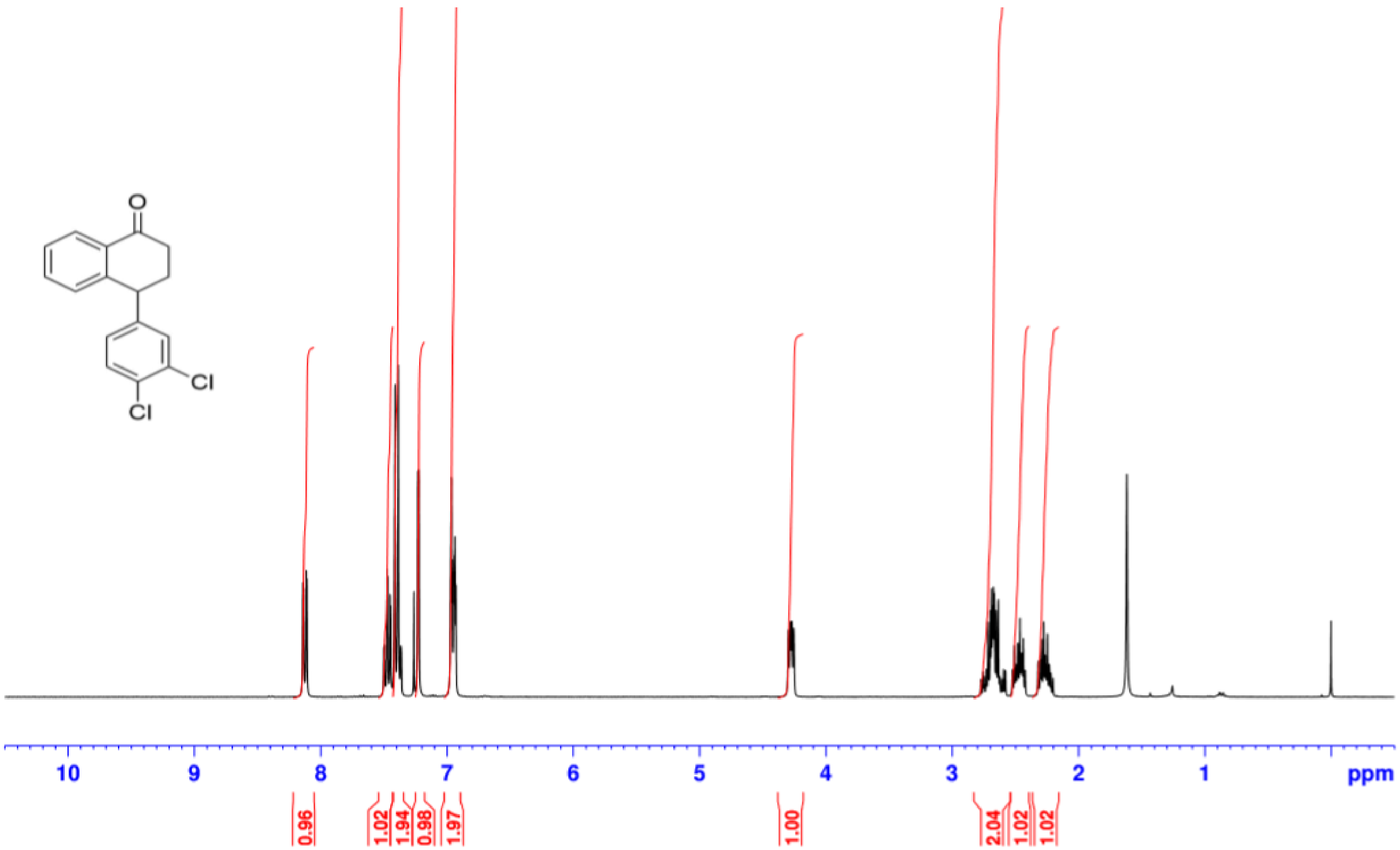
O
Cl
Cl
10
9
8
7
6
5
4
3
2
1
ppm
0.96
1.02
1.94
0.98
1.97
1.00
2.04
1.02
1.02

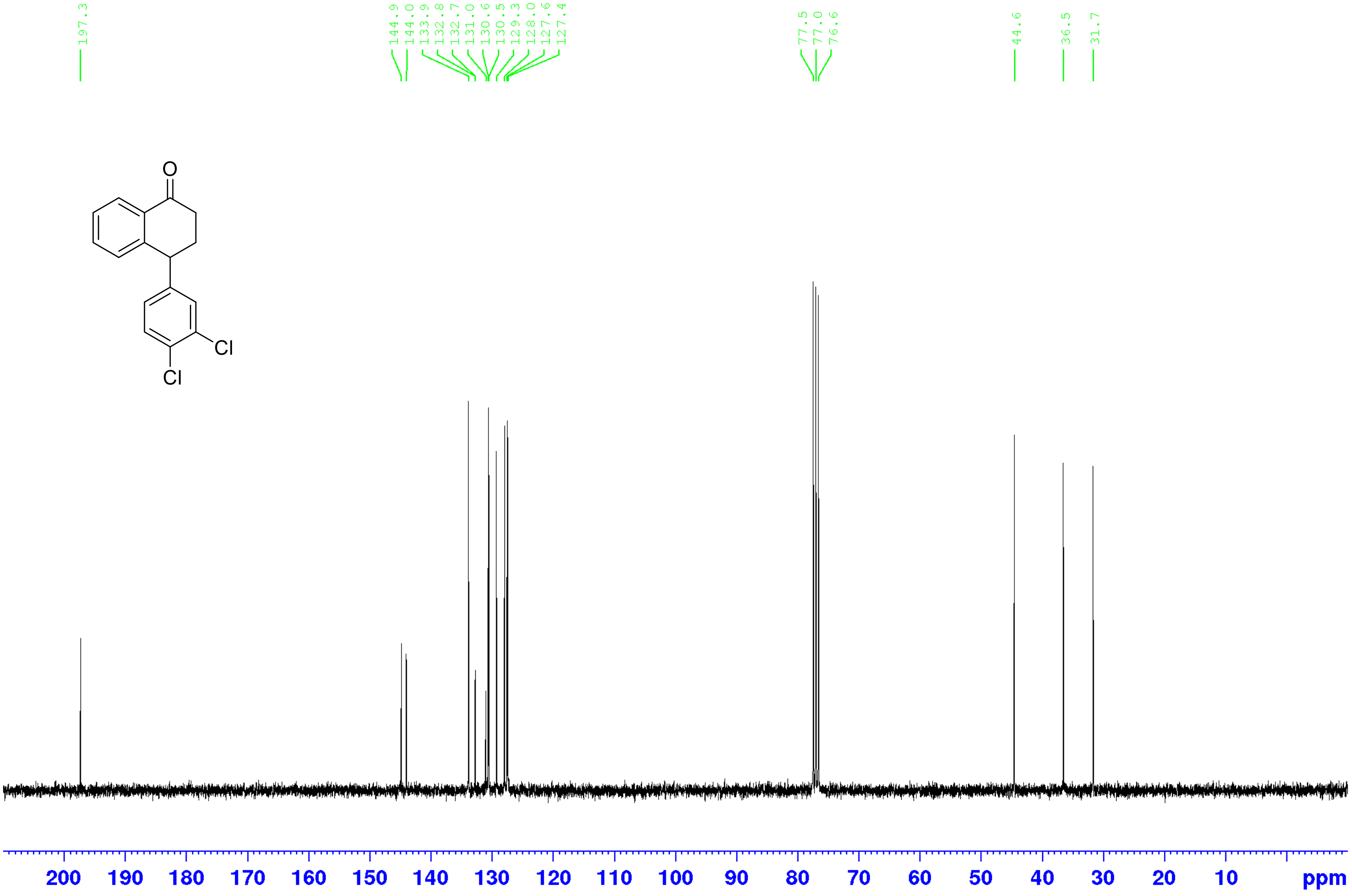
197.3
144.9
144.0
133.9
132.8
132.7
131.0
130.6
130.5
129.3
128.0
127.6
127.4
77.5
77.0
76.6
44.6
36.5
31.7
O
Cl
Cl
200
190
180
170
160
150
140
130
120
110
100
90
80
70
60
50
40
30
20
10
ppm

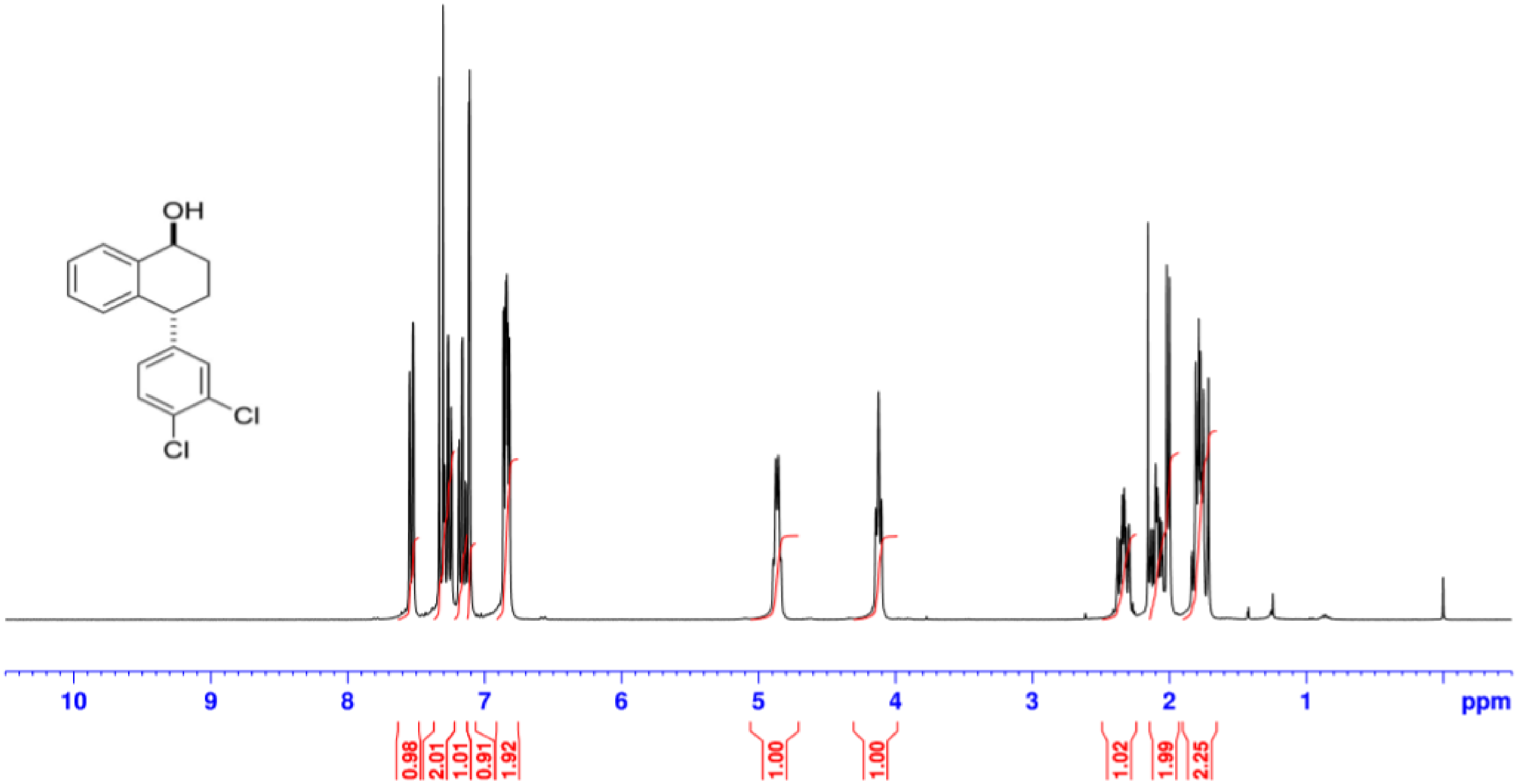
OH
Cl
Cl
10
9
8
7
6
5
4
3
2
1
ppm
0.98
2.01
1.01
0.91
1.92
1.00
1.00
1.02
1.99
2.25

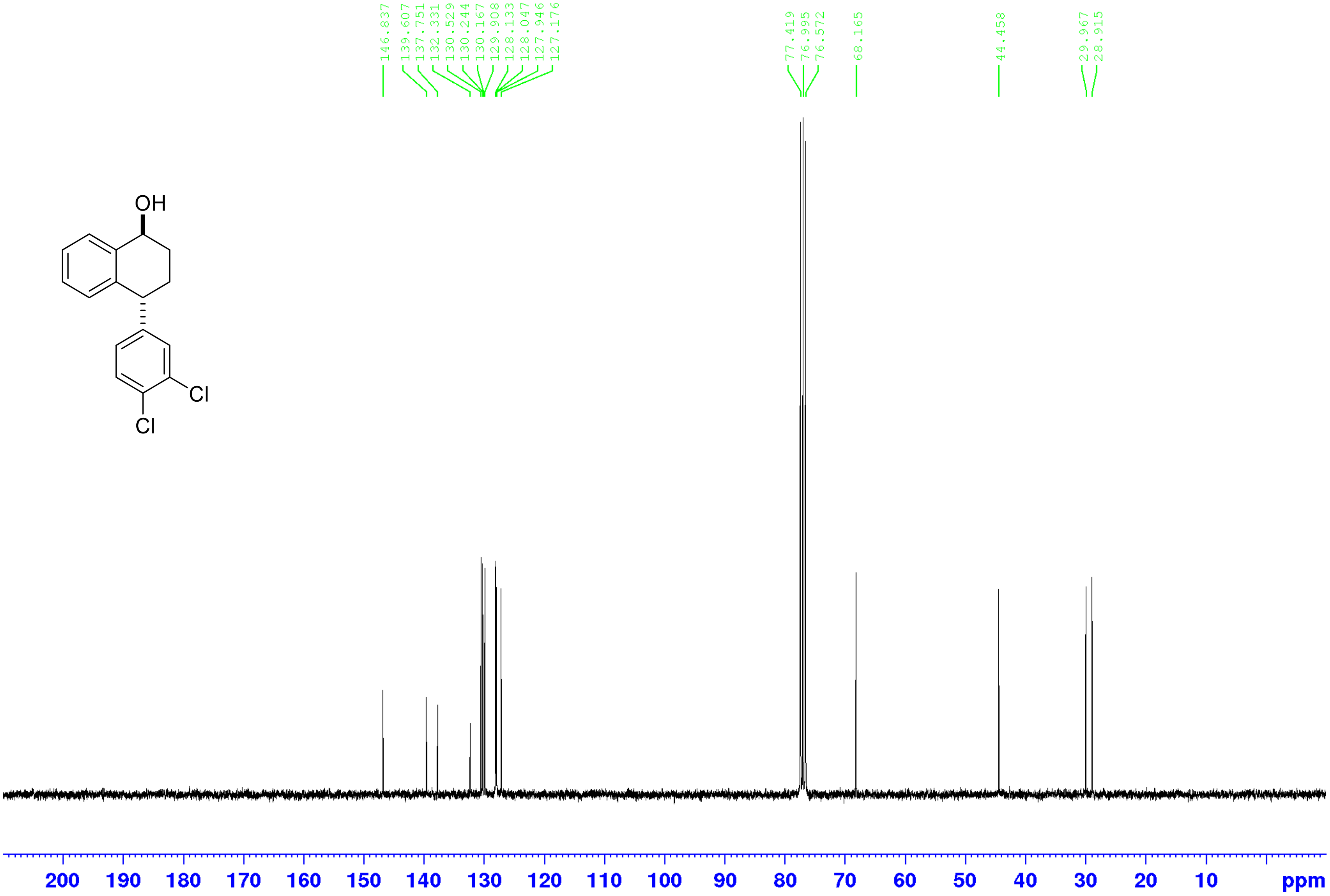
OH
Cl
Cl
146.837
139.607
137.751
132.331
130.529
130.244
130.167
129.908
128.133
128.047
127.946
127.176
77.419
76.995
76.572
68.165
44.458
29.967
28.915
200
190
180
170
160
150
140
130
120
110
100
90
80
70
60
50
40
30
20
10
ppm

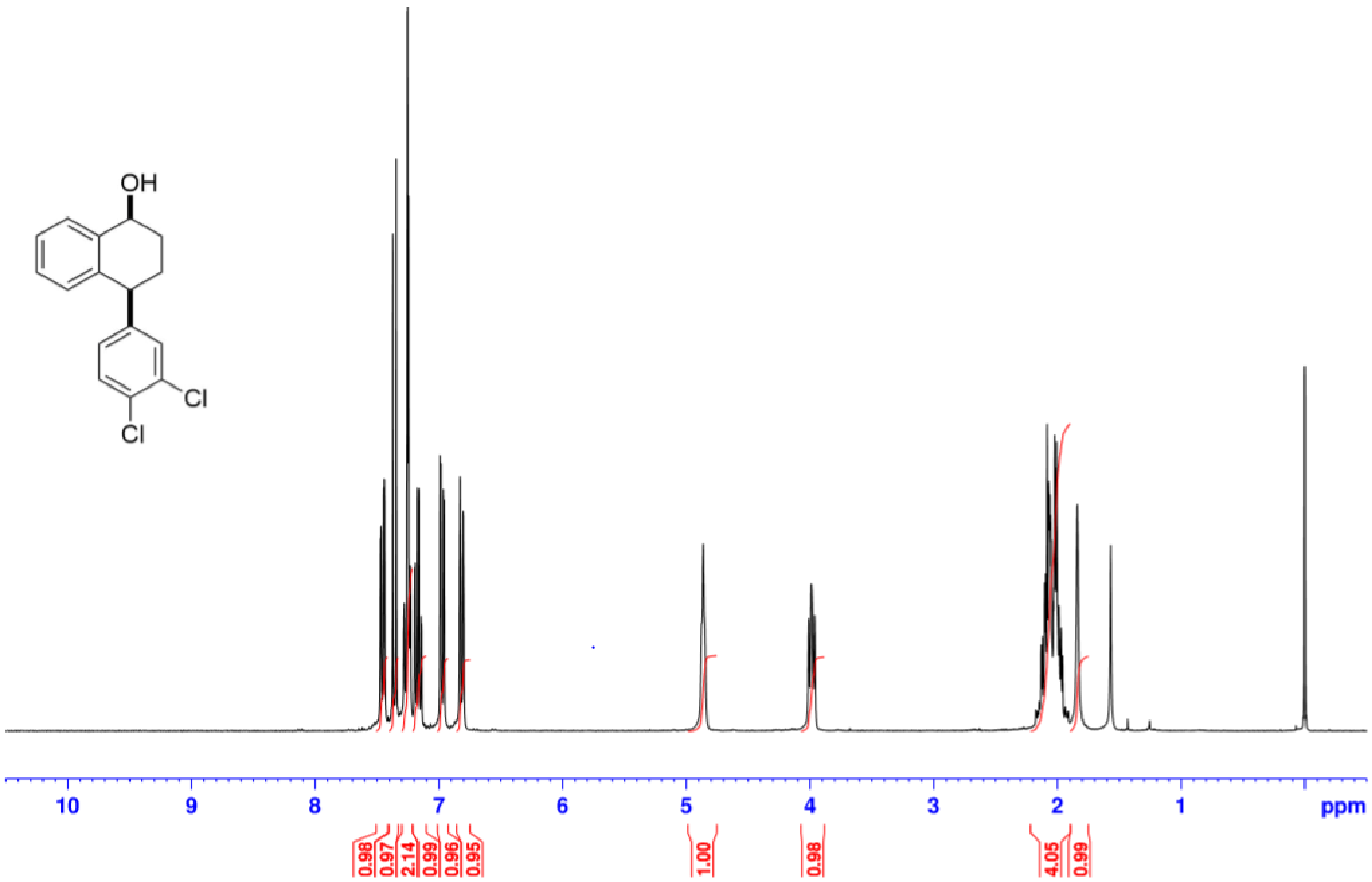
OH
Cl
Cl
10
9
8
7
6
5
4
3
2
1
ppm
0.98
0.97
2.14
0.99
0.96
0.95
1.00
0.98
4.05
0.99

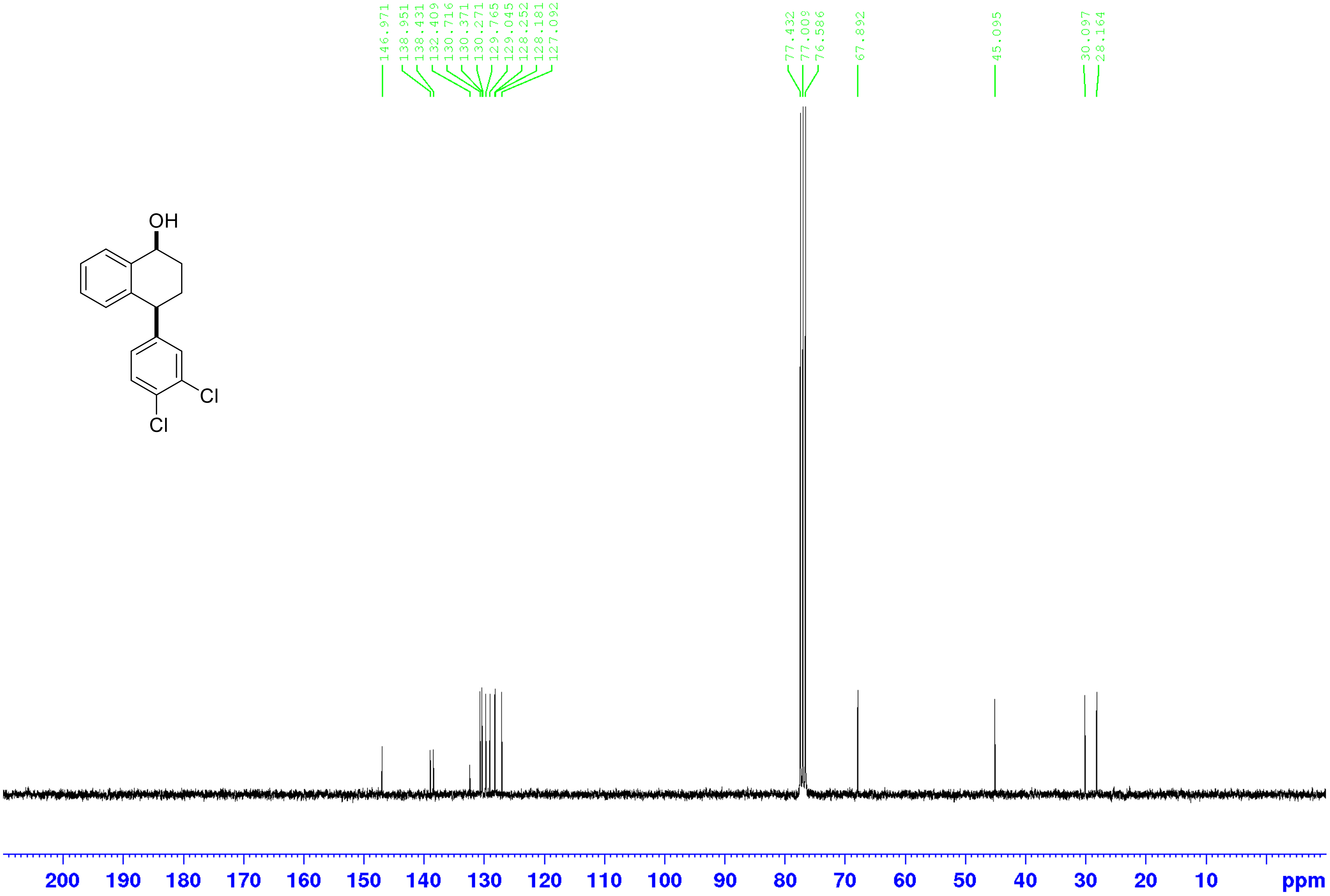
OH
Cl
Cl
146.971
138.951
138.431
132.409
130.716
130.371
130.271
129.765
129.045
128.252
128.181
127.092
77.432
77.009
76.586
67.892
45.095
30.097
28.164
200 190 180 170 160 150 140 130 120 110 100 90 80 70 60 50 40 30 20 10 ppm

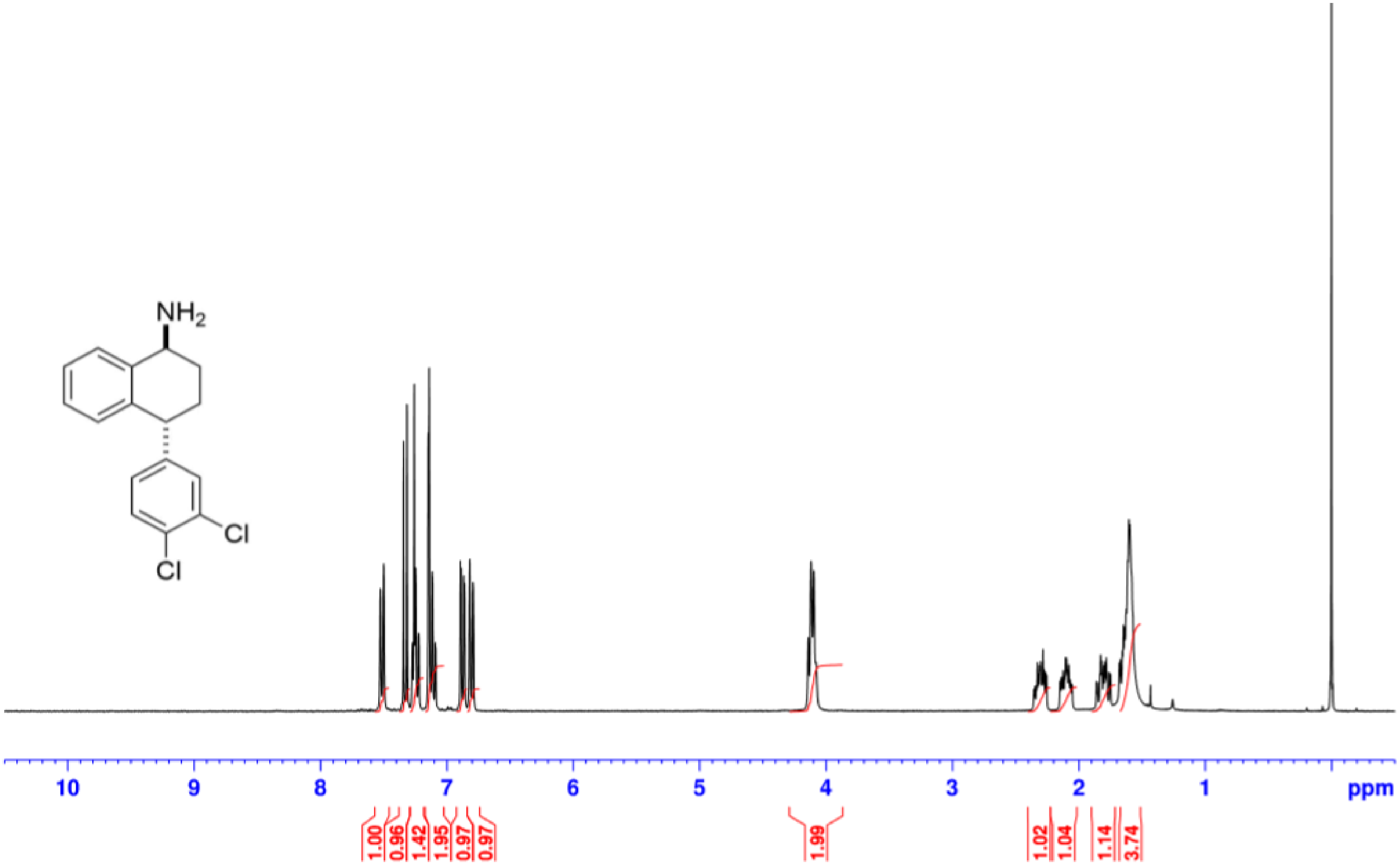
NH2
Cl
Cl
1.00
0.96
1.42
1.95
0.97
0.97
1.99
1.02
1.04
1.14
3.74
10
9
8
7
6
5
4
3
2
1
ppm

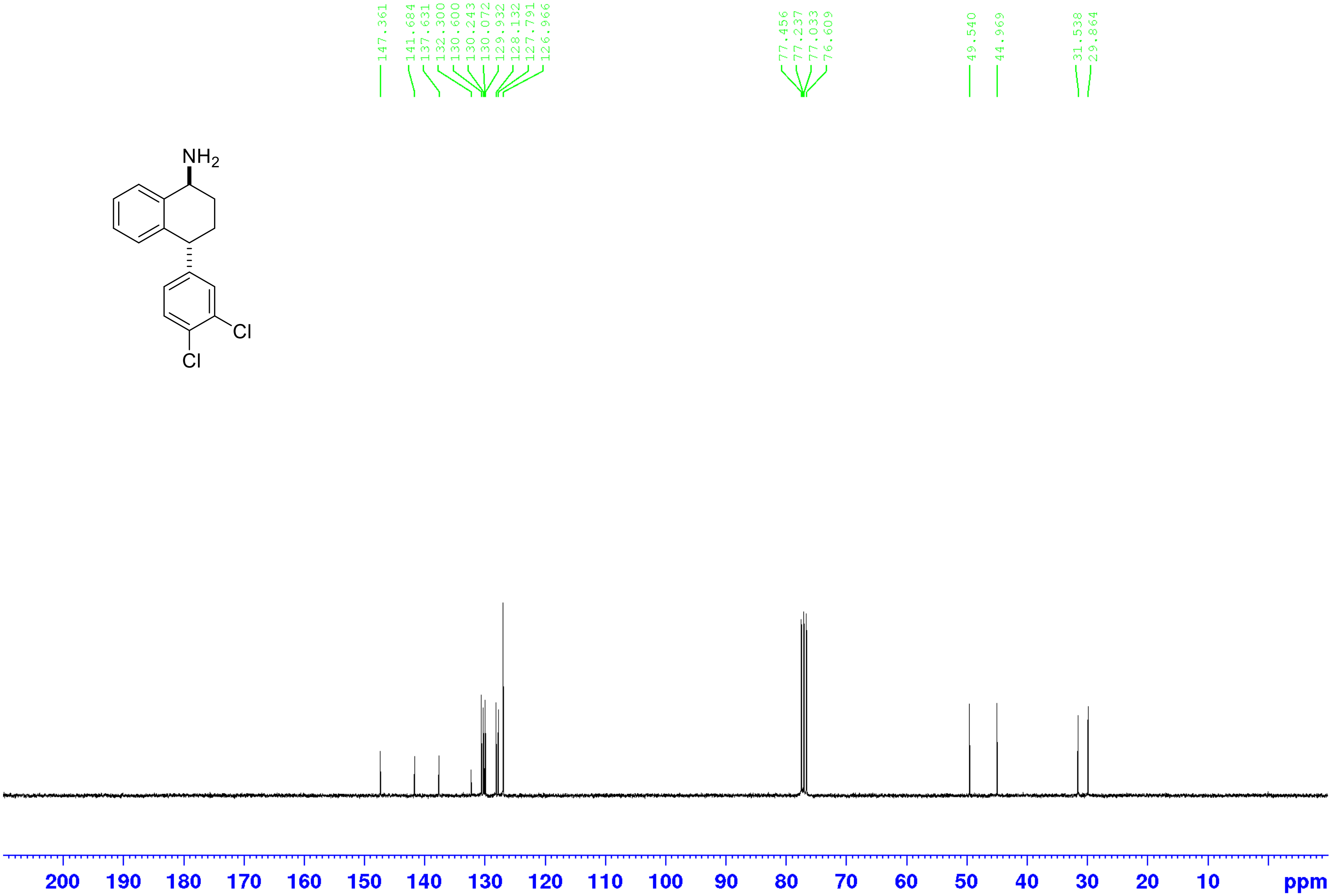
NH2
Cl
Cl
147.361
141.684
137.631
132.300
130.600
130.243
130.072
129.932
128.132
127.791
126.966
77.456
77.237
77.033
76.609
49.540
44.969
31.538
29.864
200
190
180
170
160
150
140
130
120
110
100
90
80
70
60
50
40
30
20
10
ppm

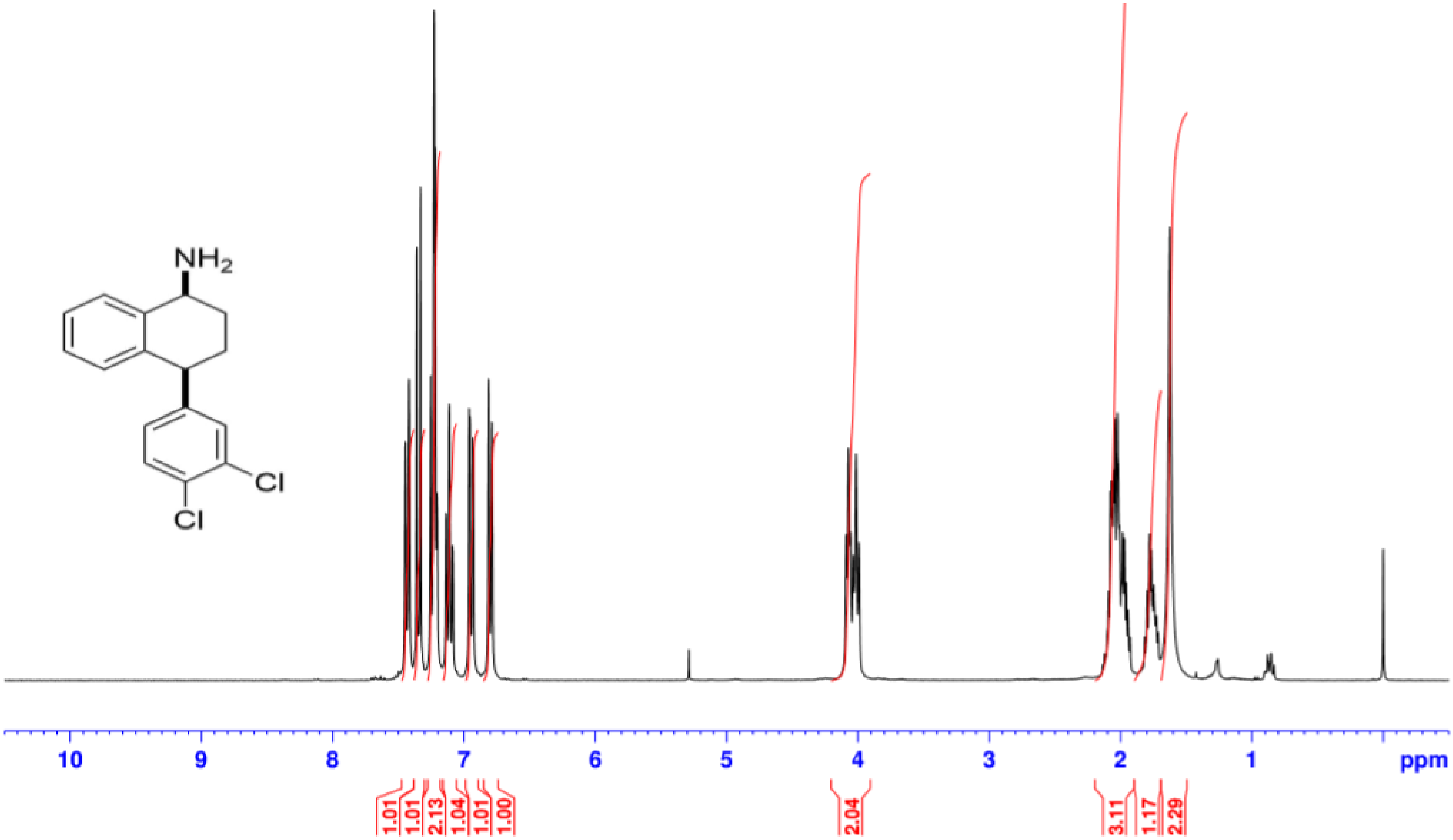
NH2
Cl
Cl
10
9
8
7
6
5
4
3
2
1
ppm
1.01
1.01
2.13
1.04
1.01
1.00
2.04
3.11
1.17
2.29

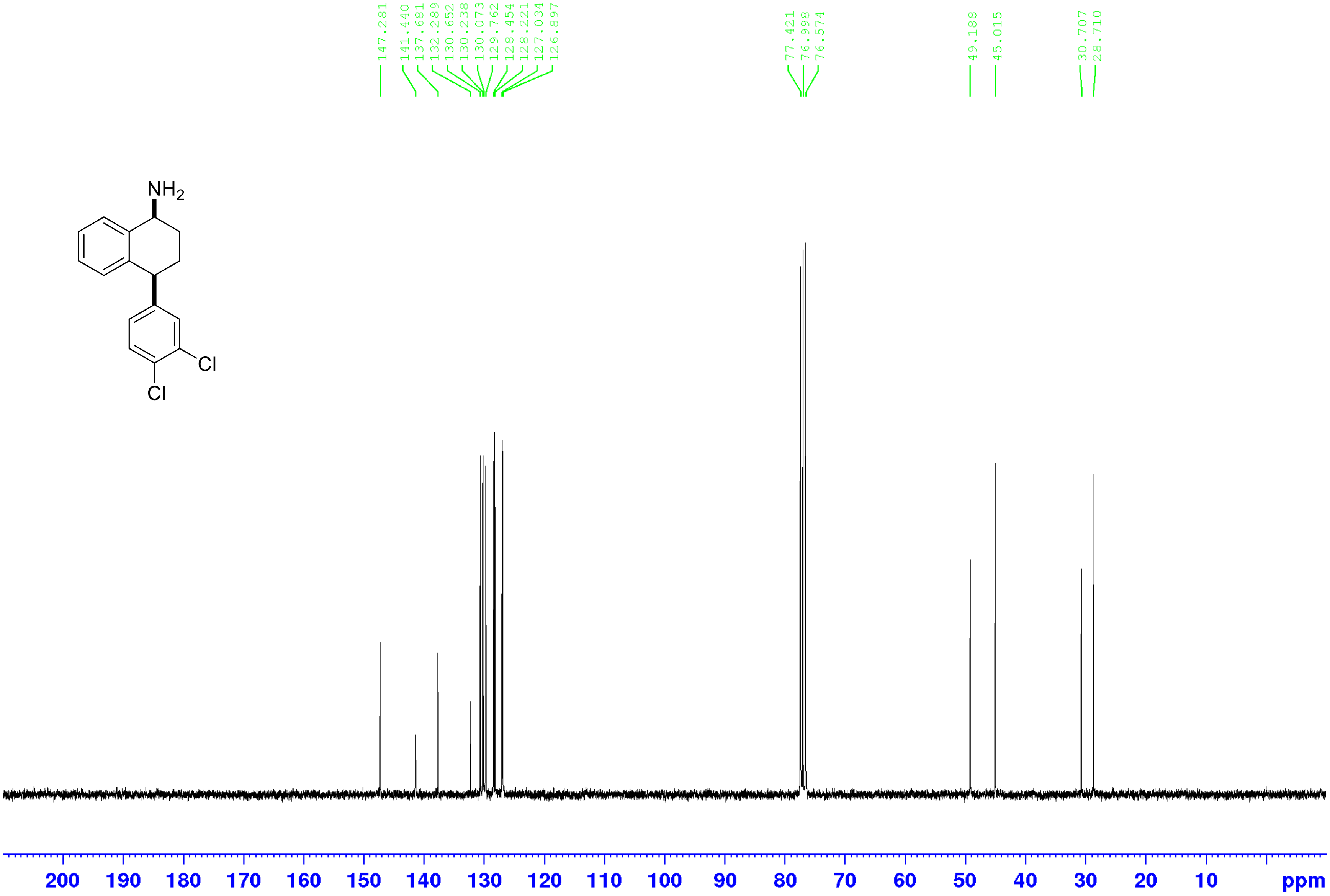
147.281
141.440
137.681
132.289
130.652
130.238
130.073
129.762
128.454
128.221
127.034
126.897
77.421
76.998
76.574
49.188
45.015
30.707
28.710
NH2
Cl
Cl
200
190
180
170
160
150
140
130
120
110
100
90
80
70
60
50
40
30
20
10
ppm
